# Development of Dairy Products Fortified with Plant Extracts: Antioxidant and Phenolic Content Characterization

**DOI:** 10.3390/antiox12020500

**Published:** 2023-02-16

**Authors:** Aikaterini Kandyliari, Panagiota Potsaki, Panoraia Bousdouni, Chrysoula Kaloteraki, Martha Christofilea, Kalliopi Almpounioti, Andreani Moutsou, Chistodoulos K. Fasoulis, Leandros V. Polychronis, Vasileios K. Gkalpinos, Andreas G. Tzakos, Antonios E. Koutelidakis

**Affiliations:** 1 Department of Food Science and Nutrition, University of the Aegean, 81400 Myrina, Lemnos, Greece; 2 Department of Food Science and Human Nutrition, Agricultural University of Athens, 11855 Athens, Greece; 3 Department of Chemistry, University of Ioannina, 45110 Ioannina, Greece; 4 Institute of Materials Science and Computing, University Research Center of Ioannina, 45110 Ioannina, Greece

**Keywords:** phytochemicals, FRAP, LC-MS, bioavailability, organoleptic evaluation, cream cheese, yogurt, kefir, herbs, plant byproducts

## Abstract

In recent decades, there has been growing interest in the fortification of dairy products with antioxidants and phenolics derived from plant byproducts and herbs. The present study focused on the analysis of dairy products, including kefir, cream cheese, yogurt, and vegan yogurt, enhanced with aqueous extracts of plant byproducts (*Citrus aurantium* peel, *Citrus limon* peel and *Rosa canina* seed) and herbs (*Sideritis* spp., *Hypericum perforatum*, *Origanum dictamnus*, *Mentha pulegium* L., *Melissa oficinallis*, *Mentha spicata* L. and *Lavandula angustifolia*) to characterize their antioxidant content, phenolic profile, and organoleptic characteristics. Antioxidant and phenolic content were determined by Folin–Ciocalteu and ferric reducing antioxidant power (FRAP) assays and presented values up to 46.61 ± 7.22 mmol Fe^2+^/L and 82.97 ± 4.29 mg gallic acid (GAE)/g, respectively for the aqueous extracts, as well as up to 0.68 ± 0.06 mmol Fe^2+^/L and 2.82 ± 0.36 mg GAE/g for the fortified dairy products. The bioavailability of antioxidants and phenolics in fortified foods was determined after in vitro digestion and ranged between 4 and 68%. The phytochemical profile of the aqueous extracts was determined by mass spectrometry, and 162 phytochemicals were determined, from which 128 belong to the polyphenol family including flavonoids and phenolic acids. Furthermore, most of the identified compounds have been recorded to possess enhanced antioxidant capacity in correlation to the in vitro findings. Finally, organoleptic evaluation showed an overall acceptability around 3.0 ± 1.0 on a 5-point scale. In conclusion, the studied plants and herbal extracts can be used for the fortification of a variety of dairy products with potential positive effects on human health.

## 1. Introduction

In the last decade, consumer choice has played an important role in shaping food supply chains worldwide. First, there was a shift in consumer preferences from synthetic to natural food ingredients, and at the same time, interest in foods of high nutritional value. This has led the food industry to utilize plant-based products such as fruits, vegetables, herbs, and spices [1,2]. However, the high demand for food products, such as vegetables and fruits, has generated immense amounts of food waste, including peels and seeds, which has created great concern for their management and environmental footprint [3,4]. Research has shown that these plant-derived byproducts constitute an important source of antioxidants, phenolic compounds, dietary fibers, and other bioactive compounds [5,6], and their use as ingredients in food fortification processes and for the development of functional foods has been studied [7,8].

Food can be considered functional if it improves human health [9]. The term was first used in the 1980s by the Ministry of Health and Welfare of Japan and describes food products that maintain beneficial health effects for human functions while also remaining nutritious [10,11]. Bioactive compounds containing antioxidants, polyphenols, carotenoids, fibers, and phytosterols have been used as sources of functional ingredients [12]. Herbs have therefore been characterized as plant based functional foods, as they constitute a great source of antioxidants [13]. As natural antioxidant compounds have been proven to have multifunctional benefits, bioactive compounds derived from herbs and fruits have been used for the enrichment of food products because of their antimicrobial properties, such as flavor, aroma, and color enhancers, and for their therapeutic properties [14,15].

Although many natural byproducts and herbs have been characterized as good sources of natural antioxidants and phenolic compounds, they exhibit diverse nutritional and organoleptic properties. This can vary depending on their origin and cultivation conditions [16,17,18]. Therefore, it is important to evaluate the quality and nutritional value of these sources for their utilization as functional ingredients. In addition, as consumer acceptance depends highly on the sensory characteristics of foods, it is significant to evaluate sensory characteristics and organoleptic properties of the final functional foods.

The growing interest of consumers in healthier food choices has led the food industry to develop various functional foods. Among them, dairy products including yogurt, cheese, and fermented milk have become increasingly popular as functional food matrixes, due to the fact they are being consumed daily and because health-conscious consumers present a preference over fortified dairy products [19,20,21,22]. Aiming to the production of fortified dairy products, plant bioactive extracts have been used for the food industry [19]. Extracts from plant byproducts such as orange peel, lemon peel, and pomegranate peel and herbs, such as basil, sage, and mountain tea, have been used as natural additives in yogurt, cheese, and kefir products to improve their nutritional and health properties [23,24,25]. However, in order for consumers to purchase these functional foods, the nutritional value and sensory characteristics of the products remain crucial aspects [26].

Data on fortified dairy products containing bioactive compounds derived from plant byproducts or herbs are encouraging and provide useful insights for further studies. Herbal extracts have been used to fortify dairy products to increase the antioxidant and total phenolic properties of the products. Although this fortification did not seem to affect the quality of the studied products, no organoleptic studies have evaluated the responses of consumers [22,27,28,29]. In addition, studies have shown that the addition of fruit and vegetable peel extracts to dairy products by microencapsulation improves the nutritional analysis and organoleptic characteristics of the final products [30,31]. However, more research is needed to overcome technological challenges for new fortified dairy products to be produced on a mass scale.

The aim of the present study was to investigate and characterize the antioxidant, phenolic, and organoleptic characteristics of fortified dairy products, including kefir, cream cheese, yogurt, and vegan yogurt enhanced with plant byproducts and herbal extracts. Specifically, in a series of aqueous extracts of bitter orange peel, lemon peel, rosehip seeds, and herbs, such as mountain tea, St. John’s wort, dittany, pennyroyal, lemon balm, spearmint, and lavender, the total antioxidant and phenolic content as well as their phenolic profile were determined, and these extracts were used for the fortification of dairy products. The phytochemical profile of the aqueous extracts was determined by mass spectrometry. Finally, the bioavailability of antioxidant and phenolic content of the newly developed fortified dairy products was determined, and their sensory characteristics were evaluated.

## 2. Materials and Methods

In the context of the present study, an in vitro study was conducted to evaluate the antioxidant capacity, total phenolic content, and phenolic profile of plant byproducts and herbs, which have been associated with high antioxidant activity. In addition, an in vitro study simulating gastrointestinal digestion was carried out to evaluate the antioxidant and phenolic content of the extracts and newly developed dairy functional products. Finally, pilot organoleptic characterizations were performed to investigate consumer acceptance of the newly developed products.

### 2.1. Sample Extract Preparation

Plant byproduct and herb samples were collected from Lemnos Island, North Aegean, Greece, between June and October 2021. The samples consisted of bitter byproducts from orange (*Citrus aurantium*) peel (*n* = 5), lemon (*Citrus limon*) peel (*n* = 5), rosehip (*Rosa canina*) seed (*n* = 5), as well as mountain tea (*Sideritis* spp.) (*n* = 10), St. John’s wort (*Hypericum perforatum*) (*n* = 10), dittany (*Origanum dictamnus*) (*n* = 5), pennyroyal (*Mentha pulegium* L.) (*n* = 10), lemon balm (*Melissa oficinallis*) (*n* = 5), spearmint (*Mentha spicata* L.) (*n* = 10), and lavender (*Lavandula angustifolia*) (*n* = 5). At least 5 samples were used from each food product and pooled together. All samples were dried in a drying heating oven (Binder GmbH, Tuttlingen, Germany) at 60 °C for 12 h and kept in sealed bags until extract preparation.

Extracts were prepared by adding in a flask 2 g of each herb or 10 g of each plant byproduct to 100 mL of dH_2_O. Each flask was then placed in an Elmasonic P 70 H ultrasound water bath (Elma-Hans Schmidbauer GmbH & Co., Singen, Germany) at 70 °C and 80 Hz for 60 min. Filtration of the extracts was performed by filter paper.

### 2.2. Determination of Antioxidant Capacity and Phenolic Content of Sample Extracts

#### 2.2.1. Total Antioxidant Activity by Ferric Reducing Antioxidant Power Assay

Total antioxidant capacity of the food extracts was determined by the ferric reducing antioxidant power (FRAP) assay [32,33,34]. This method was based on the conversion of the TPTZ-Fe^3+^ complex to TPTZ-Fe^2+^, and the absorbance was measured at 595 nm using a SPARK spectrophotometer (TECAN, Zürich, Switzerland). Higher absorbance values were correlated with higher antioxidant capacity by converting the TPTZ-Fe^3+^ complex to TPTZ-Fe^2+^. Quantification of the antioxidant activity was performed using a standard FeSO_4_ curve, and the results of the FRAP assay were quantified in mmol of Fe^2+^ per L of sample extract. The analysis was performed in triplicate. All chemicals were purchased from Sigma-Aldrich (Sigma-Aldrich, St. Louis, MO, USA).

#### 2.2.2. Total Phenolic Content by Folin–Ciocalteu Assay

The total phenolic content of the sample extracts was determined using the Folin–Ciocalteu method. This method is based on the measurement of the reductive capacity of the Folin–Ciocalteu reagent in an alkaline environment, and absorbance was measured at 765 nm using a SPARK spectrophotometer (TECAN, Zürich, Switzerland) [35]. The total phenolic content was determined using a standard gallic acid (GAE) curve, and the results were expressed in milligrams GAE per gram of dried food sample. The analysis was performed in triplicate. All chemicals were purchased from Sigma-Aldrich (Sigma-Aldrich, St. Louis, MO, USA).

### 2.3. Phenolic Profile Determination

#### 2.3.1. Sample Preparation for Phenolic Profile Determination

The sample extracts were prepared according to the methodology described above (Section 2.1). The resulting solution was filtered using a sintered glass filter funnel to remove the solid plant material. The solvent was removed by lyophilization. Phytochemical standards were purchased from Sigma-Aldrich (St. Louis, MO, USA); ethanol and other solvents were purchased from J. T. Baker (J.T.Baker, Randor, PA, USA).

One milligram of each extract was weighed into an Eppendorf tube and was dissolved in 1 mL LC-MS grade water. Samples were further centrifuged at 13,416× *g* for 10 min. Extracts were diluted to 0.2 mg/mL in water and then filtered with a 0.2 μm syringe filter for LC-MS analysis.

#### 2.3.2. Determination of Phenolic Profile with LC-MS/QToF Analysis

LC-MS/QToF analysis of extract was performed on an Xevo-G2-XS-QToF mass spectrometer coupled to a Waters UPLC I-Class Binary Solvent Manager (Waters Corp., Milford, MA, USA). The MS conditions were as follows: the scan range was set at 50–1200 m/z, the source voltage was 1 kV for negative mode and 0.8 kV for positive mode. The source temperature was 120 °C, and the desolvation gas temperature was 550 °C; the flow of desolvation gas (N_2_) was set to 1000 L per hour, and cone gas flow was set to 20 L per hour. For MS/MS, collision energy ramp was set from 20 to 40 eV, and the declustering potential was 40 V. The injection volume was 1 μL. The eluent flow was set to 0.4 mL/min, and the column used was the Acquity UPLC^®^ HSS T3 1.8 μM. More specifically, the elution system comprised 2 phases, A and B. Phase A was water (H_2_O) with 0.1% formic acid (CH_3_COOH), and phase B was acetonitrile (CH_3_CN) with 0.1% formic acid (CH_3_COOH). The gradient was as follows: The run was performed with a constant flow of 400 μL per minute. At the start of the injection, the eluent composition was 1% A that was linearly raised to 100% A by the 10 min mark. This composition (100% A) was held until minute 12.67. The system changed back to initial conditions at minute 12.73 and was held at initial conditions until minute 15, at which point the next injection was performed. The complete chromatographic conditions are summed up in Table S31 from the Supplementary Materials.

For the post processing and analysis of the acquisition data, UNIFY software was used.

### 2.4. In Vitro Digestion Analysis

#### 2.4.1. In Vitro Digestion Reagents and Chemicals

All chemicals were acquired from Sigma-Aldrich (St. Louis, MO, USA) and from Merck Chemicals (Darmstadt, Germany).

#### 2.4.2. In Vitro Gastrointestinal Digestion (GI)

The in vitro gastrointestinal (GI) digestion assay was conducted according to the method proposed by Kapsokefalou et al. with some modifications [34]. More specifically, the extracts were adjusted to pH 2.8 with HCL 1 M. In 6 well plates, 2 mL of each extract was added to each well and mixed with 0.1 mL of human pepsin. The plates were then placed in a shaking incubator (SKI-4, P.R.C.) for 2 h in 37 °C. After incubation, the dialysis membrane was used in well rings, and piperazine-N,N′-bis(2-ethanesulfonic acid) (PIPES) buffer reagent was added to each well to acidify the mixture at pH 6.3. Another incubation was performed (1 h, 37 °C), and then, a mixture of pancreatin-bile acids (0.5 mL) was added to each well and incubated for 2 h followed at 37 °C. The digestion samples were centrifuged for 15 min at 4000 rpm at 4 °C.

### 2.5. Development and Analysis of Fortified Dairy Products with Herbal and Plant Byproduct Extracts

Household utensils and drinking water were used to fortify dairy products. All measures of hygiene and food safety were followed [36]. Dairy products were bought from a local supermarket and included plain kefir milk (Mevgal Kefir, Mevgal, Greece), a spread cream cheese (Philadelphia, Mondelez, Greece), plain Greek yogurt (Total, Fage, Greece), and vegan yogurt (So Soja, Sojade, France). Then, 1 mL of each herbal or plant byproduct extract was added to 20 g of each dairy product, namely the kefir, cream cheese, yogurt and vegan yogurt and mixed well with a spoon. A description scale was used to evaluate the product color, aroma, texture, and flavor. More specifically, the description scale for the intensity of the aroma was defined as ‘not any observed’, ‘low intensity’, ‘intense’ and ‘high intensity’. The liking scale for flavor was coded as ‘not likable’, ‘likable’ and ‘really likable’. To describe the texture of the products, the scale was defined as ‘pleasant’ and ‘not pleasant’. The addition of 1 mL of extracts was then repeated until all organoleptic characteristics of each fortified dairy product were evaluated positively. The final choice and the respective concentration of each extract used for the fortification of the studied dairy products can be found in Section 3.3. The final concentration of the selected extracts was determined in mL/100 g of fortified product.

The new fortified products were then analyzed using an in vitro digestion process as described above, and their total antioxidant capacity, total phenolic compounds, and the respective compound bioavailabilities were determined.

### 2.6. Sensory Evaluation and Organoleptic Characteristics

The present study was approved by the Research Ethics Committee of the University of Aegean, Greece (no. 13, 18 February 2022). All participants provided written informed consent in accordance with the Declaration of Helsinki [37]. Any information that might reveal the identity of the study participants was omitted, and the participants were number coded.

The study was conducted from March to December 2022. Participants were recruited randomly by the research team via social media and online announcements at the Agricultural University of Athens and the University of Aegean, Lemnos, Island, Greece. All participants were randomly selected, and no further educational/informative leaflets were given about the fortified dairy products.

**Figure 1:**
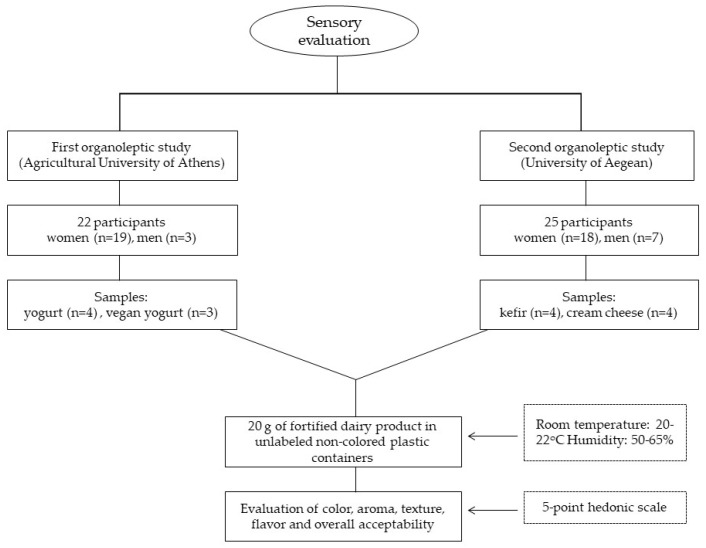
Flow diagram of organoleptic study.

Two organoleptic evaluations were performed as presented in Figure 1. The first organoleptic study was performed in Athens, Greece (Agricultural University of Athens) with a total number of 22 participants being women (*n* = 19) and men (*n* = 3) and the second organoleptic study was implemented in Lemnos Island, Greece (University of Aegean) with a total number of 25 participants, women (*n* = 18) and men (*n* = 7). In both studies, unlabeled non-colored disposable plastic containers with 20 g of fortified dairy product were provided to the participants. The studies were conducted in a room with 20–22 °C temperature and 50–65% humidity. Enhanced dairy products were provided to each of the participants, and they did not have any information on which type of fortified product they were censoring. Furthermore, a questionnaire was administered to each participant to evaluate the appearance, taste, flavor, and smell of the product on a scale from ‘1 = I do not like it’ to ‘5 = I highly like it’. Between each sample, the participants were instructed to drink water to clean their mouth.

During the first organoleptic study, the participants tested and evaluated 4 different yogurt samples and 3 vegan yogurt samples. In more detail, plain yogurt without the addition of any extract, yogurt with St. John’s Wort extract, yogurt with dittany extract, yogurt with pennyroyal, and yogurt with lemon balm extract were provided to each participant. After finishing the evaluation of the above samples, participants then tested and evaluated the vegan yogurt samples: a control without any extract added, a vegan yogurt with spearmint, and a vegan yogurt with lavender. The samples were provided in different orders for each participant.

During the second organoleptic study, the participants tested 4 kefir samples and 4 cream cheese samples. Specifically, a control kefir with no added extract, kefir with bitter orange peel extract, kefir with both bitter orange peel and lemon peel extracts, and kefir with bitter orange peel and rosehip seed extracts were evaluated. After finishing with the evaluation of the above samples, participants proceeded with the evaluation of cream cheese samples: plain cream cheese with no added extract, cream cheese with mountain tea extract, cream cheese with St. John’s Wort extract, and cream cheese with both mountain tea and St. John’s wort extracts. The samples were provided in different orders for each participant.

### 2.7. Statistical Analysis

Statistical analysis was performed using SPSS package, version 16.1 (SPSS Inc., Chicago, IL, USA). Normal distribution of all continuous variables was tested with the parametric Shapiro–Wilk test, and statistical significance was considered at *p =* 0.05. The total antioxidant and phenolic content of the selected food products after all in vitro analyses are expressed as the mean ± standard deviation (SD). ANOVA was used to investigate the differences between the total antioxidant and phenolic content of the herbal and byproduct extracts. Correlations between the total antioxidant and phenolic content of extracts and their respective content after in vitro digestion were evaluated using Pearson’s correlation test. Organoleptic characteristics as assumed by study participants were presented using descriptive statistics, and ANOVA test was implemented to determine significant statistical differences among participants’ preferences for different food products.

## 3. Results

### 3.1. Estimation of Antioxidant and Phenolic Content before and after In Vitro Digestion

Total antioxidant and phenolic content of the selected herbal and plant byproduct extracts before and after in vitro digestion are presented in Table 1. Mean values for total antioxidant capacity of extracts ranged from 2.15 ± 0.17 to 46.61 ± 7.22 mmol Fe^2+^/L. Lemon balm presented the highest total antioxidant activity, followed by spearmint (22.81 ± 1.40 mmol Fe^2+^/L) and a combination extract of bitter orange peel and rosehip seed (21.87 ± 1.41 mmol Fe2+/L). Lemon peel presented the lowest antioxidant activity, followed by rosehip seeds (2.85 ± 0.21 mmol Fe^2+^/L) and bitter orange peel (3.89 ± 0.35 mmol Fe^2+^/L).

Mean values of total phenolic content before digestion varied significantly *(p <* 0.05) from 4.31 ± 0.54 to 82.97 ± 4.29 mg GAE/g of dried sample. Lemon balm had the highest phenolic content, followed by the combination extracts of bitter orange peel and rosehip seed (67.07 ± 1.67 mg GAE/g) and bitter orange and lemon peels (47.92 ± 2.10 mg GAE/g), while the lowest phenolic content was measured in plant byproduct extracts.

After the implementation of a simulated in vitro digestion model, the values of total antioxidant and phenolic content were generally lower, as presented in Table 1. Total phenolic content ranged from 0.71 ± 0.15 to 10.71 ± 1.20 mmol Fe^2+^/L after digestion. Regarding the respective estimated bioavailability of total antioxidants of the selected extracts, the extract with the greatest bioavailability was bitter orange peel (42%), followed by lemon peel (36%), whereas those with lower bioavailability were mountain tea (15%) and the combination of bitter orange peel and rosehip seeds (18%).

Total phenolic content after in vitro digestion ranged from 1.33 ± 0.55 to 9.68 ± 4.31 mg GAE/g. Lemon peel had the highest bioavailability (68%) followed by rosehip seeds (62%), whereas spearmint and pennyroyal had the lowest bioavailabilities (4% and 5%, respectively).

Regarding the correlations between the total antioxidants of the samples before and after the in vitro digestion procedure, only lemon peel, mountain tea, and lavender extracts presented statistically significant correlations (*p <* 0.05). Respectively, in terms of correlations in the phenolic content, prior and after digestion, only dittany extract showed significant correlation (*p <* 0.05).

Table 2 summarizes the study results published in the literature regarding the total antioxidant and phenolic content of the selected plant byproduct and herbal extracts from the Mediterranean region, as measured with FRAP and Folin–Ciocalteu assays. As presented in Table 2, there is a wide range of values between the different studies.

### 3.2. Determination of Phytochemical Profile of Aqueous Plant Byproduct and Herbal Extracts

To unveil the phytochemical profile of the aqueous extracts of the studied plant materials, LC-MS QTOF spectrometry was performed. The phytochemical profile of the 9 studied plants was identified and a total of 162 different phytochemicals were determined, out of which 128 belong to the polyphenol family (as can be seen in Table S1 of the Supplementary Materials). Each plant was evaluated in two modes (positive and negative), and thus, 18 recordings were performed, and identification data are presented in the Supplementary Materials (Tables S2–S19). The most abundant phytochemicals in bitter orange extracts, namely, were: echinacoside cirsimaritin, leucosceptoside a, luteolin 7-o-rutinoside, 1,2-disinapoylgentiobiose, rutin, orientin, sucrose, kaempferol, 3-o-sophoroside, 5,7-dihydroxychromone, eupatilin, didymin, limonin, nobiletin, rhoifolin, eriocitrin, apigenin-7-o-glucoside, isorhamnetin-3-o-rutinoside, isorhamnetin, 3-o-galactoside, citric acid, luteolin, 7-o-diglucuronide, quercetin-3-o-glucoside, myricetin, 3-alpha-l-arabinopyranosideand manghaslin.

For the dittany extracts, the main compounds were: rosmarinic acid, salvianolic acid c, lithospermic acid b, vanillylmandelic acid, ferulic acid-4′-o-glucoside, diosmin, salvianolic acid b, isoacteoside, 6″-o-malonylgenistin, naringenin 7-o-glucoside, cirsilineol, glucogallin, cafestol, (2-hydroxy-) rutin, 3,4-dicaffeoylquinic acid, 5-feruloylquinic acid, plumieride, orientin, scutellarin, eupatorine and luteolin 7-o-diglucuronide.

As for the lavender aqueous extracts we detected, among others, the following phytochemicals were: rosmarinic acid, luteolin-3-o-glucuronide, vanillylmandelic acid, quercitrin, apigenin, ferulic acid-4′-o-glucoside, salvianolic acid b, luteolin 7-o-diglucuronide, luteolin, melittoside, theaflavin 3-o-gallate, quercetin 3′-o-glucuronide, chicoric acid, scutellarin, salvianolic acid c, 5-feruloylquinic acid, astragalin, 5-o-caffeoylquinic acid, luteolin 7-o-diglucuronide, cynarin, apigenin-7-o-glucoside, pinoresinol-4-o-beta-monoglycoside, acteoside, caffeoyl tartaric acid and hispidulin glucuronide.

Meanwhile, lemon balm produced extracts rich in: rosmarinic acid, lithospermic acid b, vanillylmandelic acid, quercitrin, diosmetin, salvianolic acid b, nicotiflorin, quercetin 3-o-beta-d-glucopyranosyl-7-o-alpha-l-rhamnopyranoside, 1,3-dicaffeoylquinic acid, leucosceptoside a, salvianolic acid a, luteolin, genkwanin, theaflavin 3-o-gallate, chicoric acid, luteolin 7-o-diglucuronide, echinacoside, silydianin, achillolide a, manghaslin, luteolin 7-o-glucoside and silybin.

Lemon peel extracts contained mainly: quercetin-3-o-glucoside, allobetonicoside, geniposidic-acid, barbatoside, luteolin 7-o-glucoside, rutin, limonin, rosmarinic acid, isorhamnetin 3-o-glucoside, azadirachtin, isorhamnetin 3-o-galactoside, chrysoeriol, hesperidin, quercetin 3-o-(6-malonyl-glucoside), quercetin 3-o-beta-d-glucopyranosyl-7-o-alpha-l-rhamnopyranoside, nobiletin, 1,3-dicaffeoylquinic acid, 4-o-caffeoylquinic acid, byakangelicin, myricetin-3-o-α-l-rhamnopyranoside, cirsimaritin, eriocitrin, astilbin, isorhamnetin-3-o-rutinoside and peonidin 3-o-sophoroside.

Roseship admittedly gave a poorer profile: ascorbic acid, apigenin 7-o-diglucuronide, melittoside, valoneic acid, dilactone 5-o-galloylquinic acid, astilbin, taxifolin, protocatechuic acid 4-o-glucoside, nicotiflorin, diosmin, quercetin 3-arabinoside, dihydroferulic acid 4-o-glucuronide, dihydroferulic acid-4′-o-glucuronide, teupolioside alpha-methyl-d-mannopyranoside and genistein 4′,7-o-diglucuronide 6″-o-malonylgenistin.

As expected, sisderitis produced rich extracts: geniposidic-acid, bergenin, teupolioside, leucosceptoside A, lithospermic acid B, 5-feruloylquinic acid, apigenin, kaempferol 3-O-sophoroside, acteoside, hesperidin, luteolin 7-O-diglucuronide, luteolin 7-O-diglucuronide, citric acid, oleuropein, isoacteoside, 1,3-dicaffeoylquinic acid, apigenin-7-O-glucoside, isoscutellarein 4′-methyl ether 7-(6‴-acetylallosyl)(1->2)-glucoside, isoscutellarein 7-O-[6‴-O-acetyl-β-d-allopyranosyl-(1→2)]-β-d-glucopyranoside and cirsilineol.

Spearmint also gave a rich phytochemical profile including, mainly: oleuropein, luteolin, allobetonicoside, 1,3-dicaffeoylquinic acid, luteolin 7-o-glucoside, vanillylmandelic acid, rutin, rosmarinic acid, lithospermic acid b, isorhamnetin 3-o-glucoside, apigenin-7-o-glucuronide, apigenin 7-o-diglucuronide, salvianolic acid a, melittoside, eupatilin, acteoside, manghaslin (quercetin 3-2 g-rhamnosylrutinoside), hesperidin, luteolin 7-o-diglucuronide, kaempferol 3-o-rutinoside, scutellarin, rhoifolin, 3,4-dicaffeoylquinic acid, diosmin, luteolin-3-o-glucuronide and luteolin 7-o-rutinoside.

Lastly, St. John’s wort gave mostly the following compounds: oleuropein, quercetin-3-o-glucoside, allobetonicoside, astragalin, rutin, amentoflavone, apigenin-7-o-glucuronide, kaempferol, cinnamtannin a2, quercetin, chicoric acid, apigenin-7-o-glucoside, luteolin 4′-glucoside, quercetin 3,4′-o-diglucoside, myricetin-3-o-galactopyranoside, kaempferol-3-o-glucuronide, hyperoside, phloridzin, caffeic acid, myricetin-3-o-α-l-rhamnopyranoside, betonicine, pinoresinol-4-o-beta-monoglycoside and quercetin 3-glucuronate.

Some phytochemicals were identified in both modes and/or in more than one studied plant material. These compounds, which were identified more than once, as well as their identification frequency can be seen in Table 3. Some of the most frequently identified compounds within the samples include luteolin 7-o-diglucuronide, salvianolic acid b, rutin, acteoside, nicotiflorin, chrysoeriol 7-o-apiosyl-glucoside, pinoresinol-4-o-beta-monoglycoside, naringenin-4′,5-diglucuronide, cirsilineol and vanillylmandelic acid.

Out of the 162 different phytochemicals identified, 128 belong to the polyphenol family (Table S1). Based on the number of polyphenolic components, the nine plants were ranked in the following order: 1. bitter orange (52 polyphenols), 2. dittany (41 polyphenols), 3. lemon peel (40 polyphenols), 4. spearmint (39 polyphenols), 5. St. John’s wort (32 polyphenols), 6. lemon balm (31 polyphenols), 7. lavender (29 polyphenols), 8. sideritis (27 polyphenols) and 9. rosehip (13 polyphenols) (Table S30). This order was consistent with the total number of phytochemicals identified in each plant (Table S1). In addition, the number of polyphenolic components in spearmint, St. John’s wort, lavender, sideritis and rosehip is in accordance with the Folin–Ciocalteu assay values.

Sideritis, spearmint, dittany, lavender and lemon balm belong to the Lamiacea family and were screened for compounds that were constitutively shared among them. The analysis of those plants showed that the most common antioxidants in the Lamiaceae family samples were luteolin 7-O-diglucuronide, acteoside, vanillylmandelic acid, scutellarin, salvianolic acid B, lithospermic acid B, rosmarinic acid, 3,4-dicaffeoylquinic acid, luteolin, chicoric acid, 5-feruloylquinic acid, apigenin-7-O-glucoside, nicotiflorin, pinoresinol-4-O-beta-monoglycoside, leucosceptoside A, pectolinarigenin, silybin, rutin, hesperidin, oleuropein, 1,3-dicaffeoylquinic acid, diosmin, kaempferol 3-O-rutinoside, salvianolic acid C, cirsilineol, theaflavin 3-O-gallate, luteolin 7-O-glucoside, cynarin. The most frequently identified antioxidants in the Lamiacea family are shown in Table 4.

Bitter orange and lemon peel belong to the Rutaceae family and were screened for compounds that were constitutively shared among them. The most abundant antioxidants in the Rutaceae family were rutin, limonin, eriocitrin_1, isorhamnetin-3-O-rutinoside, nicotiflorin, orientin, kaempferol 3-O-sophoroside, isorhamnetin 3-O-galactoside, citric acid, didymin, cirsimaritin, pinoresinol-4-O-beta-monoglycoside, astilbin, nobiletin, D-(+)-mannose, azadirachtin, quercetin-3-O-glucoside, allobetonicoside, rhoifolin, eupatilin, 1,2-disinapoylgentiobiose and hesperidin. The most frequently identified antioxidants in the Rutaceae family are shown in Table 5.

The total number of different identified phytochemicals for each plant studied was as follows: 65 different compounds were identified in bitter orange, 51 in dittany and lemon peel, 47 in spearmint, 42 in lavender, 39 in lemon balm, sideritis and St. John’s wort and only 20 in rosehip (Table S20). Bitter orange showed the richest phytochemical profile followed by dittany and lemon peel, while rosehip presented the poorest profile. Based on the number of their antioxidant components recorded (Tables S21–S29), the nine plants were ranked in the following order: 1. bitter orange (44 antioxidants), 2. dittany (37 antioxidants), 3. lemon peel (31 antioxidants), 4. spearmint (30 anti-oxidants), 5. lavender (26 antioxidants), 6. lemon balm (25 antioxidants), 7. sideritis (24 antioxidants), 8. St. John’s wort (20 antioxidants) and 9. rosehip (12 antioxidants). This order is consistent with the total number of phytochemicals identified in each plant material.

Some of the most commonly found antioxidants among the studied plant byproduct and herbal extracts include kaempferol 3-o-sophoroside, isorhamnetin 3-o-galactoside, luteolin 7-o-diglucuronide, salvianolic acid b, rutin, luteolin-3-o-glucuronide, quercetin 3-arabinoside and isoscutellarein 7-o-[6‴-o-acetyl-β-d-allopyranosyl-(1→2)]-β-d-glucopyranoside.

### 3.3. Estimation of Total Antioxidant and Phenolic Content in Fortified Foods after In Vitro Digestion

Total antioxidant capacity and total phenolic content of the above-selected extracts were determined in specific food samples, namely kefir, cream cheese, yogurt, and a vegan yogurt, after simulation of in vitro digestion. The values of dairy products fortified with the respective extracts are presented in Table 6.

The antioxidant content was higher in kefir fortified with bitter orange and rosehip extract, compared to plain kefir (control sample). No statistically significant difference (*p >* 0.05) was observed between the different kefir samples in terms of phenolic content.

Cream cheese showed higher antioxidant and phenolic content when fortified with St. John’s wort extract, with a value of 0.53 ± 0.16 mmol Fe^2+^/L and 56.36 ± 7.13 mg GAE/g, respectively. A statistically significant difference (*p <* 0.05) was observed in both antioxidant capacity and phenolic content.

In yogurt, antioxidant capacity was higher when fortified with lemon balm (1.21 ± 0.12 mmol Fe^2+^/L) yogurt, while no statistical important differences were observed in the phenolic content of samples (*p >* 0.05).

Vegan yogurt fortified with spearmint showed the highest antioxidant activity (0.32 ± 0.04 mmol Fe^2+^/L), while vegan yogurt fortified with lavender and spearmint presented higher phenolic content (2.01 ± 0.40, 2.00 ± 0.41 mg GAE/g, respectively) compared to control sample.

### 3.4. Evaluation of the Organoleptic Characteristics of the Fortified Food Products

The evaluation of the organoleptic characteristics (color, aroma, texture, and flavor) of the fortified food products are presented in Table 7. The ratings were on a five-point hedonic scale: 5 (liked a lot) to 1 (did not like at all).

As presented in Table 4, values for the overall acceptability of kefir ranged from 3.2 ± 1.1 in control sample (plain kefir) to 2.6 ± 1.2 in fortified bitter orange and lemon peels kefir. However, no statistically significant difference (*p >* 0.05) was observed between the samples.

Regarding cream cheese, total acceptability was 4.0 ± 1.1 for control and 2.6 ± 1.0, the lowest value, for cream cheese fortified with mountain tea extract. In addition, a statistically significant difference (*p <* 0.05) was observed between the samples for texture, flavor, and overall acceptability, but not for color and aroma (*p >* 0.05).

Yogurt’s overall acceptability ranged from 3.9 ± 0.8 for the blank yogurt to 3.2 ± 0.9 for yogurt with dittany extract. Statistically significant difference (*p <* 0.05) were presented for color, texture, flavor, and overall acceptability but not for aroma (*p >* 0.05)

As for the vegan yogurt, the highest value (2.8 ± 1.2) for total acceptability was that without any extract, and the lowest value (2.4 ± 1.1) was that with the lavender extract. A statistically significant difference (*p <* 0.05) was presented between samples only for texture.

In general, in all cases, products without any extract had the highest value for overall acceptability.

## 4. Discussion

Several studies have been conducted on herbs and their extracts with the aim of determining their antioxidant capacity and total phenolic content and characterizing the respective phenolic components [58]. However, variations exist, even among the same plant byproducts and/or species. This is attributed to differences in the extraction method during sample preparation [59], differences in harvest time [60], differences in the variety of the analyzed sample [61], as well as differences in the climatic and soil conditions and their origins [62]. Therefore, it remains important to determine the antioxidant and phenolic content of plants cultivated in different places. In the present study, we focused on the North Aegean region and evaluated the total antioxidant and phenolic content of selected aqueous extracts of herbs, byproducts, and several combinations of them.

Among them, lemon balm presented the highest value for antioxidants and total phenolics before and after in vitro digestion; however, it had lower bioavailability compared to other herbal extracts. In general, a decrease in the concentrations of both antioxidants and phenolics was observed in all extracts after simulation of gastrointestinal digestion. This could be due to the degradation of some polyphenols and flavonoids during the transition from acidic gastric conditions to mildly alkaline intestinal conditions, especially under the impact of bile acids and pancreatin [63,64]. Specifically, it is suggested that approximately 15% of polyphenols are lost during the transition from the acidic gastric environment to the mild alkaline intestinal environment [65].

The bioavailability of polyphenols has been a major problem limiting their use as a dietary intervention for different disease factors that polyphenols have been found to prevent or manage [66]. It has been estimated that only 5–10% of the total polyphenol intake is absorbed in the small intestine, whereas the remaining polyphenols (90–95% of total polyphenol intake) may accumulate in the lumen of the large intestine [67,68,69,70]. However, the poor bioavailability of polyphenols favors interactions with gut microbes, whereas microbes may modulate the activity of polyphenols by converting naturally occurring polyphenols into metabolites that can exert different effects [71]. In the large intestine, colonic bacteria act enzymatically on the polyphenolic backbone of the remaining unabsorbed polyphenols (90–95% of the total polyphenol intake), sequentially producing metabolites with different physiological significance [72]. This suggests that biοaccessibility of phenolics may be an important factor in bioavailability, and in the case of food fortification, the concentration of polyphenols in the fortification extracts must be examined alongside the bioavailability rates.

Of the total phytochemicals identified in the studied samples (162), 119 were found to have some level of antioxidant activity according to the published literature [73,74,75,76,77,78,79,80,81,82,83]. Furthermore, 86 of these 119 compounds were identified in more than one plant, and the extracts from the studied plants showed satisfactory results for the bioavailability of total antioxidants (>20%) and total phenolics (>10%). Therefore, we can assume that the studied extracts can be used as potential sources of antioxidants and phenolic compounds for the enrichment of dairy products, which are beneficial to human health [84]. Furthermore, the data show that the greatest benefits of antioxidant and phenol bioavailability can be obtained when they are consumed in food products rather than in supplement forms [85].

According to the results obtained from the FRAP assay for total antioxidant content, lemon balm had the highest antioxidant activity, although it was ranked low in terms of the number of its identified antioxidants. A plausible explanation for this could be that in the specific LC-MS set-up, the recorded phytochemical profile is qualitative, while the results of the FRAP assay are quantity-dependent. The total antioxidant activity depends not only on the total number of different antioxidants (qualitative information) but also on their concentration (quantitative information) and individual antioxidant capacity [86].

Concerning the phenolic profile of the studied plant materials, some results of the Folin–Ciocalteu assay were not in complete accordance with the total number of polyphenolic components. Bitter orange, dittany, and lemon peel were found to contain the most polyphenols out of the nine plant species but gave only poor results when tested using the Folin–Ciocalteu assay. On the other hand, lemon balm showed great results with the Folin–Ciocalteu assay although it was ranked only sixth according to the identified polyphenols (Table S30). These differences may be attributed to the fact that the Folin–Ciocalteu assay is quantity-dependent [87], while as mentioned above, the results of the specific LC-MS set-up are qualitative. Furthermore, the Folin–Ciocalteu assay is based on a redox reaction in which a chromophore complex is formed [88]. Except for polyphenols, numerous reducing agents can produce the complex and thus lead to ambiguous results regarding the total phenolic capacity [89,90].

As suggested by the above, the use of plant byproduct and herbal extracts for fortifying of dairy products has been of great interest in recent years [27,91]. At the same time, dairy products remain high in consumer preferences [21,92], whereas dairy products derived from fermentation are an ideal basis for the incorporation of bioactive ingredients [29]. The use of plant extracts for the fortification of dairy products can be used to increase their antioxidant content [93] and therefore for the development of functional dairy products. Meanwhile, it seems that the combined use of extracts derived from different herbs can lead to higher bioavailability of bioactive ingredients [94]. This is in accordance with the present study, in which the combination of herbal or byproduct extracts showed higher concentrations of antioxidants and total phenolics before and after in vitro digestion.

To increase the antioxidant capacity and bioavailability of food products such as meat and bakery products, antioxidant compounds have been added to them using encapsulation methods [95,96,97,98]. Nevertheless, it should be considered that the design and development of such products are expensive and time consuming [92]. The present study determined the higher antioxidant content of fortified dairy products compared to that of control samples, while exploiting a simple, cost-effective method for fortification. Specifically, plant byproducts and herbal extracts were utilized to prepare aqueous extracts that were easily incorporated into the final food products. However, an important factor that remains is the final organoleptic characteristics of fortified dairy products, as consumers usually choose taste over the health benefits of functional foods [12].

The pilot organoleptic evaluation implemented in the context of the present study indicated that the overall acceptability of all the newly developed dairy products was average, as most participants stated that they slightly liked the fortified products (scored as an average of three in the five-point scale). Although the dairy products that did not contain any extract presented the highest values for overall acceptability, the evaluation score given by the study participants for organoleptic characteristics between the control and fortified products did not present statistically significant differences, except in the case of the fortified cream cheese products and the yogurt fortified with St. John’s Wort. This may be attributed to the relatively low concentration of the added extract. Specifically, participants positively evaluated the organoleptic characteristics of plain kefir as well as kefir fortified with extracts. In contrast, the participants did not seem to like the flavor of the fortified cream cheese samples compared to plain cream cheese. Participants also identified differences in the color, texture, and flavor of yogurt, but not vegan yogurt, compared with the control samples. Further research is needed on dairy products to obtain favorable organoleptic characteristics. Studies have shown that desirable changes can be achieved through the addition of goat milk and/or different starting bacterial cultures [99,100,101]. This can lead to desirable changes and low-cost solutions for sensory improvement.

Finally, some limitations of the present study should be considered. The in vitro digestion methodology aims to simulate gastrointestinal conditions for the estimation of the bioavailability of different compounds. In the present study, we used an in vitro digestion model to estimate antioxidant and phenolic bioavailability; however, the human digestive system is quite complex and therefore remains challenging to recreate outside the human body [102]. To obtain safer conclusions, further study including clinical interventions is needed. It should also be noted that well-designed organoleptic studies, with training panelists, for the sensory evaluation of novel foods remain crucial. In our study, a pilot organoleptic characterization was implemented with the scope of evaluating the sensory characteristics of the fortified dairy products to obtain information on the potential of developing the studied dairy products and their respective consumer acceptance.

## 5. Conclusions

In conclusion, the fortification of dairy products with aqueous extracts from plant byproducts and herbs can lead to innovative food products with high antioxidant and phenolic content. In the present study, vegan yogurt fortified with lavender or spearmint as well as kefir fortified with a mixture of bitter orange peel and rosehip seed presented high antioxidant and phenolic content compared to plain products, while also being evaluated closely in terms of overall organoleptic characteristics and flavor compared to plain products that can be found in the market. Although these products are promising, organoleptic characteristics can be assumed to be critical for consumer acceptability and therefore for final adaptation from the food industry. Future research on the development of tasteful dairy products and further organoleptic evaluation of consumer preferences for these products is needed.

## Figures and Tables

**Table 1:** Spectrophotometric determination of total antioxidant and phenolic content of selected plant byproduct and herbal extracts and their bioavailability indices.

	Before Digestion		After Digestion					
Sample	Frap (mmol Fe_2_^+^/L)	Folin–Ciocalteu (mg GAE/g)	Frap (mmol Fe_2_^+^/L)	Folin–Ciocalteu (mg GAE/g)	Frap BAvI %	Folin BAvI%	P1	P^2^
Byproduct extracts								
Bitter orange peel	3.89 ± 0.35 ^a^	7.16 ± 2.56 ^a^	1.62 ± 0.11 ^agh^	6.96 ± 3.22 ^abcdef^	42%	55%	>0.05	>0.05
Lemon peel	2.15 ± 0.17 ^a^	4.31 ± 0.54 ^a^	0.83 ± 0.24 ^a^	5.87 ± 3.87 ^abcegh^	36%	68%	<0.05	>0.05
Rosehip seeds	2.85 ± 0.21 ^a^	5.08 ± 0.52 ^a^	0.71 ± 0.15 ^a^	3.24 ± 2.43 ^abcdegh^	23%	62%	>0.05	>0.05
Herbal extracts								
Mountain tea	4.72 ± 0.60 ^a^	10.27 ± 1.37 ^ab^	0.96 ± 0.63 ^abg^	2.48 ± 0.98 ^acdef^	15%	22%	<0.01	>0.05
St. John’s Wort	8.76 ± 1.87 ^b^	23.33 ± 4.93 ^cf^	3.00 ± 0.63 ^cdhij^	3.23 ± 2.55 ^abcdefh^	31%	13%	>0.05	>0.05
Dittany	12.81 ± 1.94 ^b^	23.93 ± 6.33 ^cf^	3.60 ± 1.15 ^chij^	2.32 ± 1.54 ^acdef^	30%	12%	>0.05	<0.05
Lemon balm	46.61 ± 7.22 ^c^	82.97 ± 4.29 ^d^	10.71 ± 1.20 ^e^	9.68 ± 4.31 ^bcgh^	23%	13%	>0.05	>0.05
Spearmint	22.81 ± 1.40 ^d^	39.97 ± 15.36 ^efh^	4.75 ± 1.00 ^fij^	1.33 ± 0.55 ^adef^	21%	4%	>0.05	>0.05
Lavender	9.68 ± 1.19 b^b^	18.30 ± 7.45 ^bcf^	1.98 ± 0.38 ^abdgh^	2.02 ± 0.99 ^abcdef^	19%	7%	<0.01	>0.05
Combination extracts								
St. John’s Wort and Mountain tea	11.80 ± 1.45 ^b^	29.39 ± 5.85 ^cef^	2.70 ± 0.23 ^acdghij^	3.84 ± 0.42 ^abcdefh^	24%	14%	>0.05	>0.05
Bitter orange peel and Rosehip seed	21.87 ± 1.41 ^d^	67.07 ± 1.67 ^g^	3.70 ± 0.50 ^cdfhij^	5.95 ± 2.69 ^abcdegh^	18%	10%	>0.05	>0.05
Bitter orange peel and Lemon peel	12.11 ± 0.41 ^b^	47.92 ± 2.10 ^eh^	3.85 ± 0.35 ^cfhij^	7.42 ± 2.75 ^bcegh^	32%	15%	>0.05	>0.05

Data are presented as mean (*n* = 3) ± SD. Different letters in the same column indicate significant differences (*p* < 0.05) between the samples. BAvI: bioavailability index. P1: correlation of samples before and after in vitro digestion of total antioxidants. P2: correlation of samples before and after in vitro digestion of total phenolics.

**Table 2:** Mean concentrations of total phenolic content and total antioxidant activity of selected herbs and byproducts reported in literature.

Food Sample	Origin of Sample	FRAP Assay (mmol Fe^2+^/g)	Folin–Ciocalteau (mg GAE/g)	Study
**Melissa oficcinalis**	Spain	9.21	382.05	[38]
	Portugal	-	293.32–959.54	[39]
	Romania	-	54.9	[40]
	Greece	2.33	82.97	Present study
**Ditanny**	Greece	-	6.7–21.7	[41]
	Greece	0.64	23.93	Present study
**St John’s Wort**	Turkey	-	104–451.33	[42]
	Greece	0.44	23.33	Present study
**Pennyroyal**	Portugal	0.01	13.3	[43]
	Greece	0.5	27.65	Present study
**Spearmint**	Greece	1.98	29.67	[44]
	Greece	1.14	39.97	Present study
**Levander**	Romania	-	50.6	[40]
	China	-	36.87	[45]
	Greece	0.48	18.30	Present study
**Mountain tea**	Turkey	-	0.507–12.99	[46]
	Greece	0.2	819.03	[44]
	Greece	0.27 × 10^−6^– 4.84 × 10^−6^	23.7 × 10^−6^ −45.6 × 10^−6^	[47]
	Spain	-	102.54	[48]
	Greece	0.24	10.27	Present study
**Bitter orange peel**	Tunisia	-	5.23	[49]
	Iran	27.63–55.13	5.06	[50]
	Greece	0.04	7.16	Present study
**Lemon peel**	Malaysia	4.34	1267.87–1336.77	[51]
	China	-	3.49	[52]
	Portugal	-	222.76	[53]
	Greece	0.02	4.31	Present study
**Wild rose seeds**	Canada	-	481	[54]
	Poland	127	-	[55]
	Italy	-	166.3–212.3	[56]
	Hungary	123.8–314.4	150.8–299.2	[57]
	Greece	0.03	5.08	Present study

**Table 3:** Common phytochemicals identified among the studied extracts and their respective identification frequency.

Phytochemicals	Identification Frequency *
Luteolin 7-O-diglucuronide	11
Salvianolic acid B	9
Rutin	9
Acteoside	8
Nicotiflorin	8
Chrysoeriol 7-O-apiosyl-glucoside	8
Pinoresinol-4-O-Beta-Monoglycoside	7
Naringenin-4′,5-diglucuronide	7
Cirsilineol	7
Hexose	7
Vanillylmandelic acid	7
3,4-Dicaffeoylquinic Acid	6
Luteolin 4′-glucoside	6
Lithospermic acid B	6
Apigenin-7-O-glucoside	6
Caffeoyl tartaric acid	6
Isoscutellarein 7-O-[6‴-O-acetyl-β-d-allopyranosyl-(1→2)]-β-d-glucopyranoside	6
Rosmarinic acid	6
Sucrose	6
Scutellarin	6
Quercetin 3-arabinoside	5
Orientin	5
5-Feruloylquinic acid	5
Ferulic acid-4′-O-glucoside	5
Luteolin	5
Hesperidin	5
Leucosceptoside A	5
Chicoric acid	5
Diosmin	5
Oleuropein	5
Astilbin	5
Luteolin-3-O-glucuronide	5
Kaempferol 3-O-sophoroside	5
Isorhamnetin 3-O-galactoside	5

* Identification frequency refers to the frequency that the specific phytochemical was detected among the tested samples on the 18 different recordings (ESI + and ESI−).

**Table 4:** Most common antioxidants identified in Lamicaea family of studied plant materials.

Phytochemicals	Identification Frequency *
Luteolin 7-O-diglucuronide	9
Acteoside	8
Vanillylmandelic acid	7
Scutellarin	6
Salvianolic acid B	6
Lithospermic acid B	6
Rosmarinic acid	5
3,4-Dicaffeoylquinic Acid	5
Luteolin	5
Chicoric acid	4
5-Feruloylquinic acid	4
Apigenin-7-O-glucoside	4
Nicotiflorin	4
Pinoresinol-4-O-Beta-Monoglycoside	4
Leucosceptoside A	4
Pectolinarigenin	3
Silybin	3
Rutin	3
Hesperidin	3
Oleuropein	3
1,3-Dicaffeoylquinic acid	3
Diosmin	3
kaempferol 3-O-rutinoside	3
Salvianolic acid C	3
Cirsilineol	3
Theaflavin 3-O-gallate	3
Luteolin 7-O-glucoside	3
Cynarin	3

* Identification Frequency refers to the frequency that the specific phytochemical was detected among the tested samples on the 18 different recordings (ESI+ and ESI−).

**Table 5:** Most common, by count, antioxidants identified in the Rutaceae family of studied plant materials.

Phytochemicals	Identification Frequency *
Rutin	4
Limonin	4
Eriocitrin_1	4
Isorhamnetin-3-O-rutinoside	3
Nicotiflorin	3
Orientin	3
Kaempferol 3-O-sophoroside	2
Isorhamnetin 3-O-galactoside	2
Citric acid	2
Didymin	2
Cirsimaritin	2
Pinoresinol-4-O-Beta-Monoglycoside	2
Astilbin	2
Nobiletin	2
D-(+)-Mannose	2
Azadirachtin	2
Quercetin-3-O-glucoside	2
Allobetonicoside	2
Rhoifolin	2
Eupatilin	2
1,2-Disinapoylgentiobiose	2
Hesperidin	2

* Identification Frequency refers to the frequency that the specific phytochemical was detected among the tested samples on the 18 different recordings (ESI+ and ESI−).

**Table 6:** Total antioxidant and phenolic content in fortified food products after in vitro digestion.

Sample	Concentration of Extract (mL/100 g of Diary Product)	FRAP (mmol Fe^2+^/L)	Folin–Ciocalteu (mg GAE/g)
**Kefir**			
Control *	0	0.09 ± 0.10 ^a^	0.39 ± 0.71 ^a^
Bitter orange peel	27	0.53 ± 0.23 ^a^	1.92 ± 0.59 ^a^
Bitter orange and lemon peels	10 and 17, respectively	0.57 ± 0.06 ^b^	1.22 ± 0.48 ^a^
Bitter orange and rosehip seed	10 and 17, respectively	0.68 ± 0.06 ^b^	1.39 ± 0.44 ^a^
**Cream cheese**			
Control *	0	0.24 ± 0.10 ^a^	2.02 ± 0.39 ^a^
Mountain tea	35	0.34 ± 0.11 ^ab^	2.28 ± 0.65 ^abc^
St. John’s Wort	35	0.53 ± 0.16 ^c^	2.82 ± 0.36 ^bc^
Mountain tea and St. John’s Wort	25 and 10, respectively	0.44 ± 0.13 ^bc^	2.07 ± 0.75 ^a^
**Yogurt**			
Control *	0	0.18 ± 0.06 ^a^	0.91 ± 0.37 ^a^
Dittany	34	0.41 ± 0.04 ^bc^	0.92 ± 0.53 ^a^
St. John’s Wort	50	0.40 ± 0.05 ^bcd^	1.04 ± 0.46 ^a^
Lemon balm	32	1.21 ± 0.12 ^e^	0.92 ± 0.64 ^a^
**Vegan yogurt**			
Control *	0	0.09 ± 0.02 ^a^	1.57 ± 0.44 ^a^
Spearmint	20	0.32 ± 0.04 ^b^	2.00 ± 0.41 ^b^
Lavender	20	0.20 ± 0.04 ^c^	2.01 ± 0.40 ^b^

* Food products without extracts were used as control samples. The data are expressed as mean (*n* = 3) ± SD. Different letters in the same column for each food product category indicate significant differences (*p <* 0.05) between the samples.

**Table 7:** Evaluation of color, aroma, texture and flavor of the fortified new dairy products.

Product	Extract	Color	Aroma	Texture	Flavor	Total Acceptability
Kefir	Control	3.8 ± 1.2 ^a^	3.4 ± 0.8 ^a^	3.4 ± 1.0 ^a^	3.0 ± 1.3 ^a^	3.2 ± 1.1 ^a^
	Bitter orange peel	3.8 ± 1.0 ^a^	3.3 ± 0.8 ^a^	3.5 ± 1.2 ^a^	3.0 ± 1.2 ^a^	2.9 ± 1.1 ^a^
	Bitter orange and Lemon peels	3.6 ± 1.0 ^a^	3.4 ± 0.9 ^a^	3.4 ± 1.2 ^a^	2.3 ± 1.5 ^a^	2.6 ± 1.2 ^a^
	Bitter orange peel and Rosehip seed	3.6 ± 1.2 ^a^	3.5 ± 0.9 ^a^	3.6 ± 1.0 ^a^	3.3 ± 1.1 ^a^	3.2 ± 0.9 ^a^
Cream cheese	Control	3.9 ± 1.2 ^a^	3.8 ± 1.2 ^a^	4.1 ± 1.2 ^a^	4.3 ± 1.0 ^a^	4.0 ± 1.1 ^a^
	St. John’s Wort	3.1 ± 1.1 ^a^	3.0 ± 1.1 ^a^	3.4 ± 1,0 ^a^	3.2 ± 1.2 ^b^	3.2 ± 0.9 ^b^
	Mountain tea	3.6 ± 1.0 ^a^	3.1 ± 1.2 ^a^	3.2 ± 1.2 ^b^	2.3 ± 1.3 ^c^	2.6 ± 1.0 ^b^
	St. John’s Wort and Mountain tea	3.7 ± 1.1 ^a^	3.1 ± 1.0 ^a^	2.9 ± 1.2 ^b^	2.9 ± 1.1 ^bc^	3.1 ± 1.1 ^b^
Yogurt	Control	4.1 ± 0.8 ^a^	3.9 ± 0.9 ^a^	4.3 ± 0.8 ^a^	3.9 ± 0.8 ^a^	3.9 ± 0.8 ^a^
	Dittany	3.8 ± 0.9 ^ab^	3.6 ± 1.0 ^a^	3.2 ± 1.1 ^bc^	2.9 ± 1.2 ^b^	3.2 ± 0.9 ^ab^
	St. John’s Wort	3.9 ± 1.1 ^b^	3.1 ± 0.9 ^a^	2.8 ± 0.8 ^c^	2.6 ± 0.9 ^b^	2.8 ± 0.8 ^b^
	Lemon balm	3.6 ± 0.8 ^ab^	3.3 ± 1.0 ^a^	3.8 ± 0.7 ^ab^	3.1 ± 1.3 ^ab^	3.2 ± 1.1 ^ab^
Vegan yogurt	Control	3.4 ± 1.1 ^a^	3.4 ± 1.0 ^a^	3.5 ± 1.1 ^a^	2.8 ± 1.2 ^a^	2.8 ± 1.2 ^a^
	Lavender	3.3 ± 1.1 ^a^	3.1 ± 1.1 ^a^	2.7 ± 1.0 ^ab^	2.4 ± 1.0 ^a^	2.4 ± 1.1 ^a^
	Spearmint	2.9 ± 1.0 ^a^	3.1 ± 0.9 ^a^	2.7 ± 0.9 ^b^	2.5 ± 1.1 ^a^	2.6 ± 1.0 ^a^

Data are presented as mean (*n* = 3) ± SD. Different letters in the same column for each food product category indicate significant differences (*p* < 0.05) between the samples.

## Data Availability

The data presented in this study are available in the present article and the Supplementary Material.

## Supplementary Materials

The following supporting information can be downloaded at: https://www.mdpi.com/article/10.3390/antiox12020500/s1. Table S1: The 162 different identified phytochemicals in the 9 studied plant materials. The compounds in blue color belong to the family of polyphenols; Table S2: Identified compounds in bitter orange aqueous extract (ESI negative mode); Table S3: Identified compounds in bitter orange aqueous extract (ESI positive mode); Table S4: Identified compounds in dittany aqueous extract (ESI negative mode); Table S5: Identified compounds in dittany aqueous extract (ESI positive mode); Table S6: Identified compounds in lavender aqueous extract (ESI negative mode); Table S7: Identified compounds in lavender aqueous extract (ESI positive mode); Table S8: Identified compounds in lemon balm aqueous extract (ESI negative mode); Table S9: Identified compounds in lemon balm aqueous extract (ESI positive mode); Table S10: Identified compounds in lemon peel aqueous extract (ESI negative mode); Table S11: Identified compounds in lemon peel aqueous extract (ESI positive mode); Table S12: Identified compounds in rosehip aqueous extract (ESI negative mode); Table S13: Identified compounds in rosehip aqueous extract (ESI positive mode); Table S14: Identified compounds in sideritis aqueous extract (ESI negative mode); Table S15: Identified compounds in sideritis aqueous extract (ESI positive mode); Table S16: Identified compounds in spearmint aqueous extract (ESI negative mode); Table S17: Identified compounds in spearmint aqueous extract (ESI positive mode); Table S18: Identified compounds in St. John’s wort aqueous extract (ESI negative mode); Table S19: Identified compounds in St. John’s wort aqueous extract (ESI positive mode); Table S20: Total phytochemical profile of the 9 studied plants; Table S21: Phytochemicals of bitter orange with antioxidant activity according to international literature; Table S22: Phytochemicals of dittany with antioxidant activity according to international literature; Table S23: Phytochemicals of lemon peel with antioxidant activity according to international literature.; Table S24: Phytochemicals of spearmint with antioxidant activity according to international literature; Table S25: Phytochemicals of lavender with antioxidant activity according to international literature; Table S26: Phytochemicals of lemon balm with antioxidant activity according to international literature; Table S27: Phytochemicals of sideritis with antioxidant activity according to international literature; Table S28: Phytochemicals of St. John’s wort with antioxidant activity according to international literature; Table S29: Phytochemicals of rosehip with antioxidant activity according to international literature; Table S30: Polyphenolic components of each studied plant; Table S31: Chromatographic Conditions including elution program and mobile phases. Phase A was water (H2O) with 0.1% formic acid (CH3COOH) and phase B was acetonitrile (CH3CN) with 0.1% formic acid (CH3COOH); Figure S1: St. John’s wort’s total ion chromatogram (negative ionization mode); Figure S2: St. John’s wort’s total ion chromatogram (positive ionization mode); Figure S3: Lemon balm’s total ion chromatogram (negative ionization mode); Figure S4: Lemon balm’s total ion chromatogram (positive ionization mode); Figure S5: Dittany’s total ion chromatogram (negative ionization mode); Figure S5: Dittany’s total ion chromatogram (negative ionization mode); Figure S7: Rosehip’s total ion chromatogram (negative ionization mode); Figure S8: Rosehip’s total ion chromatogram (positive ionization mode; Figure S9: Bitter orange’s total ion chromatogram (negative ionization mode); Figure S10: Bitter orange’s total ion chromatogram (positive ionization mode); Figure S11: Spearmint’s total ion chromatogram (negative ionization mode); Figure S12: Spearmint’s total ion chromatogram (positive ionization mode); Figure S13: Sideritis’s total ion chromatogram (negative ionization mode); Figure S14: Sideritis’s total ion chromatogram (positive ionization mode); Figure S15: Lemon peel’s total ion chromatogram (negative ionization mode); Figure S16: Lemon peel’s total ion chromatogram (positive ionization mode); Figure S17: Lavender’s total ion chromatogram (negative ionization mode); Figure S18: Lemon peel’s total ion chromatogram (positive ionization mode).
