# Supplementary material for: Development of Dairy Products Fortified with Plant Extracts: Antioxidant and Phenolic Content Characterization
Source: Antioxidants (Basel). 2023 Feb 16;12(2):500. doi: 10.3390/antiox12020500 (PMC9952465; doi:10.3390/antiox12020500)
Supplement: Supplementary file 1 [file antioxidants-12-00500-s001.zip › antioxidants-2211400-supplementary.pdf]

Table S1. The 162 different identified phytochemicals in the 9 studied plant materials. The compounds in blue color belong to the family of polyphenols.

Sr. No	Phytochemical
1	Rhoifolin
2	Eriocitrin
3	Apigenin-7-O-glucoside
4	Limonin
5	Didymin
6	Isorhamnetin-3-O-rutinoside
7	Isorhamnetin 3-O-galactoside
8	Citric acid
9	Eupatilin
10	Luteolin 7-O-diglucuronide
11	Quercetin-3-O-glucoside
12	Kaempferol 3-O-sophoroside
13	Myricetin 3-alpha-L-arabinopyranoside
14	Manghaslin (Quercetin 3-2G-rhamnosylrutinoside)
15	Orientin
16	Rutin
17	Glucoalyssin
18	Kaempferol 3-O-acetyl-glucoside
19	1,2-Disinapoylgentiobiose
20	Spinacetin 3-O-glucosyl-(1->6)-glucoside
21	Glucogallin
22	Cirsilineol
23	D-(+)-Mannose
24	Azadirachtin

25	Patulitrin
26	Bergenin
27	Allobetonicoside
28	Nicotiflorin
29	5-Feruloylquinic acid
30	Neodiosmin
31	Ferulic acid-4'-O-glucoside
32	Feruloyl C1-glucuronide
33	3,4-Dicaffeoylquinic Acid
34	Isoscutellarein 7-O-[6‴-O-acetyl-β-d-allopyranosyl-(1→2)]-β-d-glucopyranoside
35	Salvianolic acid G
36	9-F1-phytoprostane
37	Barbatoside C/D
38	Quercetin 3-arabinoside
39	Quercetin 3-O-(6''-acetyl-galactoside) 7-O-rhamnoside
40	5,5'-Dicaffeic acid
41	Isoscutellarein 4'-methyl ether 7-(6'''-acetylallosyl)(1->2)-glucoside
42	6''-O-Malonyldaidzin
43	Caffeoyl tartaric acid
44	Naringenin-4',5-diglucuronide
45	Luteolin 4'-glucoside
46	Apigenin 7-O-apiosyl-glucoside
47	Kaempferol 3-O-(6''-acetyl-galactoside) 7-O-rhamnoside
48	Tragopogonic acid
49	Nobiletin
50	Peonidin 3-O-sophoroside

51	Betonicine
52	Sucrose
53	Pelargonidin 3-O-rutinoside
54	Luteolin 7-O-rutinoside
55	Hexose
56	Chrysoeriol 7-O-apiosyl-glucoside
57	Cirsimaritin
58	5,7-Dihydroxychromone
59	Morroniside
60	Echinacoside
61	6''-O-Acetylgenistin
62	Leucosceptoside A
63	Quercetin 3-rutinoside-7-glucoside
64	5-(3'-hydroxyphenyl)-gamma-hydroxyvaleric acid -4'-O-glucuronide
65	Salvianolic acid B
66	Rosmarinic acid
67	Genistein 4',7-O-diglucuronide
68	Lithospermic acid
69	Lithospermic acid B
70	Scutellarin
71	Salvianolic acid C
72	Pectolinarigenin
73	Eupatorin
74	Vanillylmandelic acid
75	Cynarin
76	Theaflavin 3-O-gallate

77	Isorhamnetin 3-O-glucoside
78	Juglanin
79	Cafestol (2-hydroxy-)
80	Astilbin
81	Pinoresinol-4-O-Beta-Monoglycoside
82	4-Hydroxy-5-(3',5'-dihydroxyphenyl)-valeric acid-O-glucuronide
83	6''-O-Malonylgenistin
84	Geniposidic-Acid
85	Isoacteoside
86	Homoplantaginin_Tectoridin
87	Kaempferol 3-rhamnosyl-(1->2)-rhamnosyl-(1->6)-glucoside
88	Harpagide
89	Naringenin 7-O-glucoside
90	Diosmin
91	Chicoric acid
92	Aucubin
93	Apigenin 7-O-diglucuronide
94	Hispidulin glucuronide
95	Acteoside
96	Silybin
97	Plumieride
98	Hydroxytyrosol 4-O-glucoside
99	beta-D-Glcp-(1->4)-[L-alpha-D-Hepp-(1->3)]-L-alpha-D-Hepp
100	Luteolin
101	alpha-Methyl-D-mannopyranoside
102	Dihydroferulic acid 4-O-glucuronide

103	Apigenin
104	Quercitrin (Quercetin-3-O-alpha-L-rhamnopyranoside)
105	Luteolin-3-O-glucuronide
106	Quercetin 3'-O-glucuronide
107	Melittoside
108	Oleuropein
109	Astragalin
110	5-O-Caffeoylquinic acid
111	Genkwanin
112	Caffeic acid 4-O-glucoside
113	Salvianolic Acid A
114	1,3-Dicaffeoylquinic acid
115	Quercetin 3-O-beta-D-glucopyranosyl-7-O-alpha-L-rhamnopyranoside
116	Diosmetin
117	Dihydroferulic acid-4'-O-glucuronide
118	Luteolin 7-O-glucoside
119	Silydianin
120	Achillolide A
121	Glucobrassicanapin
122	Hesperidin
123	Isorhamnetin 3-O-glucoside 7-O-rhamnoside
124	Chrysoeriol
125	Hesperetin
126	Barbatoside A/B
127	kaempferol 3-O-rutinoside
128	Antoside

129	Isorhamnetin 4'-O-glucuronide
130	Byakangelicin
131	Myricetin-3-O-α-L-rhamnopyranoside
132	Coumaroyl tartaric acid (p-)
133	Quercetin 3-O-(6-malonyl-glucoside)''
134	4-O-Caffeoylquinic acid
135	Valoneic acid dilactone
136	5-O-Galloylquinic acid
137	Protocatechuic acid 4-O-glucoside
138	Taxifolin
139	Ascorbic acid (L-)
140	Teupolioside
141	Apigenin 7-(4''-E-p-coumarylglucoside)
142	Isoscutellarein 7-O-[6'-O-acetyl-β-D-allopyranosyl-(1→2)]-6''-O-acetyl-β-D-glucopyranoside''
143	Rhamnetin 3-glucoside
144	Falcarindiol 3-acetate
145	Apigenin 7-(2'',3''-diacetyl-hexocide)
146	Apigenin-7-O-glucuronide
147	5-O-Caffeoylshikimic acid
148	Quercetin
149	Cinnamtannin A2
150	Quercetin 3,4'-O-diglucoside
151	Anhydrochiisanogenoic acid
152	Amentoflavone
153	Kaempferol
154	Myricetin 7-glucoside

155	Coumaroylquinic acid
156	Quercetin 3-glucuronate
157	Hyperoside
158	Naringenin 5-O-glucuronide
159	Kaempferol-3-o-glucuronide
160	Myricetin-3-O-galactopyranoside
161	Caffeic acid
162	Phloridzin

Table S2. Identified compounds in bitter orange aqueous extract (ESI negative mode).

Component name	Neutral mass (Da)	Observed m/z	Mass error (ppm)	Mass error (mDa)	Formula
1,2-Disinapoylgentiobiose	754,23203	753,2249	0,2	0,1	$C_{34}H_{42}O_{19}$
Isoscutellarein 7-O-[6‴-O-acetyl-β-d-allopyranosyl-(1→2)]-β-d-glucopyranoside	652,16395	651,1574	1,2	0,8	$C_{29}H_{32}O_{17}$
Rhoifolin	578,16356	577,1565	0,3	0,2	$C_{27}H_{30}O_{14}$
Eriocitrin_1	596,17412	595,1673	0,8	0,4	$C_{27}H_{32}O_{15}$
Orientin	448,10056	447,0935	0,5	0,2	$C_{21}H_{20}O_{11}$
Salvianolic acid G	418,09	417,0831	0,8	0,3	$C_{20}H_{18}O_{10}$
Kaempferol 3-O-acetyl-glucoside	490,11113	489,1039	0,1	0,1	$C_{23}H_{22}O_{12}$
D-(+)-Mannose	180,06339	179,0563	0,9	0,2	$C_6H_{12}O_6$
Nicotiflorin	594,15847	593,152	1,4	0,8	$C_{27}H_{30}O_{15}$
Didymin	594,19486	593,1873	-0,4	-0,3	$C_{28}H_{34}O_{14}$
5-Feruloylquinic acid	368,11073	367,1028	-1,9	-0,7	$C_{17}H_{20}O_9$
Rutin	610,15338	609,1474	2,1	1,3	$C_{27}H_{30}O_{16}$
3,4-Dicaffeoylquinic Acid	516,12678	515,1199	0,7	0,4	$C_{25}H_{24}O_{12}$
Azadirachtin	720,26294	719,2538	-2,6	-1,9	$C_{35}H_{44}O_{16}$

Ferulic acid-4'-O-glucoside	356,11073	355,1031	-1,1	-0,4	$C_{16}H_{2}0O_{9}$
Eupatilin	344,0896	343,0821	-0,8	-0,3	$C_{18}H_{16}O_{7}$
Kaempferol 3-O-sophoroside	610,15338	609,1465	0,6	0,4	$C_{27}H_{3}0O_{16}$
9-F1-phytoprostane	328,22497	327,2174	-1	-0,3	$C_{18}H_{32}O_{5}$
Barbatoside C/D	810,25824	809,2483	-3,3	-2,7	$C_{37}H_{46}O_{2}0$
Limonin	470,19407	469,1864	-0,7	-0,4	$C_{26}H_{3}0O_{8}$
Isorhamnetin-3-O-rutinoside	624,16903	623,1626	1,3	0,8	$C_{28}H_{32}O_{16}$
Quercetin 3-arabinoside	434,08491	433,0784	1,8	0,8	$C_{2}0H_{18}O_{11}$
Bergenin	328,07943	327,0717	-1,3	-0,4	$C_{14}H_{16}O_{9}$
Cirsilineol_1	344,0896	343,0822	-0,4	-0,1	$C_{18}H_{16}O_{7}$
Eriocitrin_2	596,17412	595,1657	-1,8	-1,1	$C_{27}H_{32}O_{15}$
Spinacetin 3-O-glucosyl-(1->6)-glucoside	670,17451	669,1682	1,5	1	$C_{29}H_{34}O_{18}$
Quercetin 3-O-(6''-acetyl-galactoside) 7-O-rhamnoside	652,16395	651,155	-2,6	-1,7	$C_{29}H_{32}O_{17}$
Isorhamnetin 3-O-galactoside	478,11113	477,1042	0,8	0,4	$C_{22}H_{22}O_{12}$
5,5'-Dicaffeic acid	358,06887	357,062	1,1	0,4	$C_{18}H_{14}O_{8}$
Manghaslin (Quercetin 3-2G-rhamnosylrutinoside)	756,21129	755,2044	0,6	0,4	$C_{33}H_{4}0O_{2}0$
Isoscutellarein 4'-methyl ether 7-(6'''-	666,1796	665,1718	-0,7	-0,5	$C_{3}0H_{34}O_{17}$

acetylallosyl)(1->2)-glucoside					
Quercetin-3-O-glucoside	464,09548	463,0886	0,8	0,4	$C_{21}H_{20}O_{12}$
Allobetonicoside	506,16356	505,1558	-1	-0,5	$C_{21}H_{30}O_{14}$
Feruloyl C1-glucuronide	370,09	369,0826	-0,3	-0,1	$C_{16}H_{18}O_{10}$
Neodiosmin	608,17412	607,166	-1,3	-0,8	$C_{28}H_{32}O_{15}$
Glucogallin	332,07435	331,0681	3,1	1	$C_{13}H_{16}O_{10}$
Apigenin-7-O-glucoside	432,10565	431,0984	0,2	0,1	$C_{21}H_{20}O_{10}$
6''-O-Malonyldaidzin	502,11113	501,104	0,3	0,1	$C_{24}H_{22}O_{12}$
Caffeoyl tartaric acid	312,04813	311,0406	-0,8	-0,2	$C_{13}H_{12}O_{9}$
Naringenin-4',5-diglucuronide	608,13773	607,13	-0,8	-0,5	$C_{27}H_{28}O_{16}$
Luteolin 4'-glucoside	448,10056	447,0927	-1,2	-0,5	$C_{21}H_{20}O_{11}$
Apigenin 7-O-apiosyl-glucoside	564,14791	563,1417	1,9	1	$C_{26}H_{28}O_{14}$
Patulitrin	494,10604	493,0976	-2,4	-1,2	$C_{22}H_{22}O_{13}$
Kaempferol 3-O-(6''-acetyl-galactoside) 7-O-rhamnoside	636,16903	635,1591	-4,2	-2,7	$C_{29}H_{32}O_{16}$
Luteolin 7-O-diglucuronide_1	638,11191	637,1023	-3,6	-2,3	$C_{27}H_{26}O_{18}$
Glucoalyssin	451,06406	450,0586	4	1,8	$C_{13}H_{25}NO_{10}S_{3}$
Tragopogonic acid	288,06339	287,0558	-1,1	-0,3	$C_{15}H_{12}O_{6}$
Citric acid	192,027	191,0194	-1,7	-0,3	$C_{6}H_{8}O_{7}$
Myricetin 3-alpha-L-arabinopyranoside	450,07983	449,0735	2	0,9	$C_{20}H_{18}O_{12}$

Table S3. Identified compounds in bitter orange aqueous extract (ESI positive mode).

Component name	Neutral mass (Da)	Observed m/z	Mass error (ppm)	Mass error (mDa)	Formula
Rhoifolin	578,16356	579,1708	0	0	$C_{27}H_{30}O_{14}$
Nobiletin	402,13147	425,1198	-2	-0,9	$C_{21}H_{22}O_{8}$
Peonidin 3-O-sophoroside	625,17686	625,1764	0,1	0,1	$C_{28}H_{33}O_{16}$
Betonicine	159,08954	160,0965	-2,1	-0,3	$C_{7}H_{13}NO_{3}$
1,2-Disinapoylgentiobiose	754,23203	777,2219	0,8	0,6	$C_{34}H_{42}O_{19}$
Tragopogonic acid	288,06339	289,07	-2,4	-0,7	$C_{15}H_{12}O_{6}$
Isoscutellarein 7-O-[6′′′-O-acetyl-β-d-allopyranosyl-(1→2)]-β-d-glucopyranoside	652,16395	653,1725	2	1,3	$C_{29}H_{32}O_{17}$
Orientin	448,10056	449,1077	-0,4	-0,2	$C_{21}H_{20}O_{11}$
Sucrose	342,11621	365,1054	0	0	$C_{12}H_{22}O_{11}$
Pelargonidin 3-O-rutinoside	579,17138	579,171	0,3	0,2	$C_{27}H_{31}O_{14}$
Luteolin 7-O-rutinoside	594,15847	595,1657	-0,1	-0,1	$C_{27}H_{30}O_{15}$
Hexose	180,06339	203,0525	-0,5	-0,1	$C_{6}H_{12}O_{6}$
Limonin	470,19407	471,2007	-1,3	-0,6	$C_{26}H_{30}O_{8}$

Chrysoeriol 7-O-apiosyl-glucoside	594,15847	595,1659	0,3	0,2	$C_{27}H_{30}O_{15}$
9-F1-phytoprostane	328,22497	351,213	-3,5	-1,2	$C_{18}H_{32}O_{5}$
Eupatilin	344,0896	345,0961	-2,2	-0,8	$C_{18}H_{16}O_{7}$
Kaempferol 3-O-sophoroside	610,15338	611,1615	1,3	0,8	$C_{27}H_{30}O_{16}$
Eriocitrin_1	596,17412	597,182	1,1	0,6	$C_{27}H_{32}O_{15}$
Cirsimaritin	314,07904	315,0859	-1,3	-0,4	$C_{17}H_{14}O_{6}$
Didymin	594,19486	617,1826	-2,4	-1,5	$C_{28}H_{34}O_{14}$
5,7-Dihydroxychromone	178,02661	179,0336	-1,4	-0,2	$C_{9}H_{6}O_{4}$
Morroniside	406,14751	429,1362	-1,4	-0,6	$C_{17}H_{26}O_{11}$
Echinacoside	786,25824	787,2643	-1,5	-1,2	$C_{35}H_{46}O_{20}$
6''-O-Acetylgenistin	474,11621	475,1216	-4	-1,9	$C_{23}H_{22}O_{11}$
Leucosceptoside A	638,22107	661,2118	2,2	1,5	$C_{30}H_{38}O_{15}$
Rutin	610,15338	633,1437	1,7	1,1	$C_{27}H_{30}O_{16}$
Quercetin 3-rutinoside-7-glucoside	772,20621	773,2131	-0,6	-0,4	$C_{33}H_{40}O_{21}$
Quercetin 3-arabinoside	434,08491	457,0736	-1,3	-0,6	$C_{20}H_{18}O_{11}$
5-(3'-hydroxyphenyl)-gamma-hydroxyvaleric acid -4'-O-glucuronide	402,11621	403,1223	-3	-1,2	$C_{17}H_{22}O_{11}$

Table S4. Identified compounds in dittany aqueous extract (ESI negative mode).

Component name	Neutral mass (Da)	Observed m/z	Mass error (ppm)	Mass error (mDa)	Formula
Salvianolic acid B_1	718,15338	717,1451	-1,5	-1,1	$C_{36}H_{30}O_{16}$
Luteolin 7-O-diglucuronide_1	638,11191	637,1052	0,9	0,6	$C_{27}H_{26}O_{18}$
Rosmarinic acid	360,08452	359,0778	1,6	0,6	$C_{18}H_{16}O_{8}$
Genistein 4',7-O-diglucuronide	622,117	621,1101	0,6	0,3	$C_{27}H_{26}O_{17}$
Lithospermic acid_1	538,11113	537,1045	1,2	0,7	$C_{27}H_{22}O_{12}$
ferulic acid-4'-O-glucoside	718,15338	717,1465	0,5	0,4	$C_{36}H_{30}O_{16}$
Cirsilineol_1	344,0896	343,0824	0,3	0,1	$C_{18}H_{16}O_{7}$
9-F1-phytoprostane	328,22497	327,2177	0	0	$C_{18}H_{32}O_{5}$
Scutellarin	462,07983	461,0733	1,6	0,7	$C_{21}H_{18}O_{12}$
Salvianolic acid C	492,10565	491,0984	0,1	0	$C_{26}H_{20}O_{10}$
Pectolinarigenin	314,07904	313,072	0,8	0,2	$C_{17}H_{14}O_{6}$
Orientin	448,10056	447,0937	0,8	0,4	$C_{21}H_{20}O_{11}$
Eupatorin	344,0896	343,0828	1,4	0,5	$C_{18}H_{16}O_{7}$
Vanillylmandelic acid	198,05282	197,0456	0,5	0,1	$C_{9}H_{10}O_{5}$
Cynarin	516,12678	515,119	-0,9	-0,5	$C_{25}H_{24}O_{12}$
Rutin	610,15338	609,1463	0,3	0,2	$C_{27}H_{30}O_{16}$
Theaflavin 3-O-gallate_1	716,13773	715,131	0,7	0,5	$C_{36}H_{28}O_{16}$
Isorhamnetin 3-O-glucoside	478,11113	477,1046	1,6	0,8	$C_{22}H_{22}O_{12}$
Juglanin	418,09	417,0831	0,8	0,3	$C_{20}H_{18}O_{10}$
3,4-Dicaffeoylquinic Acid	516,12678	515,1182	-2,5	-1,3	$C_{25}H_{24}O_{12}$

Cafestol (2-hydroxy-)	332,19876	331,1908	-2	-0,6	$C_{20}H_{28}O_4$
Chrysoeriol 7-O-apiosyl-glucoside	594,15847	593,1514	0,4	0,3	$C_{27}H_{30}O_{15}$
Isorhamnetin 3-O-galactoside	478,11113	477,1049	2,2	1	$C_{22}H_{22}O_{12}$
Ferulic acid-4'-O-glucoside	356,11073	355,1037	0,7	0,2	$C_{16}H_{20}O_9$
Astilbin	450,11621	449,1095	1,3	0,6	$C_{21}H_{22}O_{11}$
Kaempferol 3-O-sophoroside	610,15338	609,1459	-0,3	-0,2	$C_{27}H_{30}O_{16}$
Pinoresinol-4-O-Beta-Monoglycoside	520,19446	519,1894	4,2	2,2	$C_{26}H_{32}O_{11}$
4-Hydroxy-5-(3',5'-dihydroxyphenyl)-valeric acid-O-glucuronide	402,11621	401,1091	0,5	0,2	$C_{17}H_{22}O_{11}$
Glucogallin	332,07435	331,0672	0,4	0,1	$C_{13}H_{16}O_{10}$
Naringenin-4',5-diglucuronide	608,13773	607,129	-2,4	-1,5	$C_{27}H_{28}O_{16}$
6''-O-Malonylgenistin	518,10604	517,0985	-0,5	-0,2	$C_{24}H_{22}O_{13}$
Geniposidic-Acid	374,1213	373,1139	-0,3	-0,1	$C_{16}H_{22}O_{10}$
Isoacteoside	624,20542	623,1954	-4,4	-2,7	$C_{29}H_{36}O_{15}$
Homoplantaginin_Tectoridin	462,11621	461,1091	0,4	0,2	$C_{22}H_{22}O_{11}$
Kaempferol 3-rhamnosyl-(1->2)-rhamnosyl-(1->6)-glucoside	740,21638	739,2074	-2,3	-1,7	$C_{33}H_{40}O_{19}$
Harpagide	364,13695	363,1281	-4,3	-1,6	$C_{15}H_{24}O_{10}$
Naringenin 7-O-glucoside	434,1213	433,112	-4,7	-2,1	$C_{21}H_{22}O_{10}$
Diosmin	608,17412	607,1664	-0,7	-0,4	$C_{28}H_{32}O_{15}$
Chicoric acid	474,07983	473,0715	-2,2	-1	$C_{22}H_{18}O_{12}$
Aucubin	346,12638	345,1188	-0,8	-0,3	$C_{15}H_{22}O_9$

Table S5. Identified compounds in dittany aqueous extract (ESI positive mode).

Component name	Neutral mass (Da)	Observed m/z	Mass error (ppm)	Mass error (mDa)	Formula
Luteolin 7-O-diglucuronide_1	638,11191	639,1202	1,5	1	$C_{27}H_{26}O_{18}$
Genistein 4',7-O-diglucuronide	622,117	623,1253	1,7	1	$C_{27}H_{26}O_{17}$
Salvianolic acid B_1	718,15338	741,1427	0,1	0,1	$C_{36}H_{30}O_{16}$
Scutellarin	462,07983	463,0874	0,7	0,3	$C_{21}H_{18}O_{12}$
Cirsilineol_1	344,0896	345,0965	-1,1	-0,4	$C_{18}H_{16}O_{7}$
9-F1-phytoprostane	328,22497	351,2134	-2,4	-0,8	$C_{18}H_{32}O_{5}$
Lithospermic acid	538,11113	561,1011	1,4	0,8	$C_{27}H_{22}O_{12}$
Sucrose	342,11621	365,1052	-0,6	-0,2	$C_{12}H_{22}O_{11}$
Eupatorin	344,0896	345,0963	-1,8	-0,6	$C_{18}H_{16}O_{7}$
Pectolinarigenin	314,07904	315,0861	-0,6	-0,2	$C_{17}H_{14}O_{6}$
Orientin	448,10056	449,1082	0,7	0,3	$C_{21}H_{20}O_{11}$
Hexose	180,06339	203,0524	-1,1	-0,2	$C_{6}H_{12}O_{6}$
Lithospermic acid B	718,15338	741,144	1,9	1,4	$C_{36}H_{30}O_{16}$
Apigenin 7-O-diglucuronide	622,117	645,1072	1,6	1	$C_{27}H_{26}O_{17}$
Hispidulin glucuronide	476,09548	477,1028	0,1	0	$C_{22}H_{20}O_{12}$

Chrysoeriol 7-O-apiosyl-glucoside	594,15847	595,1666	1,5	0,9	$C_{27}H_{30}O_{15}$
Rosmarinic acid	360,08452	383,0734	-0,8	-0,3	$C_{18}H_{16}O_{8}$
Acteoside	624,20542	647,1954	1,2	0,8	$C_{29}H_{36}O_{15}$
3,4-Dicaffeoylquinic Acid	516,12678	539,1176	3	1,6	$C_{25}H_{24}O_{12}$
Salvianolic acid C	492,10565	515,0944	-0,9	-0,4	$C_{26}H_{20}O_{10}$
Silybin	482,1213	505,1118	2,5	1,3	$C_{25}H_{22}O_{10}$
Plumieride	470,14243	471,1478	-3,9	-1,9	$C_{21}H_{26}O_{12}$
Hydroxytyrosol 4-O-glucoside	316,11582	339,1046	-1,3	-0,4	$C_{14}H_{20}O_{8}$
beta-D-Glcp-(1->4)-[L-alpha-D-Hepp-(1->3)]-L-alpha-D-Hepp	564,19016	587,1767	-4,5	-2,6	$C_{20}H_{36}O_{18}$
5-Feruloylquinic acid	368,11073	391,101	2,8	1,1	$C_{17}H_{20}O_{9}$
Vanillylmandelic acid	198,05282	221,043	4,4	1	$C_{9}H_{10}O_{5}$

Table S6. Identified compounds in lavender aqueous extract (ESI negative mode).

Component name	Neutral mass (Da)	Observed m/z	Mass error (ppm)	Mass error (mDa)	Formula
Luteolin 7-O-diglucuronide_2	638,11191	637,1054	1,2	0,8	$C_{27}H_{26}O_{18}$

Scutellarin	462,07983	461,0729	0,8	0,4	$C_{21}H_{18}O_{12}$
Salvianolic acid B_2	718,15338	717,147	1,2	0,9	$C_{36}H_{30}O_{16}$
Caffeoyl tartaric acid	312,04813	311,0413	1,4	0,4	$C_{13}H_{12}O_{9}$
Lithospermic acid_1	538,11113	537,104	0,3	0,2	$C_{27}H_{22}O_{12}$
Rosmarinic acid	360,08452	359,0776	1	0,4	$C_{18}H_{16}O_{8}$
Luteolin	286,04774	285,0407	0,9	0,3	$C_{15}H_{10}O_{6}$
alpha-Methyl-D-mannopyranoside	194,07904	193,0717	-0,2	0	$C_{7}H_{14}O_{6}$
9-F1-phytoprostane	328,22497	327,2176	-0,4	-0,1	$C_{18}H_{32}O_{5}$
ferulic acid-4'-O-glucoside	198,05282	197,0458	1,2	0,2	$C_{9}H_{10}O_{5}$
Dihydroferulic acid 4-O-glucuronide	372,10565	371,0989	1,4	0,5	$C_{16}H_{20}O_{10}$
Apigenin-7-O-glucoside	432,10565	431,0987	0,8	0,3	$C_{21}H_{20}O_{10}$
D-(+)-Mannose	180,06339	179,0562	0,4	0,1	$C_{6}H_{12}O_{6}$
Naringenin-4',5-diglucuronide	608,13773	607,13	-0,7	-0,4	$C_{27}H_{28}O_{16}$
Apigenin	270,05282	269,0456	0,3	0,1	$C_{15}H_{10}O_{5}$
Pinoresinol-4-O-Beta-Monoglycoside	520,19446	519,188	1,6	0,8	$C_{26}H_{32}O_{11}$
Ferulic acid-4'-O-glucoside	356,11073	355,1035	0,1	0	$C_{16}H_{20}O_{9}$
Quercitrin (Quercetin-3-O-alpha-L-rhamnopyranoside)	448,10056	447,0941	1,8	0,8	$C_{21}H_{20}O_{11}$

4-Hydroxy-5-(3',5'-dihydroxyphenyl)-valeric acid-O-glucuronide	402,11621	401,1109	4,9	2	$C_{17}H_{22}O_{11}$
Isorhamnetin 3-O-galactoside	478,11113	477,104	0,3	0,1	$C_{22}H_{22}O_{12}$
Hispidulin glucuronide	476,09548	475,0886	0,9	0,4	$C_{22}H_{20}O_{12}$
Luteolin-3-O-glucuronide	462,07983	461,0732	1,4	0,6	$C_{21}H_{18}O_{12}$
Quercetin 3'-O-glucuronide	478,07474	477,0678	0,7	0,4	$C_{21}H_{18}O_{13}$
Chicoric acid	474,07983	473,0735	2	0,9	$C_{22}H_{18}O_{12}$
Melittoside	524,17412	523,165	-3,5	-1,9	$C_{21}H_{32}O_{15}$
Oleuropein	540,18429	539,176	-1,8	-1	$C_{25}H_{32}O_{13}$
Theaflavin 3-O-gallate_1	716,13773	715,1317	1,7	1,2	$C_{36}H_{28}O_{16}$
Aucubin	346,12638	345,1198	1,9	0,7	$C_{15}H_{22}O_{9}$
Silybin	482,1213	481,1124	-3,3	-1,6	$C_{25}H_{22}O_{10}$

Table S7. Identified compounds in lavender aqueous extract (ESI positive mode).

Component name	Neutral mass (Da)	Observed m/z	Mass error (ppm)	Mass error (mDa)	Formula
Luteolin 7-O-diglucuronide_1	638,11191	639,119	-0,3	-0,2	$C_{27}H_{26}O_{18}$
Scutellarin	462,07983	463,0874	0,7	0,3	$C_{21}H_{18}O_{12}$

Apigenin-7-O-glucoside	432,10565	433,1133	0,8	0,4	$C_{21}H_{20}O_{10}$
Sucrose	342,1$_{16}$21	365,105	-1,1	-0,4	$C_{12}H_{22}O_{11}$
Hexose	180,06339	203,0522	-1,9	-0,4	$C_{6}H_{12}O_{6}$
Pinoresinol-4-O-Beta-Monoglycoside	520,19446	543,1846	1,7	0,9	$C_{26}H_{32}O_{11}$
Salvianolic acid B_2	718,15338	741,1436	1,4	1	$C_{36}H_{30}O_{16}$
Caffeoyl tartaric acid	312,04813	335,0371	-0,8	-0,3	$C_{13}H_{12}O_{9}$
Cynarin	5$_{16}$,12678	539,1187	5	2,7	$C_{25}H_{24}O_{12}$
Hispidulin glucuronide	476,09548	477,1043	3,2	1,5	$C_{22}H_{20}O_{12}$
Astragalin	448,10056	449,1085	1,6	0,7	$C_{21}H_{20}O_{11}$
Acteoside	624,20542	647,1955	1,3	0,8	$C_{29}H_{36}O_{15}$
Luteolin 4'-glucoside	448,10056	471,0903	1	0,5	$C_{21}H_{20}O_{11}$
Salvianolic acid C	492,10565	493,1134	0,9	0,5	$C_{26}H_{20}O_{10}$
Kaempferol 3-rhamnosyl-(1->2)-rhamnosyl-(1->6)-glucoside	740,2$_{16}$38	763,2092	4,8	3,6	$C_{33}H_{40}O_{19}$
5-Feruloylquinic acid	368,11073	391,1005	1,3	0,5	$C_{17}H_{20}O_{9}$
beta-D-Glcp-(1->4)-[L-alpha-D-Hepp-(1->3)]-L-alpha-D-Hepp	564,190$_{16}$	587,1796	0,3	0,2	$C_{20}H_{36}O_{18}$

alpha-Methyl-D-mannopyranoside	194,07904	194,079	2,7	0,5	$C_7H_{14}O_6$
5-O-Caffeoylquinic acid	354,09508	377,0861	4,7	1,8	$C_{16}H_{18}O_9$
Isoscutellarein 4'-methyl ether 7-(6'''-acetylallosyl)(1->2)-glucoside	666,1796	667,1901	4,8	3,2	$C_{30}H_{34}O_{17}$

Table S8. Identified compounds in lemon balm aqueous extract (ESI negative mode).

Component name	Neutral mass (Da)	Observed m/z	Mass error (ppm)	Mass error (mDa)	Formula
Lithospermic acid B	718,15338	717,1465	0,6	0,4	$C_{36}H_{30}O_{16}$
Rosmarinic acid	360,08452	359,0778	1,5	0,5	$C_{18}H_{16}O_8$
Theaflavin 3-O-gallate_1	716,13773	715,1308	0,5	0,4	$C_{36}H_{28}O_{16}$
Chicoric acid	474,07983	473,0731	1,1	0,5	$C_{22}H_{18}O_{12}$
Caffeoyl tartaric acid	312,04813	311,0411	0,7	0,2	$C_{13}H_{12}O_9$
Salvianolic acid B_1	718,15338	717,1464	0,4	0,3	$C_{36}H_{30}O_{16}$
9-F1-phytoprostane	328,22497	327,2183	1,9	0,6	$C_{18}H_{32}O_5$
Salvianolic acid B_2	718,15338	717,1462	0,1	0,1	$C_{36}H_{30}O_{16}$
Luteolin	286,04774	285,0407	0,7	0,2	$C_{15}H_{10}O_6$
Vanillylmandelic acid	198,05282	197,0456	0	0	$C_9H_{10}O_5$
Genkwanin	284,06847	283,0612	-0,2	0	$C_{16}H_{12}O_5$

Caffeic acid 4-O-glucoside	342,09508	341,0877	-0,2	-0,1	$C_{15}H_{18}O_9$
Salvianolic Acid A	494,1213	493,1137	-0,6	-0,3	$C_{26}H_{22}O_{10}$
1,3-Dicaffeoylquinic acid	516,12678	515,1184	-2,1	-1,1	$C_{25}H_{24}O_{12}$
Nicotiflorin	594,15847	593,1505	-1,2	-0,7	$C_{26}H_{30}O_{15}$
Isorhamnetin 3-O-galactoside	478,11113	477,1044	1,1	0,5	$C_{22}H_{22}O_{12}$
Quercitrin (Quercetin-3-O-alpha-L-rhamnopyranoside)	448,10056	447,093	-0,6	-0,3	$C_{21}H_{20}O_{11}$
Naringenin-4',5-diglucuronide	608,13773	607,129	-2,4	-1,4	$C_{26}H_{28}O_{16}$
Quercetin 3-O-beta-D-glucopyranosyl-7-O-alpha-L-rhamnopyranoside	610,15338	609,1443	-2,9	-1,8	$C_{26}H_{30}O_{16}$
Kaempferol 3-O-(6''-acetyl-galactoside) 7-O-rhamnoside	636,16903	635,1596	-3,3	-2,1	$C_{29}H_{32}O_{16}$
Diosmetin	300,06339	299,0558	-1,2	-0,4	$C_{16}H_{12}O_6$
Dihydroferulic acid-4'-O-glucuronide	372,10565	371,0974	-2,6	-0,9	$C_{16}H_{20}O_{10}$
Leucosceptoside A	638,22107	637,2151	2,1	1,3	$C_{30}H_{38}O_{15}$
6''-O-Malonyldaidzin	502,11113	501,1042	0,7	0,4	$C_{24}H_{22}O_{12}$
Acteoside	624,20542	623,2007	4,1	2,6	$C_{29}H_{36}O_{15}$

Table S9. Identified compounds in lemon balm aqueous extract (ESI positive mode).

Component name	Neutral mass (Da)	Observed m/z	Mass error (ppm)	Mass error (mDa)	Formula
Luteolin-3-O-glucuronide	462,07983	463,0868	-0,7	-0,3	$C_{21}H_{18}O_{12}$
9-F1-phytoprostane	328,22497	351,2135	-1,9	-0,7	$C_{18}H_{32}O_{5}$
Sucrose	342,11621	365,1047	-2	-0,7	$C_{12}H_{22}O_{11}$
Luteolin	286,04774	287,0547	-1,1	-0,3	$C_{15}H_{10}O_{6}$
Luteolin 7-O-glucoside	448,10056	449,1081	0,6	0,3	$C_{21}H_{20}O_{11}$
Hexose	180,06339	203,0522	-2,1	-0,4	$C_{6}H_{12}O_{6}$
Salvianolic acid B_1	718,15338	741,1425	-0,2	-0,1	$C_{36}H_{30}O_{16}$
Caffeoyl tartaric acid	312,04813	335,0371	-0,7	-0,2	$C_{13}H_{12}O_{9}$
Theaflavin 3-O-gallate_1	716,13773	739,1272	0,3	0,2	$C_{36}H_{28}O_{16}$
Luteolin 4'-glucoside	448,10056	449,1078	0	0	$C_{21}H_{20}O_{11}$
Nicotiflorin	594,15847	595,1649	-1,3	-0,8	$C_{27}H_{30}O_{15}$
Lithospermic acid B	718,15338	741,1439	1,8	1,3	$C_{36}H_{30}O_{16}$
Luteolin 7-O-diglucuronide_1	638,11191	639,1209	2,7	1,7	$C_{27}H_{26}O_{18}$
Lithospermic acid_1	538,11113	539,119	1	0,6	$C_{27}H_{22}O_{12}$

Hydroxytyrosol 4-O-glucoside	316,11582	339,1043	-2,3	-0,8	$C_{14}H_{20}O_8$
Acteoside	624,20542	625,2105	-3,6	-2,2	$C_{29}H_{36}O_{15}$
Leucosceptoside A	638,22107	661,2089	-2,1	-1,4	$C_{30}H_{38}O_{15}$
Silydianin	482,1213	483,1273	-2,6	-1,3	$C_{25}H_{22}O_{10}$
Manghaslin (Quercetin 3-2G-rhamnosylrutinoside)	756,21129	779,2003	-0,3	-0,2	$C_{33}H_{40}O_{20}$
Echinacoside	786,25824	809,2456	-2,3	-1,9	$C_{35}H_{46}O_{20}$
Vanillylmandelic acid	198,05282	221,0411	-4,4	-1	$C_9H_{10}O_5$
Achillolide A	306,11034	329,1002	2,1	0,7	$C_{16}H_{18}O_6$
Silybin	482,1213	505,11	-1	-0,5	$C_{25}H_{22}O_{10}$
Glucobrassicanapin	387,06577	388,0724	-1,6	-0,6	$C_{12}H_{21}NO_9S_2$

Table S10. Identified compounds in lemon peel aqueous extract (ESI negative mode).

Component name	Neutral mass (Da)	Observed m/z	Mass error (ppm)	Mass error (mDa)	Formula
Eriocitrin_1	596,17412	595,1646	-3,7	-2,2	$C_{27}H_{32}O_{15}$
Isoscutellarein 7-O-[6‴-O-acetyl-β-d-allopyranosyl-(1→2)]-β-d-glucopyranoside	652,16395	651,1571	0,7	0,4	$C_{29}H_{32}O_{17}$
Nicotiflorin	594,15847	593,1519	1,2	0,7	$C_{27}H_{30}O_{15}$
Hesperidin	610,18977	609,1825	0	0	$C_{28}H_{34}O_{15}$
Rutin	610,15338	609,1471	1,6	1	$C_{27}H_{30}O_{16}$

6''-O-Malonyldaidzin	502,11113	501,1042	0,8	0,4	$C_{24}H_{22}O_{12}$
Isorhamnetin 3-O-glucoside 7-O-rhamnoside	624,16903	623,1621	0,5	0,3	$C_{28}H_{32}O_{16}$
D-(+)-Mannose	180,06339	179,0563	0,9	0,2	$C_{6}H_{12}O_{6}$
Salvianolic acid G	418,09	417,0828	0,1	0	$C_{20}H_{18}O_{10}$
Diosmin	608,17412	607,1671	0,4	0,2	$C_{28}H_{32}O_{15}$
Chrysoeriol	300,06339	299,056	-0,5	-0,2	$C_{16}H_{12}O_{6}$
Limonin	470,19407	469,1868	0	0	$C_{26}H_{30}O_{8}$
Orientin	448,10056	447,0937	1	0,4	$C_{21}H_{20}O_{11}$
9-F1-phytoprostane	328,22497	327,2175	-0,6	-0,2	$C_{18}H_{32}O_{5}$
Chrysoeriol 7-O-apiosyl-glucoside	594,15847	593,1517	0,9	0,5	$C_{27}H_{30}O_{15}$
Azadirachtin	720,26294	719,2533	-3,2	-2,3	$C_{35}H_{44}O_{16}$
Isorhamnetin-3-O-rutinoside	624,16903	623,1604	-2,2	-1,3	$C_{28}H_{32}O_{16}$
Hesperetin	302,07904	301,0713	-1,4	-0,4	$C_{16}H_{14}O_{6}$
Ferulic acid-4'-O-glucoside	356,11073	355,1051	4,5	1,6	$C_{16}H_{20}O_{9}$
Isorhamnetin 3-O-galactoside	478,11113	477,1042	0,7	0,3	$C_{22}H_{22}O_{12}$
Astilbin	450,11621	449,1089	-0,1	-0,1	$C_{21}H_{22}O_{11}$
4-Hydroxy-5-(3',5'-dihydroxyphenyl)-valeric acid-O-glucuronide	402,11621	401,1092	0,7	0,3	$C_{17}H_{22}O_{11}$
Naringenin-4',5-diglucuronide	608,13773	607,1275	-4,9	-3	$C_{27}H_{28}O_{16}$
Barbatoside A/B	664,22146	663,2146	0,6	0,4	$C_{28}H_{40}O_{18}$
Allobetonicoside	506,16356	505,1562	-0,1	-0,1	$C_{21}H_{30}O_{14}$
6''-O-Malonylgenistin	518,10604	517,0993	0,9	0,5	$C_{24}H_{22}O_{13}$

kaempferol 3-O-rutinoside	594,15847	593,1522	1,6	1	$C_{27}H_{30}O_{15}$
Quercetin-3-O-glucoside	464,09548	463,0885	0,6	0,3	$C_{21}H_{20}O_{12}$
Antoside	610,15338	609,1449	-2	-1,2	$C_{27}H_{30}O_{16}$
Isorhamnetin 4'-O-glucuronide	492,09039	491,0826	-1,1	-0,5	$C_{22}H_{20}O_{13}$
Kaempferol 3-O-(6''-acetyl-galactoside) 7-O-rhamnoside	636,16903	635,1595	-3,5	-2,2	$C_{29}H_{32}O_{16}$
Isorhamnetin 3-O-glucoside	478,11113	477,1022	-3,4	-1,6	$C_{22}H_{22}O_{12}$
;Homoplantaginin_Tectoridin	462,11621	461,1072	-3,7	-1,7	$C_{22}H_{22}O_{11}$
Pinoresinol-4-O-Beta-Monoglycoside	520,19446	519,1866	-1,1	-0,6	$C_{26}H_{32}O_{11}$
Geniposidic-Acid	374,1213	373,1141	0,2	0,1	$C_{16}H_{22}O_{10}$
Luteolin 7-O-glucoside	448,10056	447,0933	0,1	0,1	$C_{21}H_{20}O_{11}$
Apigenin 7-O-apiosyl-glucoside	564,14791	563,1395	-2	-1,1	$C_{26}H_{28}O_{14}$
Citric acid	192,027	191,0194	-1,6	-0,3	$C_6H_8O_7$
Hispidulin glucuronide	476,09548	475,0871	-2,4	-1,1	$C_{22}H_{20}O_{12}$
Rosmarinic acid	360,08452	359,079	4,9	1,7	$C_{18}H_{16}O_8$

Table S11. Identified compounds in lemon peel aqueous extract (ESI positive mode).

Component name	Neutral mass (Da)	Observed m/z	Mass error (ppm)	Mass error (mDa)	Formula
Peonidin 3-O-sophoroside	625,17686	625,1766	0,5	0,3	$C_{28}H_{33}O_{16}$
Byakangelicin	334,10525	357,0936	-2,4	-0,8	$C_{17}H_{18}O_7$

Nicotiflorin	594,15847	595,1661	0,6	0,4	$C_{27}H_{30}O_{15}$
Limonin	470,19407	471,2009	-1	-0,5	$C_{26}H_{30}O_{8}$
Isoscutellarein 7-O-[6′′′-O-acetyl-β-d-allopyranosyl-(1→2)]-β-d-glucopyranoside	652,16395	653,1711	-0,2	-0,1	$C_{29}H_{32}O_{17}$
Eriocitrin_1	596,17412	619,1636	0,4	0,2	$C_{27}H_{32}O_{15}$
Nobiletin	402,13147	403,1381	-1,6	-0,7	$C_{21}H_{22}O_{8}$
Hesperidin	610,18977	633,1788	-0,4	-0,2	$C_{28}H_{34}O_{15}$
Rutin	610,15338	611,1615	1,3	0,8	$C_{27}H_{30}O_{16}$
Isorhamnetin 3-O-glucoside 7-O-rhamnoside	624,16903	625,177	1,1	0,7	$C_{28}H_{32}O_{16}$
1,3-Dicaffeoylquinic acid	516,12678	517,1322	-3,5	-1,8	$C_{25}H_{24}O_{12}$
Chrysoeriol 7-O-apiosyl-glucoside	594,15847	595,1663	1	0,6	$C_{27}H_{30}O_{15}$
Myricetin-3-O-α-L-rhamnopyranoside	464,09548	465,103	0,5	0,2	$C_{21}H_{20}O_{12}$
Coumaroyl tartaric acid (p-)	296,05322	297,0607	0,8	0,2	$C_{13}H_{12}O_{8}$
Isorhamnetin-3-O-rutinoside	624,16903	647,1587	0,7	0,5	$C_{28}H_{32}O_{16}$
Quercetin 3-O-beta-D-glucopyranosyl-7-O-alpha-L-rhamnopyranoside	610,15338	611,1619	2	1,2	$C_{27}H_{30}O_{16}$

Spinacetin 3-O-glucosyl-(1->6)-glucoside	670,17451	670,1764	3,7	2,5	$C_{29}H_{34}O_{18}$
Quercetin 3-O-(6-malonyl-glucoside)"	550,09587	551,1027	-0,8	-0,4	$C_{24}H_{22}O_{15}$
4-O-Caffeoylquinic acid	354,09508	377,0839	-1,2	-0,4	$C_{16}H_{18}O_{9}$
Cirsimaritin	314,07904	315,085	-4,3	-1,4	$C_{17}H_{14}O_{6}$
Astilbin	450,11621	473,1043	-2,4	-1,1	$C_{21}H_{22}O_{11}$
Pinoresinol-4-O-Beta-Monoglycoside	520,19446	520,1923	-3,2	-1,7	$C_{26}H_{32}O_{11}$

Table S12. Identified compounds in rosehip aqueous extract (ESI negative mode).

Component name	Neutral mass (Da)	Observed m/z	Mass error (ppm)	Mass error (mDa)	Formula
Astilbin	450,11621	449,1091	0,3	0,2	$C_{21}H_{22}O_{11}$
Valoneic acid dilactone	470,01214	469,0048	-0,1	-0,1	$C_{21}H_{10}O_{13}$
5-O-Galloylquinic acid	344,07435	343,0676	1,5	0,5	$C_{14}H_{16}O_{10}$
Protocatechuic acid 4-O-glucoside	316,07943	315,0724	0,9	0,3	$C_{13}H_{16}O_{9}$
Nicotiflorin	594,15847	593,1484	-4,7	-2,8	$C_{27}H_{30}O_{15}$
Taxifolin	304,0583	303,0509	-0,5	-0,2	$C_{15}H_{12}O_{7}$

Ascorbic acid (L-)	176,03209	175,025	1	0,2	$C_6H_8O_6$
Quercetin 3-arabinoside	434,08491	433,0776	-0,2	-0,1	$C_{20}H_{18}O_{11}$
Sucrose	342,11621	341,1095	1,6	0,6	$C_{12}H_{22}O_{11}$
Dihydroferulic acid 4-O-glucuronide	372,10565	371,0972	-3,2	-1,2	$C_{16}H_{20}O_{10}$
Melittoside	524,17412	523,1661	-1,5	-0,8	$C_{21}H_{32}O_{15}$
Diosmin	608,17412	607,1659	-1,5	-0,9	$C_{28}H_{32}O_{15}$
Apigenin 7-O-diglucuronide	622,117	621,1095	-0,3	-0,2	$C_{27}H_{26}O_{17}$
Dihydroferulic acid-4'-O-glucuronide	372,10565	371,099	1,8	0,7	$C_{16}H_{20}O_{10}$

Table S13. Identified compounds in rosehip aqueous extract (ESI positive mode).

Component name	Neutral mass (Da)	Observed m/z	Mass error (ppm)	Mass error (mDa)	Formula
Hexose	180,06339	203,0525	-0,7	-0,1	$C_6H_{12}O_6$
Valoneic acid dilactone	470,01214	471,0189	-1,1	-0,5	$C_{21}H_{10}O_{13}$
Astilbin	450,11621	451,1234	-0,3	-0,1	$C_{21}H_{22}O_{11}$
Teupolioside	786,25824	786,2544	-4,2	-3,3	$C_{35}H_{46}O_{20}$
9-F1-phytoprostane	328,22497	351,2131	-3,1	-1,1	$C_{18}H_{32}O_5$

alpha-Methyl-D-mannopyranoside	194,07904	217,0692	4,1	0,9	$C_7H_{14}O_6$
Genistein 4',7-O-diglucuronide	622,117	623,1232	-1,7	-1	$C_{27}H_{26}O_{17}$
6''-O-Malonylgenistin	518,10604	541,0926	-4,9	-2,6	$C_{24}H_{22}O_{13}$

Table S14. Identified compounds in sideritis aqueous extract (ESI negative mode).

Component name	Neutral mass (Da)	Observed m/z	Mass error (ppm)	Mass error (mDa)	Formula
Acteoside	624,20542	623,198	-0,2	-0,1	$C_{29}H_{36}O_{15}$
Isoscutellarein 4'-methyl ether 7-(6'''-acetylallosyl)(1->2)-glucoside	666,1796	665,1727	0,5	0,4	$C_{30}H_{34}O_{17}$
9-F1-phytoprostane	328,22497	327,2178	0,4	0,1	$C_{18}H_{32}O_5$
Isoscutellarein 7-O-[6‴-O-acetyl-β-d-allopyranosyl-(1→2)]-β-d-glucopyranoside	652,16395	651,1574	1,1	0,7	$C_{29}H_{32}O_{17}$
Apigenin 7-(4''-E-p-coumarylglucoside)	578,14243	577,1352	0,2	0,1	$C_{30}H_{26}O_{12}$
Apigenin-7-O-glucoside	432,10565	431,0984	0,1	0	$C_{21}H_{20}O_{10}$
Teupolioside	786,25824	785,25	-1,3	-1	$C_{35}H_{46}O_{20}$

Bergenin	328,07943	327,0722	0,2	0,1	$C_{14}H_{16}O_9$
Apigenin	270,05282	269,0458	0,9	0,2	$C_{15}H_{10}O_5$
Cirsilineol_1	344,0896	343,0825	0,4	0,2	$C_{18}H_{16}O_7$
Kaempferol 3-O-sophoroside	610,15338	609,1466	0,8	0,5	$C_{27}H_{30}O_{16}$
Leucosceptoside A	638,22107	637,2128	-1,6	-1	$C_{30}H_{38}O_{15}$
Cirsilineol_2	344,0896	343,0824	0,2	0,1	$C_{18}H_{16}O_7$
5-Feruloylquinic acid	368,11073	367,1035	0,1	0	$C_{17}H_{20}O_9$
kaempferol 3-O-rutinoside	594,15847	593,1512	0,1	0	$C_{27}H_{30}O_{15}$
Pectolinarigenin	314,07904	313,0716	-0,6	-0,2	$C_{17}H_{14}O_6$
Pinoresinol-4-O-Beta-Monoglycoside	520,19446	519,1862	-1,9	-1	$C_{26}H_{32}O_{11}$
Isoscutellarein 7-O-[6'-O-acetyl-β-D-allopyranosyl-(1→2)]-6''-O-acetyl-β-D-glucopyranoside''	694,17451	693,167	-0,3	-0,2	$C_{31}H_{34}O_{18}$
Geniposidic-Acid	374,1213	373,1138	-0,5	-0,2	$C_{16}H_{22}O_{10}$
Genistein 4',7-O-diglucuronide	622,117	621,1091	-1,1	-0,7	$C_{27}H_{26}O_{17}$
Luteolin 7-O-diglucuronide_2	638,11191	637,103	-2,5	-1,6	$C_{27}H_{26}O_{18}$
Hesperidin	610,18977	609,1826	0,2	0,1	$C_{28}H_{34}O_{15}$
Nicotiflorin	594,15847	593,1495	-2,9	-1,7	$C_{27}H_{30}O_{15}$
Rhamnetin 3-glucoside	478,11113	477,1037	-0,4	-0,2	$C_{22}H_{22}O_{12}$

Luteolin 4'-glucoside	448,10056	447,0931	-0,3	-0,2	$C_{21}H_{20}O_{11}$
3,4-Dicaffeoylquinic Acid	516,12678	515,1195	0	0	$C_{25}H_{24}O_{12}$
4-Hydroxy-5-(3',5'-dihydroxyphenyl)-valeric acid-O-glucuronide	402,11621	401,108	-2,4	-1	$C_{17}H_{22}O_{11}$
Lithospermic acid B	718,15338	717,1463	0,2	0,2	$C_{36}H_{30}O_{16}$
Falcarindiol 3-acetate	302,18819	301,1805	-1,4	-0,4	$C_{19}H_{26}O_{3}$
Antoside	610,15338	609,1452	-1,5	-0,9	$C_{27}H_{30}O_{16}$
Luteolin 7-O-diglucuronide_1	638,11191	637,102	-4,2	-2,7	$C_{27}H_{26}O_{18}$
Apigenin 7-O-apiosyl-glucoside	564,14791	563,1404	-0,5	-0,3	$C_{26}H_{28}O_{14}$
Kaempferol 3-O-acetyl-glucoside	490,11113	489,1029	-2	-1	$C_{23}H_{22}O_{12}$
Citric acid	192,027	191,0194	-1,9	-0,4	$C_{6}H_{8}O_{7}$

Table S15. Identified compounds in sideritis aqueous extract (ESI positive mode).

Component name	Neutral mass (Da)	Observed m/z	Mass error (ppm)	Mass error (mDa)	Formula
Isoscutellarein 4'-methyl ether 7-(6'''-acetylallosyl)(1->2)-glucoside	666,1796	667,1872	0,5	0,3	$C_{30}H_{34}O_{17}$
9-F1-phytoprostane	328,22497	351,2132	-3	-1	$C_{18}H_{32}O_{5}$

Isoscutellarein 7-O-[6′′′-O-acetyl-β-d-allopyranosyl-(1→2)]-β-d-glucopyranoside	652,16395	653,1717	0,8	0,5	$C_{29}H_{32}O_{17}$
Acteoside	624,20542	647,1952	0,9	0,6	$C_{29}H_{36}O_{15}$
Apigenin-7-O-glucoside	432,10565	433,113	0,2	0,1	$C_{21}H_{20}O_{10}$
Cirsilineol_1	344,0896	345,0963	-1,7	-0,6	$C_{18}H_{16}O_{7}$
Teupolioside	786,25824	809,2472	-0,3	-0,3	$C_{35}H_{46}O_{20}$
Isoacteoside	624,20542	647,1975	4,3	2,8	$C_{29}H_{36}O_{15}$
Cirsilineol_2	344,0896	345,0962	-2	-0,7	$C_{18}H_{16}O_{7}$
Leucosceptoside A	638,22107	661,2105	0,3	0,2	$C_{30}H_{38}O_{15}$
5-Feruloylquinic acid	368,11073	391,0997	-0,8	-0,3	$C_{17}H_{20}O_{9}$
Bergenin	328,07943	351,0681	-1,4	-0,5	$C_{14}H_{16}O_{9}$
Apigenin 7-(2'',3''-diacetyl-hexocide)	516,12678	517,1342	0,2	0,1	$C_{25}H_{24}O_{12}$
1,3-Dicaffeoylquinic acid	516,12678	517,135	1,8	1	$C_{25}H_{24}O_{12}$
Oleuropein	540,18429	563,1726	-1,7	-1	$C_{25}H_{32}O_{13}$
alpha-Methyl-D-mannopyranoside	194,07904	217,0684	0,5	0,1	$C_{7}H_{14}O_{6}$
Luteolin 7-O-diglucuronide_1	638,11191	639,1185	-1,1	-0,7	$C_{27}H_{26}O_{18}$

Table S16. Identified compounds in spearmint aqueous extract (ESI negative mode).

Component name	Neutral mass (Da)	Observed m/z	Mass error (ppm)	Mass error (mDa)	Formula
Nicotiflorin	594,15847	593,151	-0,3	-0,2	$C_{27}H_{30}O_{15}$
Scutellarin	462,07983	461,073	1	0,5	$C_{21}H_{18}O_{12}$
Rosmarinic acid	360,08452	359,0778	1,6	0,6	$C_{18}H_{16}O_{8}$
Diosmin	608,17412	607,1677	1,4	0,8	$C_{28}H_{32}O_{15}$
Luteolin-3-O-glucuronide	462,07983	461,0741	3,3	1,5	$C_{21}H_{18}O_{12}$
Luteolin	286,04774	285,0407	0,9	0,3	$C_{15}H_{10}O_{6}$
Hesperidin	610,18977	609,183	0,8	0,5	$C_{28}H_{34}O_{15}$
Vanillylmandelic acid	198,05282	197,0456	0,5	0,1	$C_{9}H_{10}O_{5}$
Caffeoyl tartaric acid	312,04813	311,0413	1,4	0,4	$C_{13}H_{12}O_{9}$
Isorhamnetin 3-O-glucoside 7-O-rhamnoside	624,16903	623,1605	-2	-1,3	$C_{28}H_{32}O_{16}$
Salvianolic acid B_1	718,15338	717,1461	0	0	$C_{36}H_{30}O_{16}$
Salvianolic Acid A	494,1213	493,1148	1,7	0,8	$C_{26}H_{22}O_{10}$
Apigenin-7-O-glucuronide	446,08491	445,0786	2,2	1	$C_{21}H_{18}O_{11}$
Lithospermic acid B	718,15338	717,1456	-0,8	-0,5	$C_{36}H_{30}O_{16}$
Rutin	610,15338	609,1463	0,4	0,2	$C_{27}H_{30}O_{16}$

Luteolin 7-O-diglucuronide_1	638,11191	637,1051	0,7	0,4	$C_{27}H_{26}O_{18}$
Luteolin 7-O-glucoside	448,10056	447,0934	0,3	0,2	$C_{21}H_{20}O_{11}$
Eupatilin	344,0896	343,0826	0,7	0,2	$C_{18}H_{16}O_{7}$
Chicoric acid	474,07983	473,0729	0,7	0,3	$C_{22}H_{18}O_{12}$
Genkwanin	284,06847	283,0615	1	0,3	$C_{16}H_{12}O_{5}$
kaempferol 3-O-rutinoside	594,15847	593,1504	-1,3	-0,8	$C_{27}H_{30}O_{15}$
Isorhamnetin 3-O-glucoside	478,11113	477,1034	-0,9	-0,5	$C_{22}H_{22}O_{12}$
Chrysoeriol 7-O-apiosyl-glucoside	594,15847	593,1507	-0,8	-0,5	$C_{27}H_{30}O_{15}$
Cynarin	5_{16},12678	515,1186	-1,7	-0,9	$C_{25}H_{24}O_{12}$
Ferulic acid-4'-O-glucoside	356,11073	355,1034	-0,3	-0,1	$C_{16}H_{20}O_{9}$
1,3-Dicaffeoylquinic acid	5_{16},12678	515,1191	-0,8	-0,4	$C_{25}H_{24}O_{12}$
3,4-Dicaffeoylquinic Acid	5_{16},12678	515,1179	-3,1	-1,6	$C_{25}H_{24}O_{12}$
Antoside	610,15338	609,1452	-1,5	-0,9	$C_{27}H_{30}O_{16}$
Apigenin 7-O-diglucuronide	622,117	621,1112	2,4	1,5	$C_{27}H_{26}O_{17}$
Naringenin-4',5-diglucuronide	608,13773	607,1275	-4,9	-3	$C_{27}H_{28}O_{16}$

Kaempferol 3-rhamnosyl-(1->2)-rhamnosyl-(1->6)-glucoside	740,21638	739,2078	-1,7	-1,3	$C_{33}H_{40}O_{19}$
Oleuropein	540,18429	539,1763	-1,3	-0,7	$C_{25}H_{32}O_{13}$
Allobetonicoside	506,16356	505,1561	-0,4	-0,2	$C_{21}H_{30}O_{14}$
Acteoside	624,20542	623,2007	4,1	2,5	$C_{29}H_{36}O_{15}$
6''-O-Malonyldaidzin	502,11113	501,1034	-0,8	-0,4	$C_{24}H_{22}O_{12}$
Salvianolic acid G	418,09	417,082	-1,7	-0,7	$C_{20}H_{18}O_{10}$
Quercetin 3-arabinoside	434,08491	433,0779	0,6	0,3	$C_{20}H_{18}O_{11}$
Melittoside	524,17412	523,1679	2	1,1	$C_{21}H_{32}O_{15}$

Table S17. Identified compounds in spearmint aqueous extract (ESI positive mode).

Component name	Neutral mass (Da)	Observed m/z	Mass error (ppm)	Mass error (mDa)	Formula
Scutellarin	462,07983	463,0871	0	0	$C_{21}H_{18}O_{12}$
Diosmin	608,17412	609,1817	0,5	0,3	$C_{28}H_{32}O_{15}$
Luteolin-3-O-glucuronide	462,07983	463,0881	2,1	1	$C_{21}H_{18}O_{12}$
Luteolin 7-O-rutinoside	594,15847	595,166	0,5	0,3	$C_{27}H_{30}O_{15}$
9-F1-phytoprostane	328,22497	351,2137	-1,3	-0,4	$C_{18}H_{32}O_{5}$
Luteolin	286,04774	287,0548	-0,6	-0,2	$C_{15}H_{10}O_{6}$

Luteolin 7-O-glucoside	448,10056	449,1079	0	0	$C_{21}H_{20}O_{11}$
Hesperidin	610,18977	633,1791	0,1	0,1	$C_{28}H_{34}O_{15}$
Sucrose	342,11621	365,1052	-0,7	-0,3	$C_{12}H_{22}O_{11}$
Luteolin 7-O-diglucuronide_1	638,11191	639,1197	0,8	0,5	$C_{27}H_{26}O_{18}$
Hexose	180,06339	203,0525	-0,7	-0,1	$C_{6}H_{12}O_{6}$
Salvianolic acid B_1	718,15338	741,1424	-0,3	-0,2	$C_{36}H_{30}O_{16}$
Rutin	610,15338	611,1612	0,9	0,6	$C_{27}H_{30}O_{16}$
3,4-Dicaffeoylquinic Acid	516,12678	539,118	3,7	2	$C_{25}H_{24}O_{12}$
Acteoside	624,20542	647,1942	-0,7	-0,4	$C_{29}H_{36}O_{15}$
Chrysoeriol 7-O-apiosyl-glucoside	594,15847	595,1663	0,9	0,5	$C_{27}H_{30}O_{15}$
Rhoifolin	578,16356	578,1641	1,8	1,1	$C_{27}H_{30}O_{14}$
Manghaslin (Quercetin 3-2G-rhamnosylrutinoside)	756,21129	757,2183	-0,3	-0,2	$C_{33}H_{40}O_{20}$
Naringenin-4',5-diglucuronide	608,13773	609,145	0	0	$C_{27}H_{28}O_{16}$
5-O-Caffeoylshikimic acid	336,08452	359,0747	2,8	1	$C_{16}H_{16}O_{8}$
kaempferol 3-O-rutinoside	594,15847	617,1468	-1,5	-0,9	$C_{27}H_{30}O_{15}$
Lithospermic acid_1	538,11113	561,0992	-2,1	-1,2	$C_{27}H_{22}O_{12}$
Quercetin 3-rutinoside-7-glucoside	772,20621	795,1929	-3,1	-2,5	$C_{33}H_{40}O_{21}$
Vanillylmandelic acid	198,05282	221,0411	-4,4	-1	$C_{9}H_{10}O_{5}$

Table S18. Identified compounds in St. John's wort aqueous extract (ESI negative mode).

Component name	Neutral mass (Da)	Observed m/z	Mass error (ppm)	Mass error (mDa)	Formula
Quercetin-3-O-glucoside	464,09548	463,0886	0,8	0,4	$C_{21}H_{20}O_{12}$
Quercetin	302,04265	301,0354	-0,1	0	$C_{15}H_{10}O_{7}$
Luteolin 4'-glucoside	448,10056	447,0932	-0,3	-0,1	$C_{21}H_{20}O_{11}$
Cinnamtannin A2_1	1154,2692	1153,2608	-0,9	-1,1	$C_{60}H_{50}O_{24}$
9-F1-phytoprostane	328,22497	327,2176	-0,2	-0,1	$C_{18}H_{32}O_{5}$
Astragalin	448,10056	447,0932	-0,1	0	$C_{21}H_{20}O_{11}$
Quercetin 3,4'-O-diglucoside	626,1483	625,1412	0,2	0,1	$C_{27}H_{30}O_{17}$
Anhydrochiisanogenoic acid	502,32944	501,3219	-0,6	-0,3	$C_{30}H_{46}O_{6}$
Amentoflavone	538,09	537,0825	-0,4	-0,2	$C_{30}H_{18}O_{10}$
Cinnamtannin A2_2	1154,2692	1153,2605	-1,2	-1,4	$C_{60}H_{50}O_{24}$
Kaempferol	286,04774	285,0405	0	0	$C_{15}H_{10}O_{6}$
Rutin	610,15338	609,1464	0,5	0,3	$C_{27}H_{30}O_{16}$
Oleuropein	540,18429	539,1769	-0,3	-0,2	$C_{25}H_{32}O_{13}$
Kaempferol 3-O-sophoroside	610,15338	609,1442	-3,1	-1,9	$C_{27}H_{30}O_{16}$
Apigenin-7-O-glucuronide	446,08491	445,0771	-1,2	-0,5	$C_{21}H_{18}O_{11}$
5-O-Caffeoylshikimic acid	336,08452	335,0774	0,4	0,1	$C_{16}H_{16}O_{8}$

Myricetin 7-glucoside	480,09039	479,0811	-4,1	-2	$C_{21}H_{20}O_{13}$
Chicoric acid	474,07983	473,0723	-0,5	-0,2	$C_{22}H_{18}O_{12}$
Quercetin 3'-O-glucuronide	478,07474	477,066	-3,1	-1,5	$C_{21}H_{18}O_{13}$
Dihydroferulic acid-4'-O-glucuronide	372,10565	371,0983	-0,3	-0,1	$C_{16}H_{20}O_{10}$
Allobetonicoside	506,16356	505,1552	-2,2	-1,1	$C_{21}H_{30}O_{14}$
Apigenin-7-O-glucoside	432,10565	431,0972	-2,7	-1,2	$C_{21}H_{20}O_{10}$
Dihydroferulic acid 4-O-glucuronide	372,10565	371,0978	-1,6	-0,6	$C_{16}H_{20}O_{10}$
Coumaroylquinic acid	338,10017	337,0937	2,3	0,8	$C_{16}H_{18}O_{8}$
Salvianolic acid G	418,09	417,0821	-1,4	-0,6	$C_{20}H_{18}O_{10}$

Table S19. Identified compounds in St. John's wort aqueous extract (ESI positive mode).

Component name	Neutral mass (Da)	Observed m/z	Mass error (ppm)	Mass error (mDa)	Formula
Quercetin 3-glucuronate	478,07474	479,0816	-0,9	-0,4	$C_{21}H_{18}O_{13}$
Quercetin	302,04265	303,0496	-1,2	-0,3	$C_{15}H_{10}O_{7}$
Hyperoside	464,09548	465,1023	-0,9	-0,4	$C_{21}H_{20}O_{12}$
Myricetin-3-O-α-L-rhamnopyranoside	464,09548	487,0842	-0,9	-0,5	$C_{21}H_{20}O_{12}$
Naringenin 5-O-glucuronide	448,10056	471,0893	-1	-0,5	$C_{21}H_{20}O_{11}$
Amentoflavone	538,09	539,0969	-0,7	-0,4	$C_{30}H_{18}O_{10}$

Luteolin 4'-glucoside	448,10056	471,0896	-0,3	-0,1	$C_{21}H_{20}O_{11}$
9-F1-phytoprostane	328,22497	351,2133	-2,5	-0,9	$C_{18}H_{32}O_{5}$
Cinnamtannin A2_1	1154,2692	1155,2767	0,2	0,2	$C_{60}H_{50}O_{24}$
Quercetin 3-arabinoside	434,08491	457,0737	-0,9	-0,4	$C_{20}H_{18}O_{11}$
Kaempferol-3-o-glucuronide	462,07983	463,0868	-0,5	-0,3	$C_{21}H_{18}O_{12}$
Hexose	180,06339	203,0524	-0,9	-0,2	$C_{6}H_{12}O_{6}$
Chrysoeriol 7-O-apiosyl-glucoside	594,15847	595,1654	-0,5	-0,3	$C_{27}H_{30}O_{15}$
Cinnamtannin A2_2	1154,2692	1155,2773	0,7	0,8	$C_{60}H_{50}O_{24}$
Rutin	610,15338	633,1423	-0,5	-0,3	$C_{27}H_{30}O_{16}$
Quercetin 3,4'-O-diglucoside	626,1483	627,1567	1,9	1,2	$C_{27}H_{30}O_{17}$
Myricetin-3-O-galactopyranoside	480,09039	503,0797	0,2	0,1	$C_{21}H_{20}O_{13}$
Oleuropein	540,18429	541,1919	0,5	0,3	$C_{25}H_{32}O_{13}$
Pinoresinol-4-O-Beta-Monoglycoside	520,19446	543,1845	1,5	0,8	$C_{26}H_{32}O_{11}$
Caffeic acid	180,04226	181,0498	1,4	0,2	$C_{9}H_{8}O_{4}$
Betonicine	159,08954	160,0962	-4,2	-0,7	$C_{7}H_{13}NO_{3}$
Luteolin-3-O-glucuronide	462,07983	485,0713	4,7	2,3	$C_{21}H_{18}O_{12}$
Phloridzin	436,13695	459,125	-2,5	-1,1	$C_{21}H_{24}O_{10}$

Table S20. Total phytochemical profile of the 9 studied plants.

Total Phytochemical Profile									
**Sr. No.**	**Bitter Orange**	**Dittany**	**Lemon Peel**	**Spearmint**	**Lavender**	**Lemon Balm**	**Sideritis**	**St. John's wort**	**Rosehip**
1	1,2-Disinapoylgentiobiose	3,4-Dicaffeoylquinic Acid	9-F1-phytoprostane	Oleuropein	Rosmarinic acid	Rosmarinic acid	9-F1-phytoprostane	Oleuropein	Ascorbic acid (L-)
2	Isoscutellarein 7-O-[6‴-O-acetyl-β-d-allopyranosyl-(1→2)]-β-d-glucopyranoside	Rosmarinic acid	Quercetin-3-O-glucoside	Luteolin	Luteolin-3-O-glucuronide	Lithospermic acid B	Geniposidic-Acid	Quercetin-3-O-glucoside	Apigenin 7-O-diglucuronide
3	Rhoifolin	Salvianolic acid C	Allobetonicoside	Allobetonicoside	Vanillylmandelic acid	Vanillylmandelic acid	Cirsilineol_1	Allobetonicoside	Melittoside
4	Eriocitrin_1	Lithospermic acid B	D-(+)-Mannose	1,3-Dicaffeoylquinic acid	D-(+)-Mannose	Quercitrin (Quercetin-3-O-alpha-L-rhamnopyranoside)	Bergenin	Astragalin	Valoneic acid dilactone
5	Orientin	Vanillylmandelic acid	Geniposidic-Acid	Luteolin 7-O-glucoside	Quercitrin (Quercetin-3-O-alpha-L-rhamnopyranoside)	Diosmetin	Teupolioside	Rutin	5-O-Galloylquinic acid

6	Salvianolic acid G	Ferulic acid-4'-O-glucoside	Barbatoside A/B	Vanillylmandelic acid	Apigenin	Salvianolic acid B_1	Leucosceptoside A	Amentoflavone	Astilbin
7	Kaempferol 3-O-acetyl-glucoside	Diosmin	Luteolin 7-O-glucoside	Rutin	Ferulic acid-4'-O-glucoside	Nicotiflorin	Lithospermic acid B	Apigenin-7-O-glucuronide	Taxifolin
8	D-(+)-Mannose	Salvianolic acid B_1	Rutin	Rosmarinic acid	Salvianolic acid B_2	Quercetin 3-O-beta-D-glucopyranosyl-7-O-alpha-L-rhamnopyranoside	5-Feruloylquinic acid	Kaempferol	Sucrose
9	Nicotiflorin	Isoacteoside	Limonin	Lithospermic acid B	Luteolin 7-O-diglucuronide_2	1,3-Dicaffeoylquinic acid	Apigenin	Cinnamtannin A2_1	Protocatechuic acid 4-O-glucoside
10	Didymin	6''-O-Malonylgenistin	Rosmarinic acid	Isorhamnetin 3-O-glucoside	Luteolin	Leucosceptoside A	Kaempferol 3-O-sophoroside	Quercetin	Nicotiflorin
11	5-Feruloylquinic acid	Naringenin 7-O-glucoside	Isorhamnetin 3-O-glucoside	Apigenin-7-O-glucuronide	Melittoside	Salvianolic Acid A	Acteoside	Chicoric acid	Diosmin
12	Rutin	Cirsilineol_1	Azadirachtin	Apigenin 7-O-diglucuronide	Theaflavin 3-O-gallate_1	Luteolin	Hesperidin	Apigenin-7-O-glucoside	Quercetin 3-arabinoside
13	3,4-Dicaffeoylquinic Acid	Glucogallin	Isorhamnetin 3-O-galactoside	Salvianolic Acid A	Quercetin 3'-O-glucuronide	Genkwanin	Luteolin 7-O-diglucuronide_1	Luteolin 4'-glucoside	Dihydroferulic acid 4-O-

										glucuronide
14	Azadirachtin	Cafestol (2-hydroxy-)	Chrysoeriol	Melittoside	Chicoric acid	Theaflavin 3-O-gallate_1	Luteolin 7-O-diglucuronide_2	9-F1-phytoprostane	Dihydroferulic acid-4'-O-glucuronide
15	Ferulic acid-4'-O-glucoside	Rutin	Hesperidin	Eupatilin	Scutellarin	Chicoric acid	Citric acid	Quercetin 3,4'-O-diglucoside	9-F1-phytoprostane
16	Eupatilin	Theaflavin 3-O-gallate_1	Hesperetin	Acteoside	Silybin	Acteoside	Pectolinarigenin	Anhydrochiisanogenoic acid	Teupolioside
17	Kaempferol 3-O-sophoroside	Orientin	Citric acid	Genkwanin	Aucubin	Caffeoyl tartaric acid	Rhamnetin 3-glucoside	Cinnamtannin A2_2	Hexose
18	9-F1-phytoprostane	Chicoric acid	Eriocitrin_1	Caffeoyl tartaric acid	Apigenin-7-O-glucoside	9-F1-phytoprostane	kaempferol 3-O-rutinoside	Kaempferol 3-O-sophoroside	alpha-Methyl-D-mannopyranoside
19	Barbatoside C/D	Kaempferol 3-O-sophoroside	Astilbin	Chicoric acid	Oleuropein	Salvianolic acid B_2	Apigenin-7-O-glucoside	5-O-Caffeoylshikimic acid	Genistein 4',7-O-diglucuronide
20	Limonin	Scutellarin	Isorhamnetin-3-O-rutinoside	Hesperidin	Pinoresinol-4-O-Beta-Monoglycoside	Caffeic acid 4-O-glucoside	Pinoresinol-4-O-Beta-Monoglycoside	Myricetin 7-glucoside	6''-O-Malonylgenistin
21	Isorhamnetin-3-O-rutinoside	Eupatorin	Hispidulin glucuronide	Luteolin 7-O-diglucuronide_1	Caffeoyl tartaric acid	Isorhamnetin 3-O-galactoside	Nicotiflorin	Quercetin 3'-O-glucuronide	

22	Quercetin 3-arabinoside	Homoplantaginin_Tectoridin	Orientin	Cynarin	Lithospermic acid_1	Naringenin-4',5-diglucuronide	3,4-Dicaffeoylquinic Acid	Dihydroferulic acid-4'-O-glucuronide	
23	Bergenin	Luteolin 7-O-diglucuronide_1	kaempferol 3-O-rutinoside	kaempferol 3-O-rutinoside	alpha-Methyl-D-mannopyranoside	Kaempferol 3-O-(6''-acetyl-galactoside) 7-O-rhamnoside	Isoscutellarein 4'-methyl ether 7-(6'''-acetylallosyl)(1->2)-glucoside	Dihydroferulic acid 4-O-glucuronide	
24	Cirsilineol_1	Juglanin	Pinoresinol-4-O-Beta-Monoglycoside	Scutellarin	9-F1-phytoprostane	Dihydroferulic acid-4'-O-glucuronide	Isoscutellarein 7-O-[6'''-O-acetyl-β-d-allopyranosyl-(1→2)]-β-d-glucopyranoside	Coumaroylquinic acid	
25	Eriocitrin_2	Isorhamnetin 3-O-glucoside	Nicotiflorin	Salvianolic acid B_1	Dihydroferulic acid 4-O-glucuronide	6''-O-Malonyldaidzin	Apigenin 7-(4''-E-p-coumarylglucoside)	Salvianolic acid G	
26	Spinacetin 3-O-glucosyl-(1->6)-glucoside	Pectolinarigenin	Diosmin	Nicotiflorin	Naringenin-4',5-diglucuronide	Luteolin 7-O-diglucuronide_1	Cirsilineol_2	Myricetin-3-O-galactopyranoside	

27	Quercetin 3-O-(6''-acetyl-galactoside) 7-O-rhamnoside	Cynarin	Isoscutellarein 7-O-[6'''-O-acetyl-β-d-allopyranosyl-(1→2)]-β-d-glucopyranoside	3,4-Dicaffeoylquinic Acid	4-Hydroxy-5-(3',5'-dihydroxyphenyl)-valeric acid-O-glucuronide	Echinacoside	Isoscutellarein 7-O-[6'-O-acetyl-β-D-allopyranosyl-(1→2)]-6''-O-acetyl-β-D-glucopyranoside''	Kaempferol-3-o-glucuronide	
28	Isorhamnetin 3-O-galactoside	Aucubin	6''-O-Malonyldaidzin	Diosmin	Isorhamnetin 3-O-galactoside	Silydianin	Genistein 4',7-O-diglucuronide	Hyperoside	
29	5,5'-Dicaffeic acid	Geniposidic-Acid	Isorhamnetin 3-O-glucoside 7-O-rhamnoside	Luteolin-3-O-glucuronide	Hispidulin glucuronide	Achillolide A	Luteolin 4'-glucoside	Phloridzin	
30	Manghaslin (Quercetin 3-2G-rhamnosylrutinoside)	Pinoresinol-4-O-Beta-Monoglycoside	Salvianolic acid G	Isorhamnetin 3-O-glucoside 7-O-rhamnoside	Salvianolic acid C	Sucrose	4-Hydroxy-5-(3',5'-dihydroxyphenyl)-valeric acid-O-glucuronide	Caffeic acid	
31	Isoscutellarein 4'-methyl ether 7-(6'''-acetylallosyl)(1->2)-glucoside	Astilbin	Chrysoeriol 7-O-apiosyl-glucoside	Chrysoeriol 7-O-apiosyl-glucoside	5-Feruloylquinic acid	Manghaslin (Quercetin 3-2G-rhamnosylrutinoside)	Falcarindiol 3-acetate	Myricetin-3-O-α-L-rhamnopyranoside	

32	Quercetin-3-O-glucoside	Genistein 4',7-O-diglucuronide	Ferulic acid-4'-O-glucoside	Ferulic acid-4'-O-glucoside	Astragalin	Luteolin 7-O-glucoside	Antoside	Betonicine	
33	Allobetonicoside	Lithospermic acid_1	4-Hydroxy-5-(3',5'-dihydroxyphenyl)-valeric acid-O-glucuronide	Antoside	5-O-Caffeoylquinic acid	Silybin	Apigenin 7-O-apiosyl-glucoside	Pinoresinol-4-O-Beta-Monoglycoside	
34	Feruloyl C1-glucuronide	9-F1-phytoprostane	Naringenin-4',5-diglucuronide	Naringenin-4',5-diglucuronide	Luteolin 7-O-diglucuronide_1	Glucobrassicanapin	Kaempferol 3-O-acetyl-glucoside	Quercetin 3-glucuronate	
35	Neodiosmin	Chrysoeriol 7-O-apiosyl-glucoside	6''-O-Malonylgenistin	Kaempferol 3-rhamnosyl-(1->2)-rhamnosyl-(1->6)-glucoside	Cynarin	Luteolin-3-O-glucuronide	Oleuropein	Naringenin 5-O-glucuronide	
36	Glucogallin	Isorhamnetin 3-O-galactoside	Antoside	6''-O-Malonyldaidzin	Acteoside	Hexose	Isoacteoside	Quercetin 3-arabinoside	
37	Apigenin-7-O-glucoside	4-Hydroxy-5-(3',5'-dihydroxyphenyl)-valeric acid-O-glucuronide	Isorhamnetin 4'-O-glucuronide	Salvianolic acid G	Sucrose	Luteolin 4'-glucoside	1,3-Dicaffeoylquinic acid	Hexose	
38	6''-O-Malonyldaidzin	Naringenin-4',5-diglucuronide	Kaempferol 3-O-(6''-acetyl-galactoside) 7-O-rhamnoside	Quercetin 3-arabinoside	Hexose	Lithospermic acid_1	Apigenin 7-(2'',3''-diacetyl-hexocide)	Chrysoeriol 7-O-apiosyl-glucoside	

39	Caffeoyl tartaric acid	Kaempferol 3-rhamnosyl-(1->2)-rhamnosyl-(1->6)-glucoside	Homoplantaginin _Tectoridin	Manghaslin (Quercetin 3-2G-rhamnosylrutinoside)	Luteolin 4'-glucoside	Hydroxytyrosol 4-O-glucoside	alpha-Methyl-D-mannopyranoside	Luteolin-3-O-glucuronide	
40	Naringenin-4',5-diglucuronide	Harpagide	Apigenin 7-O-apiosyl-glucoside	Rhoifolin	Kaempferol 3-rhamnosyl-(1->2)-rhamnosyl-(1->6)-glucoside				
41	Luteolin 4'-glucoside	5-Feruloylquinic acid	Quercetin 3-O-(6-malonyl-glucoside)"	Luteolin 7-O-rutinoside	beta-D-Glcp-(1->4)-[L-alpha-D-Hepp-(1->3)]-L-alpha-D-Hepp				
42	Apigenin 7-O-apiosyl-glucoside	Plumieride	Quercetin 3-O-beta-D-glucopyranosyl-7-O-alpha-L-rhamnopyranoside	9-F1-phytoprostane	Isoscutellarein 4'-methyl ether 7-(6'''-acetylallosyl)(1->2)-glucoside				
43	Patulitrin	Hexose	Nobiletin	Sucrose					
44	Kaempferol 3-O-(6''-acetyl-	Sucrose	1,3-Dicaffeoylquinic acid	Hexose					

	galactoside) 7-O-rhamnoside								
45	Luteolin 7-O-diglucuronide_1	Silybin	4-O-Caffeoylquinic acid	5-O-Caffeoylshikimic acid					
46	Glucoalyssin	Acteoside	Byakangelicin	Lithospermic acid_1					
47	Tragopogonic acid	Lithospermic acid	Myricetin-3-O-α-L-rhamnopyranoside	Quercetin 3-rutinoside-7-glucoside					
48	Citric acid	Apigenin 7-O-diglucuronide	Cirsimaritin						
49	Myricetin 3-alpha-L-arabinopyranoside	Hispidulin glucuronide	Peonidin 3-O-sophoroside						
50	Echinacoside	Hydroxytyrosol 4-O-glucoside	Coumaroyl tartaric acid (p-)						
51	Cirsimaritin	beta-D-Glcp-(1->4)-[L-alpha-D-Hepp-(1->3)]-L-alpha-D-Hepp	Spinacetin 3-O-glucosyl-(1->6)-glucoside						
52	Hexose								
53	Leucosceptoside A								
54	Luteolin 7-O-rutinoside								
55	Sucrose								

56	5,7-Dihydroxychromone								
57	Nobiletin								
58	Morroniside								
59	Quercetin 3-rutinoside-7-glucoside								
60	Peonidin 3-O-sophoroside								
61	Betonicine								
62	Pelargonidin 3-O-rutinoside								
63	Chrysoeriol 7-O-apiosyl-glucoside								
64	6''-O-Acetylgenistin								
65	5-(3'-hydroxyphenyl)-gamma-hydroxyvaleric acid -4'-O-glucuronide								

Table S21. Phytochemicals of bitter orange with antioxidant activity according to international literature.

Sr. No.	Component name	Formatted citation
1	1,2-Disinapoylgentiobiose	[1]
2	Rhoifolin	[2]
3	Eriocitrin_1	[3]
4	Orientin	[4]
5	Kaempferol 3-O-acetyl-glucoside	[5]
6	D-(+)-Mannose	[6]
7	Nicotiflorin	[7]
8	Didymin	[8]
9	5-Feruloylquinic acid	[9]
10	Rutin	[10]
11	3,4-Dicaffeoylquinic Acid	[11]
12	Azadirachtin	[12]
13	Ferulic acid-4'-O-glucoside	[13]
14	Eupatilin	[14]
15	Kaempferol 3-O-sophoroside	[15]
16	Limonin	[16]
17	Isorhamnetin-3-O-rutinoside	[17]
18	Bergenin	[18]
19	Cirsilineol_1	[19]
20	Eriocitrin_2	[20]
21	Spinacetin 3-O-glucosyl-(1->6)-glucoside	[21]
22	Isorhamnetin 3-O-galactoside	[22]
23	Manghaslin (Quercetin 3-2G-rhamnosylrutinoside)	[23]

24	Quercetin-3-O-glucoside	[24]
25	Allobetonicoside	[25]
26	Feruloyl C1-glucuronide	[26]
27	Neodiosmin	[27]
28	Glucogallin	[28]
29	Apigenin-7-O-glucoside	[29]
30	Patulitrin	[30]
31	Luteolin 7-O-diglucuronide_1	[31]
32	Glucoalyssin	[32]
33	Citric acid	[33]
34	Myricetin 3-alpha-L-arabinopyranoside	[34]
35	Nobiletin	[36]
36	Sucrose	[39]
37	Luteolin 7-O-rutinoside	[40]
38	Hexose	[41]
39	Cirsimaritin	[46]
40	5,7-Dihydroxychromone	[48]
41	Morroniside	[49]
42	Echinacoside	[50]
43	Leucosceptoside A	[51]
44	Quercetin 3-rutinoside-7-glucoside	[53]

Table S22. Phytochemicals of dittany with antioxidant activity according to international literature.

Sr. No.	Component Name	Formatted Citation
1	Salvianolic acid B_1	[54]
2	Luteolin 7-O-diglucuronide_1	[55]
3	Rosmarinic acid	[56]
4	ferulic acid-4'-O-glucoside	[57]
5	Cirsilineol_1	[58]
6	Scutellarin	[59]
7	Salvianolic acid C	[60]
8	Pectolinarigenin	[61]
9	Orientin	[38]
10	Eupatorin	[62]
11	Vanillylmandelic acid	[63]
12	Cynarin	[64]
13	Rutin	[52]
14	Theaflavin 3-O-gallate_1	[65]
15	Isorhamnetin 3-O-glucoside	[66]
16	Juglanin	[67]
17	3,4-Dicaffeoylquinic Acid	[68]
18	Cafestol (2-hydroxy-)	[69]
19	Astilbin	[71]
20	Kaempferol 3-O-sophoroside	[44]
21	Pinoresinol-4-O-Beta-Monoglycoside	[72]
22	Glucogallin	[73]

23	6''-O-Malonylgenistin	[74]
24	Geniposidic-Acid	[75]
25	Isoacteoside	[76]
26	Homoplantaginin_Tectoridin	[77]
27	Naringenin 7-O-glucoside	[78]
28	Diosmin	[79]
29	Chicoric acid	[80]
30	Aucubin	[81]
31	Sucrose	[39]
32	Hexose	[82]
33	Lithospermic acid B	[57]
34	Acteoside	[83]
35	Silybin	[84]
36	Plumieride	[85]
37	5-Feruloylquinic acid	[86]

Table S23. Phytochemicals of lemon peel with antioxidant activity according to international literature.

Sr. No.	Component name	Formatted Citation
1	Eriocitrin_1	[45]
2	Nicotiflorin	[69]
3	Hesperidin	[108]
4	Rutin	[52]

5	D-(+)-Mannose	[90]
6	Diosmin	[79]
7	Chrysoeriol	[42]
8	Limonin	[38]
9	Orientin	[38]
10	Azadirachtin	[109]
11	Isorhamnetin-3-O-rutinoside	[66]
12	Hesperetin	[108]
13	Isorhamnetin 3-O-galactoside	[110]
14	Astilbin	[111]
15	Barbatoside A/B	[112]
16	Allobetonicoside	[113]
17	kaempferol 3-O-rutinoside	[114]
18	Quercetin-3-O-glucoside	[115]
19	Isorhamnetin 3-O-glucoside	[116]
20	Pinoresinol-4-O-Beta-Monoglycoside	[75]
21	Citric acid	[117]
22	Hispidulin glucuronide	[118]
23	Rosmarinic acid	[56]
24	Peonidin 3-O-sophoroside	[119]
25	Nobiletin	[36]
26	1,3-Dicaffeoylquinic acid	[100]
27	Myricetin-3-O-α-L-rhamnopyranoside	[120]
28	Quercetin 3-O-beta-D-glucopyranosyl-7-O-alpha-L-rhamnopyranoside	[102]
29	Quercetin 3-O-(6-malonyl-glucoside)"	[121]

30	4-O-Caffeoylquinic acid	[122]
31	Cirsimaritin	[123]

Table S24. Phytochemicals of spearmint with antioxidant activity according to international literature.

Sr. No.	Component name	Formatted Citation
1	Nicotiflorin	[69]
2	Scutellarin	[59]
3	Rosmarinic acid	[56]
4	Diosmin	[79]
5	Luteolin	[88]
6	Hesperidin	[108]
7	Vanillylmandelic acid	[63]
8	Caffeoyl tartaric acid	[137]
9	Salvianolic acid B_1	[54]
10	Salvianolic Acid A	[138]
11	Apigenin-7-O-glucuronide	[130]
12	Lithospermic acid B	[139]
13	Rutin	[52]
14	Luteolin 7-O-diglucuronide_1	[55]
15	Luteolin 7-O-glucoside	[87]
16	Eupatilin	[43]
17	Chicoric acid	[80]
18	Genkwanin	[140]
19	kaempferol 3-O-rutinoside	[114]

20	Isorhamnetin 3-O-glucoside	[116]
21	Cynarin	[64]
22	1,3-Dicaffeoylquinic acid	[100]
23	3,4-Dicaffeoylquinic Acid	[68]
24	Apigenin 7-O-diglucuronide	[130]
25	Oleuropein	[96]
26	Allobetonicoside	[113]
27	Acteoside	[132]
28	Melittoside	[129]
29	Rhoifolin	[141]
30	Manghaslin (Quercetin 3-2G-rhamnosylrutinoside)	[105]

Table S25. Phytochemicals of levander with antioxidant activity according to international literature.

.Sr. No.	Component name	Formatted Citation
1	Luteolin 7-O-diglucuronide_2	[87]
2	Scutellarin	[59]
3	Salvianolic acid B_2	[54]
4	Rosmarinic acid	[87]
5	Luteolin	[88]
6	ferulic acid-4'-O-glucoside	[57]
7	Apigenin-7-O-glucoside	[89]
8	D-(+)-Mannose	[90]

9	Apigenin	[91]
10	Pinoresinol-4-O-Beta-Monoglycoside	[72]
11	Quercitrin (Quercetin-3-O-alpha-L-rhamnopyranoside)	[92]
12	Luteolin-3-O-glucuronide	[93]
13	Quercetin 3'-O-glucuronide	[94]
14	Chicoric acid	[80]
15	Melittoside	[95]
16	Oleuropein	[96]
17	Theaflavin 3-O-gallate_1	[65]
18	Aucubin	[81]
19	Silybin	[84]
20	Luteolin 7-O-diglucuronide_1	[55]
21	Cynarin	[64]
22	Astragalin	[69]
23	Acteoside	[83]
24	Salvianolic acid C	[60]
25	5-Feruloylquinic acid	[86]
26	5-O-Caffeoylquinic acid	[97]

Table S26. Phytochemicals of lemon balm with antioxidant activity according to international literature.

Sr. No.	Component name	Formatted citation
1	Rosmarinic acid	[56]

2	Chicoric acid	[80]
3	Genkwanin	[98]).
4	Salvianolic Acid A	[99]
5	1,3-Dicaffeoylquinic acid	[100]
6	Quercitrin (Quercetin-3-O-alpha-L-rhamnopyranoside)	[92]
7	Quercetin 3-O-beta-D-glucopyranosyl-7-O-alpha-L-rhamnopyranoside	[102]
8	Diosmetin	[79]
9	Sucrose	[39]
10	Luteolin	[88]
11	Luteolin 7-O-glucoside	[103]
12	Salvianolic acid B_1	[54]
13	Theaflavin 3-O-gallate_1	[65]
14	Nicotiflorin	[101]
15	Lithospermic acid B	[57]
16	Luteolin 7-O-diglucuronide_1	[55]
17	Acteoside	[83]
18	Leucosceptoside A	[51]
19	Silydianin	[104]
20	Manghaslin (Quercetin 3-2G-rhamnosylrutinoside)	[105]
21	Echinacoside	[50]
22	Vanillylmandelic acid	[63]
23	Achillolide A	[106]
24	Silybin	[84]
25	Glucobrassicanapin	[107]

Table S27. Phytochemicals of sideritis with antioxidant activity according to international literature.

Sr. No.	Component name	Formatted Citation
1	Acteoside	[132]
2	Apigenin-7-O-glucoside	[89]
3	Teupolioside	[131]
4	Bergenin	[133]
5	Apigenin	[91]
6	Cirsilineol_1	[134]
7	Kaempferol 3-O-sophoroside	[15]
8	Leucosceptoside A	[51]
9	5-Feruloylquinic acid	[97]
10	kaempferol 3-O-rutinoside	[114]
11	Pectolinarigenin	[61]
12	Pinoresinol-4-O-Beta-Monoglycoside	[72]
13	Geniposidic-Acid	[75]
14	Luteolin 7-O-diglucuronide_2	[55]
15	Hesperidin	[108]
16	Nicotiflorin	[69]
17	Rhamnetin 3-glucoside	[135]
18	3,4-Dicaffeoylquinic Acid	[68]
19	Lithospermic acid B	[57]
20	Citric acid	[117]
21	Isoacteoside	[76]

22	1,3-Dicaffeoylquinic acid	[100]
23	Oleuropein	[96]
24	Luteolin 7-O-diglucuronide_1	[55]

Table S28. Phytochemicals of St. John's wort with antioxidant activity according to international literature.

Sr. No	Component name	Formatted Citation
1	Quercetin-3-O-glucoside	[115]
2	Astragalin	[143]
3	Kaempferol	[145]
4	Apigenin-7-O-glucuronide	[130]
5	Chicoric acid	[80]
6	Allobetonicoside	[113]
7	Apigenin-7-O-glucoside	[89]
8	Quercetin	[137]
9	Hyperoside	[146]
10	Myricetin-3-O-α-L-rhamnopyranoside	[120]
11	Amentoflavone	[144]
12	Cinnamtannin A2_1	[142]
13	Kaempferol-3-o-glucuronide	[147]
14	Rutin	[52]
15	Myricetin-3-O-galactopyranoside	[148]
16	Oleuropein	[96]
17	Pinoresinol-4-O-Beta-Monoglycoside	[72]
18	Caffeic acid	[149]

19	Betonicine	[150]
20	Phloridzin	[151]

Table S29. Phytochemicals of rosehip with antioxidant activity according to international literature.

Sr. No.	Component name	Formatted Citaion
1	Astilbin	[111]
2	Valoneic acid dilactone	[124]
3	5-O-Galloylquinic acid	[125]
4	Protocatechuic acid 4-O-glucoside	[126]
5	Nicotiflorin	[69]
6	Taxifolin	[127]
7	Ascorbic acid (L-)	[128]
8	Sucrose	[39]
9	Melittoside	[129]
10	Diosmin	[79]
11	Apigenin 7-O-diglucuronide	[130]
12	Hexose	[124]

Table S30. Polyphenolic components of each studied plant.

Polyphenolic Components									
**Sr. No**	**Bitter Orange**	**Dittany**	**Lemon Peel**	**Spearmint**	**St. John's wort**	**Lemon Balm**	**Lavender**	**Sideritis**	**Rosehip**
1	1,2-Disinapoylgentiobiose	3,4-Dicaffeoylquinic Acid	Quercetin-3-O-glucoside	Luteolin	Quercetin-3-O-glucoside	Rosmarinic acid	Rosmarinic acid	Cirsilineol_1	Apigenin 7-O-diglucuronide
2	Isoscutellarein 7-O-[6‴-O-acetyl-β-d-allopyranosyl-(1→2)]-β-d-glucopyranoside	Rosmarinic acid	Luteolin 7-O-glucoside	1,3-Dicaffeoylquinic acid	Astragalin	Lithospermic acid B	Luteolin-3-O-glucuronide	Bergenin	Valoneic acid dilactone
3	Rhoifolin	Salvianolic acid C	Rutin	Luteolin 7-O-glucoside	Rutin	Vanillylmandelic acid	Vanillylmandelic acid	Leucosceptoside A	5-O-Galloylquinic acid
4	Eriocitrin_1	Lithospermic acid B	Rosmarinic acid	Vanillylmandelic acid	Amentoflavone	Diosmetin	Apigenin	Lithospermic acid B	Astilbin
5	Orientin	Vanillylmandelic acid	Isorhamnetin 3-O-glucoside	Rosmarinic acid	Apigenin-7-O-glucuronide	Salvianolic acid B_1	Ferulic acid-4'-O-glucoside	5-Feruloylquinic acid	Taxifolin
6	Salvianolic acid G	Ferulic acid-4'-O-glucoside	Isorhamnetin 3-O-galactoside	Lithospermic acid B	Kaempferol	Nicotiflorin	Salvianolic acid B_2	Apigenin	Protocatechuic acid 4-O-glucoside

7	Kaempferol 3-O-acetyl-glucoside	Diosmin	Chrysoeriol	Isorhamnetin 3-O-glucoside	Cinnamtannin A2_1	1,3-Dicaffeoylquinic acid	Luteolin 7-O-diglucuronide_2	Kaempferol 3-O-sophoroside	Nicotiflorin
8	Nicotiflorin	Salvianolic acid B_1	Hesperidin	Apigenin-7-O-glucuronide	Quercetin	Leucosceptoside A	Luteolin	Acteoside	Diosmin
9	Didymin	Isoacteoside	Hesperetin	Apigenin 7-O-diglucuronide	Chicoric acid	Salvianolic Acid A	Theaflavin 3-O-gallate_1	Hesperidin	Quercetin 3-arabinoside
10	5-Feruloylquinic acid	6''-O-Malonylgenistin	Eriocitrin_1	Salvianolic Acid A	Apigenin-7-O-glucoside	Luteolin	Quercetin 3'-O-glucuronide	Luteolin 7-O-diglucuronide_1	Dihydroferulic acid 4-O-glucuronide
11	Rutin	Naringenin 7-O-glucoside	Astilbin	Eupatilin	Luteolin 4'-glucoside	Genkwanin	Chicoric acid	Luteolin 7-O-diglucuronide_2	Dihydroferulic acid-4'-O-glucuronide
12	3,4-Dicaffeoylquinic Acid	Cirsilineol_1	Isorhamnetin-3-O-rutinoside	Genkwanin	Quercetin 3,4'-O-diglucoside	Theaflavin 3-O-gallate_1	Scutellarin	Pectolinarigenin	Genistein 4',7-O-diglucuronide
13	Ferulic acid-4'-O-glucoside	Glucogallin	Hispidulin glucuronide	Caffeoyl tartaric acid	Cinnamtannin A2_2	Chicoric acid	Silybin	Rhamnetin 3-glucoside	6''-O-Malonylgenistin

14	Eupatilin	Cafestol (2-hydroxy-)	Orientin	Chicoric acid	Kaempferol 3-O-sophoroside	Acteoside	Apigenin-7-O-glucoside	kaempferol 3-O-rutinoside	
15	Kaempferol 3-O-sophoroside	Rutin	kaempferol 3-O-rutinoside	Hesperidin	5-O-Caffeoylshikimic acid	Caffeoyl tartaric acid	Caffeoyl tartaric acid	Apigenin-7-O-glucoside	
16	Barbatoside C/D	Theaflavin 3-O-gallate_1	Nicotiflorin	Luteolin 7-O-diglucuronide_1	Myricetin 7-glucoside	Salvianolic acid B_2	Lithospermic acid_1	Nicotiflorin	
17	Isorhamnetin-3-O-rutinoside	Orientin	Diosmin	Cynarin	Quercetin 3'-O-glucuronide	Caffeic acid 4-O-glucoside	Dihydroferulic acid 4-O-glucuronide	3,4-Dicaffeoylquinic Acid	
18	Quercetin 3-arabinoside	Chicoric acid	Isoscutellarein 7-O-[6‴-O-acetyl-β-d-allopyranosyl-(1→2)]-β-d-glucopyranoside	kaempferol 3-O-rutinoside	Dihydroferulic acid-4'-O-glucuronide	Isorhamnetin 3-O-galactoside	Naringenin-4',5-diglucuronide	Isoscutellarein 7-O-[6‴-O-acetyl-β-d-allopyranosyl-(1→2)]-β-d-glucopyranoside	
19	Bergenin	Kaempferol 3-O-sophoroside	6''-O-Malonyldaidzin	Scutellarin	Dihydroferulic acid 4-O-glucuronide	Naringenin-4',5-diglucuronide	Isorhamnetin 3-O-galactoside	Cirsilineol_2	
20	Cirsilineol_1	Scutellarin	Isorhamnetin 3-O-glucoside 7-O-rhamnoside	Salvianolic acid B_1	Coumaroylquinic acid	Kaempferol 3-O-(6''-acetyl-galactoside) 7-	Hispidulin glucuronide	Isoscutellarein 7-O-[6'-O-acetyl-β-D-	

						O-rhamnoside		allopyranosyl-(1→2)]-6''-O-acetyl-β-D-glucopyranoside''	
21	Eriocitrin_2	Eupatorin	Salvianolic acid G	Nicotiflorin	Salvianolic acid G	Dihydroferulic acid-4'-O-glucuronide	Salvianolic acid C	Genistein 4',7-O-diglucuronide	
22	Spinacetin 3-O-glucosyl-(1->6)-glucoside	Homoplantaginin_Tectoridin	Chrysoeriol 7-O-apiosyl-glucoside	3,4-Dicaffeoylquinic Acid	Myricetin-3-O-galactopyranoside	6''-O-Malonyldaidzin	5-Feruloylquinic acid	Luteolin 4'-glucoside	
23	Quercetin 3-O-(6''-acetyl-galactoside) 7-O-rhamnoside	Luteolin 7-O-diglucuronide_1	Ferulic acid-4'-O-glucoside	Diosmin	Kaempferol-3-o-glucuronide	Luteolin 7-O-diglucuronide _1	Astragalin	Antoside	
24	Isorhamnetin 3-O-galactoside	Juglanin	Naringenin-4',5-diglucuronide	Luteolin-3-O-glucuronide	Hyperoside	Silydianin	5-O-Caffeoylquinic acid	Apigenin 7-O-apiosyl-glucoside	
25	5,5'-Dicaffeic acid	Isorhamnetin 3-O-glucoside	6''-O-Malonylgenistin	Isorhamnetin 3-O-glucoside 7-O-rhamnoside	Phloridzin	Manghaslin (Quercetin 3-2G-rhamnosylrutinoside)	Luteolin 7-O-diglucuronide_1	Kaempferol 3-O-acetyl-glucoside	
26	Manghaslin (Quercetin 3-2G-	Pectolinarigenin	Antoside	Chrysoeriol 7-O-apiosyl-glucoside	Caffeic acid	Luteolin 7-O-glucoside	Cynarin	Isoacteoside	

	rhamnosylrutinoside)								
27	Quercetin-3-O-glucoside	Cynarin	Isorhamnetin 4'-O-glucuronide	Ferulic acid-4'-O-glucoside	Myricetin-3-O-α-L-rhamnopyranoside	Silybin	Acteoside	1,3-Dicaffeoylquinic acid	
28	Feruloyl C1-glucuronide	Astilbin	Kaempferol 3-O-(6''-acetyl-galactoside) 7-O-rhamnoside	Antoside	Quercetin 3-glucuronate	Luteolin-3-O-glucuronide	Luteolin 4'-glucoside		
29	Neodiosmin	Genistein 4',7-O-diglucuronide	Homoplantaginin_Tectoridin	Naringenin-4',5-diglucuronide	Naringenin 5-O-glucuronide	Luteolin 4'-glucoside	Kaempferol 3-rhamnosyl-(1->2)-rhamnosyl-(1->6)-glucoside		
30	Glucogallin	Lithospermic acid_1	Apigenin 7-O-apiosyl-glucoside	Kaempferol 3-rhamnosyl-(1->2)-rhamnosyl-(1->6)-glucoside	Quercetin 3-arabinoside	Lithospermic acid_1			
31	Apigenin-7-O-glucoside	Chrysoeriol 7-O-apiosyl-glucoside	Quercetin 3-O-(6-malonyl-glucoside)"	6''-O-Malonyldaidzin	Chrysoeriol 7-O-apiosyl-glucoside	Hydroxytyrosol 4-O-glucoside			
32	6''-O-Malonyldaidzin	Isorhamnetin 3-O-galactoside	Nobiletin	Salvianolic acid G	Luteolin-3-O-glucuronide				

33	Caffeoyl tartaric acid	Naringenin-4',5-diglucuronide	1,3-Dicaffeoylquinic acid	Quercetin 3-arabinoside					
34	Naringenin-4',5-diglucuronide	Kaempferol 3-rhamnosyl-(1->2)-rhamnosyl-(1->6)-glucoside	4-O-Caffeoylquinic acid	Manghaslin (Quercetin 3-2G-rhamnosylrutinoside)					
35	Luteolin 4'-glucoside	5-Feruloylquinic acid	Byakangelicin	Rhoifolin					
36	Apigenin 7-O-apiosyl-glucoside	Silybin	Myricetin-3-O-α-L-rhamnopyranoside	Luteolin 7-O-rutinoside					
37	Patulitrin	Acteoside	Cirsimaritin	5-O-Caffeoylshikimic acid					
38	Kaempferol 3-O-(6''-acetyl-galactoside) 7-O-rhamnoside	Lithospermic acid	Peonidin 3-O-sophoroside	Lithospermic acid_1					
39	Luteolin 7-O-diglucuronide _1	Apigenin 7-O-diglucuronide	Coumaroyl tartaric acid (p-)	Quercetin 3-rutinoside-7-glucoside					
40	Myricetin 3-alpha-L-arabinopyranoside	Hispidulin glucuronide	Spinacetin 3-O-glucosyl-(1->6)-glucoside						
41	Cirsimaritin	Hydroxytyrosol 4-O-glucoside							

42	Leucosceptoside A								
43	Luteolin 7-O-rutinoside								
44	5,7-Dihydroxychromone								
45	Nobiletin								
46	Morroniside								
47	Quercetin 3-rutinoside-7-glucoside								
48	Peonidin 3-O-sophoroside								
49	Pelargonidin 3-O-rutinoside								
50	Chrysoeriol 7-O-apiosyl-glucoside								
51	6''-O-Acetylgenistin								
52	5-(3'-hydroxyphenyl)-gamma-hydroxyvaleric acid -4'-O-glucuronide								

References:

[1] X. L. PIAO, H. Y. KIM, T. YOKOZAWA, Y. A. LEE, X. S. PIAO, and E. J. CHO, "Protective Effects of Broccoli (Brassica oleracea) and Its Active Components against Radical-Induced Oxidative Damage," *J Nutr Sci Vitaminol (Tokyo)*, vol. 51, no. 3, pp. 142–147, 2005, doi: 10.3177/jnsv.51.142.

[2] B. Zou, T. Li, Y. Xu, Y. Yu, and J. Wu, "Structural identification and antioxidant potency evaluation of pomelo vinegar polyphenols," *Food Biosci*, vol. 47, p. 101674, Jun. 2022, doi: 10.1016/j.fbio.2022.101674.

[3] L. Yao, W. Liu, M. Bashir, M. F. Nisar, and C. (Craig) Wan, "Eriocitrin: A review of pharmacological effects," *Biomedicine & Pharmacotherapy*, vol. 154, p. 113563, Oct. 2022, doi: 10.1016/j.biopha.2022.113563.

[4] S. Nagai, C. Matsumoto, M. Shibano, and K. Fujimori, "Suppression of Fatty Acid and Triglyceride Synthesis by the Flavonoid Orientin through Decrease of C/EBPδ Expression and Inhibition of PI3K/Akt-FOXO1 Signaling in Adipocytes," *Nutrients*, vol. 10, no. 2, p. 130, Jan. 2018, doi: 10.3390/nu10020130.

[5] M. J. Larrazábal-Fuentes *et al.*, "Chemical Profiling, Antioxidant, Anticholinesterase, and Antiprotozoal Potentials of Artemisia copa Phil. (Asteraceae)," *Front Pharmacol*, vol. 11, Dec. 2020, doi: 10.3389/fphar.2020.594174.

[6] A. Hameed, N. Iqbal, and S. A. Malik, "Effect of d-mannose on antioxidant defense and oxidative processes in etiolated wheat coleoptiles," *Acta Physiol Plant*, vol. 36, no. 1, pp. 161–167, Jan. 2014, doi: 10.1007/s11738-013-1396-5.

[7] S. Bakir, S. Kamiloglu, M. Tomas, and E. Capanoglu, "Tomato Polyphenolics: Putative Applications to Health and Disease," in *Polyphenols: Mechanisms of Action in Human Health and Disease*, Elsevier, 2018, pp. 93–102. doi: 10.1016/B978-0-12-813006-3.00009-X.

[8] K. Shukla, H. Sonowal, A. Saxena, and K. v. Ramana, "Didymin by suppressing NF-κB activation prevents VEGF-induced angiogenesis in vitro and in vivo," *Vascul Pharmacol*, vol. 115, pp. 18–25, Apr. 2019, doi: 10.1016/j.vph.2019.01.002.

[9] X. Ao *et al.*, "Extraction, isolation and identification of four phenolic compounds from Pleioblastus amarus shoots and their antioxidant and anti-inflammatory properties in vitro," *Food Chem*, vol. 374, p. 131743, Apr. 2022, doi: 10.1016/j.foodchem.2021.131743.

[10] B. Lue, A. M. Sørensen, C. Jacobsen, Z. Guo, and X. Xu, "Antioxidant efficacies of rutin and rutin esters in bulk oil and oil-in-water emulsion," *European Journal of Lipid Science and Technology*, vol. 119, no. 4, p. 1600049, Apr. 2017, doi: 10.1002/ejlt.201600049.

[11] Y. J. Hyun *et al.*, “3,4-Dicaffeoylquinic acid protects human keratinocytes against environmental oxidative damage,” *J Funct Foods*, vol. 52, pp. 430–441, Jan. 2019, doi: 10.1016/j.jff.2018.11.026.

[12] A. K. Ghimeray, C.-W. Jin, B. K. Ghimire, and D. H. Cho, “Antioxidant activity and quantitative estimation of azadirachtin and nimbin in Azadirachta Indica A. Juss grown in foothills of Nepal,” *Afr J Biotechnol*, vol. 8, no. 13, pp. 3084–3091, 2009, [Online]. Available: http://www.academicjournals.org/AJB

[13] A. Piazzon, U. Vrhovsek, D. Masuero, F. Mattivi, F. Mandoj, and M. Nardini, “Antioxidant Activity of Phenolic Acids and Their Metabolites: Synthesis and Antioxidant Properties of the Sulfate Derivatives of Ferulic and Caffeic Acids and of the Acyl Glucuronide of Ferulic Acid,” *J Agric Food Chem*, vol. 60, no. 50, pp. 12312–12323, Dec. 2012, doi: 10.1021/jf304076z.

[14] A. Rosa, R. Isola, F. Pollastro, P. Caria, G. Appendino, and M. Nieddu, “The dietary flavonoid eupatilin attenuates *in vitro* lipid peroxidation and targets lipid profile in cancer HeLa cells,” *Food Funct*, vol. 11, no. 6, pp. 5179–5191, 2020, doi: 10.1039/D0FO00777C.

[15] G. W. Plumb, K. R. Price, M. J. C. Modes, and G. Williamson, “Antioxidant Properties of the Major Polyphenolic Compounds in Broccoli,” *Free Radic Res*, vol. 27, no. 4, pp. 429–435, Jan. 1997, doi: 10.3109/10715769709065782.

[16] Y. D. Singh, D. Das, S. Das, K. D. Swain, S. Pradhan, and P. J. Babu, “Pharmacological activities of limonin from Khasi Mandarin as therapeutic applications,” *Pharmacological Research - Modern Chinese Medicine*, vol. 5, p. 100181, Dec. 2022, doi: 10.1016/j.prmcm.2022.100181.

[17] M. Shibano, K. Kakutani, M. Taniguchi, M. Yasuda, and K. Baba, “Antioxidant constituents in the dayflower (Commelina communis L.) and their α-glucosidase-inhibitory activity,” *J Nat Med*, vol. 62, no. 3, pp. 349–353, Jul. 2008, doi: 10.1007/s11418-008-0244-1.

[18] H. A. de Abreu, I. Aparecida dos S. Lago, G. P. Souza, D. Piló-Veloso, H. A. Duarte, and A. F. de C. Alcântara, “Antioxidant activity of (+)-bergenin—a phytoconstituent isolated from the bark of Sacoglottis uchi Huber (Humireaceae),” *Org Biomol Chem*, vol. 6, no. 15, p. 2713, 2008, doi: 10.1039/b804385j.

[19] M. A. Kelm, M. G. Nair, G. M. Strasburg, and D. L. DeWitt, “Antioxidant and cyclooxygenase inhibitory phenolic compounds from Ocimum sanctum Linn.,” *Phytomedicine*, vol. 7, no. 1, pp. 7–13, Mar. 2000, doi: 10.1016/S0944-7113(00)80015-X.

[20] L. Yao, W. Liu, M. Bashir, M. F. Nisar, and C. (Craig) Wan, “Eriocitrin: A review of pharmacological effects,” *Biomedicine & Pharmacotherapy*, vol. 154, p. 113563, Oct. 2022, doi: 10.1016/j.biopha.2022.113563.

[21] Z. Kokanova-Nedialkova and P. T. Nedialkov, "Antioxidant properties of 6-methoxyflavonol glycosides from the aerial parts of Chenopodium bonus-henricus L ACTIVITY-GUIDED ANALYSIS OF THE ANTICANCER METABOLITES OF UNSTUDIED SPECIES OF THE BULGARIAN FLORA AND CERTIFIED FOREIGN HYBRIDS OF GENUS JUNIPERUS L. (CUPRESSACEAE) USING MODERN RESEARCH TECHNOLOGIES View project," 2017. [Online]. Available: https://www.researchgate.net/publication/324562545

[22] M. Shibano, K. Kakutani, M. Taniguchi, M. Yasuda, and K. Baba, "Antioxidant constituents in the dayflower (Commelina communis L.) and their α-glucosidase-inhibitory activity," *J Nat Med*, vol. 62, no. 3, pp. 349–353, Jul. 2008, doi: 10.1007/s11418-008-0244-1.

[23] A. Nugroho, H. Heryani, J. S. Choi, and H.-J. Park, "Identification and quantification of flavonoids in Carica papaya leaf and peroxynitrite-scavenging activity," *Asian Pac J Trop Biomed*, vol. 7, no. 3, pp. 208–213, Mar. 2017, doi: 10.1016/j.apjtb.2016.12.009.

[24] S. M. Razavi, S. Zahri, G. Zarrini, H. Nazemiyeh, and S. Mohammadi, "Biological activity of quercetin-3-O-glucoside, a known plant flavonoid," *Russ J Bioorg Chem*, vol. 35, no. 3, pp. 376–378, May 2009, doi: 10.1134/S1068162009030133.

[25] M. de la L. Cádiz-Gurrea, D. Pinto, C. Delerue-Matos, and F. Rodrigues, "Olive Fruit and Leaf Wastes as Bioactive Ingredients for Cosmetics—A Preliminary Study," *Antioxidants*, vol. 10, no. 2, p. 245, Feb. 2021, doi: 10.3390/antiox10020245.

[26] A. Piazzon, U. Vrhovsek, D. Masuero, F. Mattivi, F. Mandoj, and M. Nardini, "Antioxidant Activity of Phenolic Acids and Their Metabolites: Synthesis and Antioxidant Properties of the Sulfate Derivatives of Ferulic and Caffeic Acids and of the Acyl Glucuronide of Ferulic Acid," *J Agric Food Chem*, vol. 60, no. 50, pp. 12312–12323, Dec. 2012, doi: 10.1021/jf304076z.

[27] E. da Pozzo *et al.*, "Antioxidant and Antisenescence Effects of Bergamot Juice," *Oxid Med Cell Longev*, vol. 2018, pp. 1–14, Jul. 2018, doi: 10.1155/2018/9395804.

[28] A. N. Khan *et al.*, "A Short Review on Glucogallin and its Pharmacological Activities," *Mini-Reviews in Medicinal Chemistry*, vol. 22, no. 22, pp. 2820–2830, Dec. 2022, doi: 10.2174/1389557522666220513150907.

[29] W. Wang *et al.*, "Efficiency comparison of apigenin-7-O-glucoside and trolox in antioxidative stress and anti-inflammatory properties," *Journal of Pharmacy and Pharmacology*, vol. 72, no. 11, pp. 1645–1656, Oct. 2020, doi: 10.1111/jphp.13347.

[30] "Antioxidant Activity Of Patulitrin From Tagetes Patula," *Indian J Appl Res*.

[31] S. Rehecho *et al.*, “Chemical composition, mineral content and antioxidant activity of Verbena officinalis L.,” *LWT - Food Science and Technology*, vol. 44, no. 4, pp. 875–882, May 2011, doi: 10.1016/j.lwt.2010.11.035.

[32] M.-K. Lee *et al.*, “Variation of glucosinolates in 62 varieties of Chinese cabbage (Brassica rapa L. ssp. pekinensis) and their antioxidant activity,” *LWT - Food Science and Technology*, vol. 58, no. 1, pp. 93–101, Sep. 2014, doi: 10.1016/j.lwt.2014.03.001.

[33] E. M. Ryan *et al.*, “Antioxidant properties of citric acid interfere with the uricase-based measurement of circulating uric acid,” *J Pharm Biomed Anal*, vol. 164, pp. 460–466, Feb. 2019, doi: 10.1016/j.jpba.2018.11.011.

[34] H. Okamura, A. Mimura, Y. Yakou, M. Niwano, and Y. Takahara, “Antioxidant activity of tannins and flavonoids in Eucalyptus rostrata,” *Phytochemistry*, vol. 33, no. 3, pp. 557–561, Jun. 1993, doi: 10.1016/0031-9422(93)85448-Z.

[35] B. Zou, T. Li, Y. Xu, Y. Yu, and J. Wu, “Structural identification and antioxidant potency evaluation of pomelo vinegar polyphenols,” *Food Biosci*, vol. 47, p. 101674, Jun. 2022, doi: 10.1016/j.fbio.2022.101674.

[36] M. Wang *et al.*, “Antioxidant Protection of Nobiletin, 5-Demethylnobiletin, Tangeretin, and 5-Demethyltangeretin from Citrus Peel in *Saccharomyces cerevisiae*,” *J Agric Food Chem*, vol. 66, no. 12, pp. 3155–3160, Mar. 2018, doi: 10.1021/acs.jafc.8b00509.

[37] X. L. PIAO, H. Y. KIM, T. YOKOZAWA, Y. A. LEE, X. S. PIAO, and E. J. CHO, “Protective Effects of Broccoli (Brassica oleracea) and Its Active Components against Radical-Induced Oxidative Damage,” *J Nutr Sci Vitaminol (Tokyo)*, vol. 51, no. 3, pp. 142–147, 2005, doi: 10.3177/jnsv.51.142.

[38] S. Nagai, C. Matsumoto, M. Shibano, and K. Fujimori, “Suppression of Fatty Acid and Triglyceride Synthesis by the Flavonoid Orientin through Decrease of C/EBPδ Expression and Inhibition of PI3K/Akt-FOXO1 Signaling in Adipocytes,” *Nutrients*, vol. 10, no. 2, p. 130, Jan. 2018, doi: 10.3390/nu10020130.

[39] B. C. Q. Nguyen, M. Shahinozzaman, N. T. K. Tien, T. N. Thach, and S. Tawata, “Effect of sucrose on antioxidant activities and other health-related micronutrients in gamma-aminobutyric acid (GABA)-enriched sprouting Southern Vietnam brown rice,” *J Cereal Sci*, vol. 93, p. 102985, May 2020, doi: 10.1016/j.jcs.2020.102985.

[40] N. M. Kim, J. Kim, H. Y. Chung, and J. S. Choi, “Isolation of luteolin 7-O-rutinoside and esculetin with potential antioxidant activity from the aerial parts ofArtemisia montana,” *Arch Pharm Res*, vol. 23, no. 3, pp. 237–239, Jun. 2000, doi: 10.1007/BF02976451.

[41] C. Frezza *et al.*, "Leucosceptosides A and B: Two Phenyl-Ethanoid Glycosides with Important Occurrence and Biological Activities," *Biomolecules*, vol. 12, no. 12, p. 1807, Dec. 2022, doi: 10.3390/biom12121807.

[42] Y. D. Singh, D. Das, S. Das, K. D. Swain, S. Pradhan, and P. J. Babu, "Pharmacological activities of limonin from Khasi Mandarin as therapeutic applications," *Pharmacological Research - Modern Chinese Medicine*, vol. 5, p. 100181, Dec. 2022, doi: 10.1016/j.prmcm.2022.100181.

[43] A. Rosa, R. Isola, F. Pollastro, P. Caria, G. Appendino, and M. Nieddu, "The dietary flavonoid eupatilin attenuates *in vitro* lipid peroxidation and targets lipid profile in cancer HeLa cells," *Food Funct*, vol. 11, no. 6, pp. 5179–5191, 2020, doi: 10.1039/D0FO00777C.

[44] G. W. Plumb, K. R. Price, M. J. C. Modes, and G. Williamson, "Antioxidant Properties of the Major Polyphenolic Compounds in Broccoli," *Free Radic Res*, vol. 27, no. 4, pp. 429–435, Jan. 1997, doi: 10.3109/10715769709065782.

[45] L. Yao, W. Liu, M. Bashir, M. F. Nisar, and C. (Craig) Wan, "Eriocitrin: A review of pharmacological effects," *Biomedicine & Pharmacotherapy*, vol. 154, p. 113563, Oct. 2022, doi: 10.1016/j.biopha.2022.113563.

[46] T. Benali *et al.*, "The Current State of Knowledge in Biological Properties of Cirsimaritin," *Antioxidants*, vol. 11, no. 9, p. 1842, Sep. 2022, doi: 10.3390/antiox11091842.

[47] K. Shukla, H. Sonowal, A. Saxena, and K. v. Ramana, "Didymin by suppressing NF-κB activation prevents VEGF-induced angiogenesis in vitro and in vivo," *Vascul Pharmacol*, vol. 115, pp. 18–25, Apr. 2019, doi: 10.1016/j.vph.2019.01.002.

[48] J. Qiu *et al.*, "Screening natural antioxidants in peanut shell using DPPH–HPLC–DAD–TOF/MS methods," *Food Chem*, vol. 135, no. 4, pp. 2366–2371, Dec. 2012, doi: 10.1016/j.foodchem.2012.07.042.

[49] H. Xu, J. Shen, H. Liu, Y. Shi, L. Li, and M. Wei, "Morroniside and loganin extracted from Cornus officinalis have protective effects on rat mesangial cell proliferation exposed to advanced glycation end products by preventing oxidative stress," *Can J Physiol Pharmacol*, vol. 84, no. 12, pp. 1267–1273, Dec. 2006, doi: 10.1139/y06-075.

[50] B. D. Sloley, L. J. Urichuk, C. Tywin, R. T. Coutts, P. K. T. Pang, and J. J. Shan, "Comparison of chemical components and antioxidant capacity of different *Echinacea* species," *Journal of Pharmacy and Pharmacology*, vol. 53, no. 6, pp. 849–857, Feb. 2010, doi: 10.1211/0022357011776009.

[51] C. Frezza *et al.*, "Leucosceptosides A and B: Two Phenyl-Ethanoid Glycosides with Important Occurrence and Biological Activities," *Biomolecules*, vol. 12, no. 12, p. 1807, Dec. 2022, doi: 10.3390/biom12121807.

[52] B. Lue, A. M. Sørensen, C. Jacobsen, Z. Guo, and X. Xu, “Antioxidant efficacies of rutin and rutin esters in bulk oil and oil-in-water emulsion,” *European Journal of Lipid Science and Technology*, vol. 119, no. 4, p. 1600049, Apr. 2017, doi: 10.1002/ejlt.201600049.

[53] Y.-Z. Zheng, G. Deng, Q. Liang, D.-F. Chen, R. Guo, and R.-C. Lai, “Antioxidant Activity of Quercetin and Its Glucosides from Propolis: A Theoretical Study,” *Sci Rep*, vol. 7, no. 1, p. 7543, Aug. 2017, doi: 10.1038/s41598-017-08024-8.

[54] Z. Xiao *et al.*, “Pharmacological Effects of Salvianolic Acid B Against Oxidative Damage,” *Front Pharmacol*, vol. 11, Nov. 2020, doi: 10.3389/fphar.2020.572373.

[55] S. Rehecho *et al.*, “Chemical composition, mineral content and antioxidant activity of Verbena officinalis L.,” *LWT - Food Science and Technology*, vol. 44, no. 4, pp. 875–882, May 2011, doi: 10.1016/j.lwt.2010.11.035.

[56] A. G. Adomako-Bonsu, S. L. Chan, M. Pratten, and J. R. Fry, “Antioxidant activity of rosmarinic acid and its principal metabolites in chemical and cellular systems: Importance of physico-chemical characteristics,” *Toxicology in Vitro*, vol. 40, pp. 248–255, Apr. 2017, doi: 10.1016/j.tiv.2017.01.016.

[57] J. Damašius, P. R. Venskutonis, V. Kaškonienė, and A. Maruška, “Fast screening of the main phenolic acids with antioxidant properties in common spices using on-line HPLC/UV/DPPH radical scavenging assay,” *Analytical Methods*, vol. 6, no. 8, p. 2774, 2014, doi: 10.1039/c3ay41703d.

[58] M. A. Kelm, M. G. Nair, G. M. Strasburg, and D. L. DeWitt, “Antioxidant and cyclooxygenase inhibitory phenolic compounds from Ocimum sanctum Linn.,” *Phytomedicine*, vol. 7, no. 1, pp. 7–13, Mar. 2000, doi: 10.1016/S0944-7113(00)80015-X.

[59] M. Spiegel, T. Marino, M. Prejanò, and N. Russo, “Primary and secondary antioxidant properties of scutellarin and scutellarein in water and lipid-like environments: A theoretical investigation,” *J Mol Liq*, vol. 366, p. 120343, Nov. 2022, doi: 10.1016/j.molliq.2022.120343.

[60] L.-H. Chien, C.-T. Wu, J.-S. Deng, W.-P. Jiang, W.-C. Huang, and G.-J. Huang, “Salvianolic Acid C Protects against Cisplatin-Induced Acute Kidney Injury through Attenuation of Inflammation, Oxidative Stress and Apoptotic Effects and Activation of the CaMKK–AMPK–Sirt1-Associated Signaling Pathway in Mouse Models,” *Antioxidants*, vol. 10, no. 10, p. 1620, Oct. 2021, doi: 10.3390/antiox10101620.

[61] M. Shiraiwa, T. Kitakaze, Y. Yamashita, Y. Ukawa, K. Mukai, and H. Ashida, “Pectolinarigenin Induces Antioxidant Enzymes through Nrf2/ARE Pathway in HepG2 Cells,” *Antioxidants*, vol. 11, no. 4, p. 675, Mar. 2022, doi: 10.3390/antiox11040675.

[62] D. Procházková, I. Boušová, and N. Wilhelmová, “Antioxidant and prooxidant properties of flavonoids,” *Fitoterapia*, vol. 82, no. 4, pp. 513–523, Jun. 2011, doi: 10.1016/j.fitote.2011.01.018.

[63] D. Dimić *et al.*, "Experimental and theoretical elucidation of structural and antioxidant properties of vanillylmandelic acid and its carboxylate anion," *Spectrochim Acta A Mol Biomol Spectrosc*, vol. 198, pp. 61–70, Jun. 2018, doi: 10.1016/j.saa.2018.02.063.

[64] M. Topal *et al.*, "Antioxidant, antiradical, and anticholinergic properties of cynarin purified from the Illyrian thistle (*Onopordum illyricum* L.)," *J Enzyme Inhib Med Chem*, vol. 31, no. 2, pp. 266–275, Mar. 2016, doi: 10.3109/14756366.2015.1018244.

[65] N. J. Miller, C. Castelluccio, L. Tijburg, and C. Rice-Evans, "The antioxidant properties of theaflavins and their gallate esters - radical scavengers or metal chelators?," *FEBS Lett*, vol. 392, no. 1, pp. 40–44, Aug. 1996, doi: 10.1016/0014-5793(96)00780-6.

[66] M. Shibano, K. Kakutani, M. Taniguchi, M. Yasuda, and K. Baba, "Antioxidant constituents in the dayflower (Commelina communis L.) and their α-glucosidase-inhibitory activity," *J Nat Med*, vol. 62, no. 3, pp. 349–353, Jul. 2008, doi: 10.1007/s11418-008-0244-1.

[67] Y. Ren *et al.*, "Juglanin ameliorates depression-like behavior in chronic unpredictable mild stress-induced mice by improving AMPK signaling," *J Funct Foods*, vol. 98, p. 105263, Nov. 2022, doi: 10.1016/j.jff.2022.105263.

[68] Y. J. Hyun *et al.*, "3,4-Dicaffeoylquinic acid protects human keratinocytes against environmental oxidative damage," *J Funct Foods*, vol. 52, pp. 430–441, Jan. 2019, doi: 10.1016/j.jff.2018.11.026.

[69] K. J. Lee, J. H. Choi, and H. G. Jeong, "Hepatoprotective and antioxidant effects of the coffee diterpenes kahweol and cafestol on carbon tetrachloride-induced liver damage in mice," *Food and Chemical Toxicology*, vol. 45, no. 11, pp. 2118–2125, Nov. 2007, doi: 10.1016/j.fct.2007.05.010.

[70] A. Piazzon, U. Vrhovsek, D. Masuero, F. Mattivi, F. Mandoj, and M. Nardini, "Antioxidant Activity of Phenolic Acids and Their Metabolites: Synthesis and Antioxidant Properties of the Sulfate Derivatives of Ferulic and Caffeic Acids and of the Acyl Glucuronide of Ferulic Acid," *J Agric Food Chem*, vol. 60, no. 50, pp. 12312–12323, Dec. 2012, doi: 10.1021/jf304076z.

[71] X. Zhao, R. Chen, Y. Shi, X. Zhang, C. Tian, and D. Xia, "Antioxidant and Anti-Inflammatory Activities of Six Flavonoids from Smilax glabra Roxb," *Molecules*, vol. 25, no. 22, p. 5295, Nov. 2020, doi: 10.3390/molecules25225295.

[72] F. S. Youssef, M. L. Ashour, H. A. El-Beshbishy, A. Ahmed Hamza, A. N. B. Singab, and M. Wink, "Pinoresinol-4- *O* - β -D-glucopyranoside: a lignan from prunes (*Prunus domestica*) attenuates oxidative stress, hyperglycaemia and hepatic toxicity *in vitro* and *in vivo*," *Journal of Pharmacy and Pharmacology*, vol. 72, no. 12, pp. 1830–1839, Nov. 2020, doi: 10.1111/jphp.13358.

[73] A. N. Khan *et al.*, “A Short Review on Glucogallin and its Pharmacological Activities,” *Mini-Reviews in Medicinal Chemistry*, vol. 22, no. 22, pp. 2820–2830, Dec. 2022, doi: 10.2174/1389557522666220513150907.

[74] H.-N. Chu *et al.*, “What Is the Relationship between Antioxidant Efficacy, Functional Composition, and Genetic Characteristics in Comparing Soybean Resources by Year?,” *Antioxidants*, vol. 11, no. 11, p. 2249, Nov. 2022, doi: 10.3390/antiox11112249.

[75] Y. Wang *et al.*, “A New Geniposidic Acid Derivative Exerts Antiaging Effects through Antioxidative Stress and Autophagy Induction,” *Antioxidants*, vol. 10, no. 6, p. 987, Jun. 2021, doi: 10.3390/antiox10060987.

[76] S. Chae *et al.*, “Antioxidant Activity of Isoacteoside from Clerodendron Trichotomum,” *J Toxicol Environ Health A*, vol. 68, no. 5, pp. 389–400, Jan. 2005, doi: 10.1080/15287390590900750.

[77] X.-J. Qu *et al.*, “Protective effects of Salvia plebeia compound homoplantaginin on hepatocyte injury,” *Food and Chemical Toxicology*, vol. 47, no. 7, pp. 1710–1715, Jul. 2009, doi: 10.1016/j.fct.2009.04.032.

[78] X. Han, “Protective effect of naringenin-7-O-glucoside against oxidative stress induced by doxorubicin in H9c2 cardiomyocytes,” *Biosci Trends*, 2012, doi: 10.5582/bst.2012.v6.1.19.

[79] M. Wójciak, M. Feldo, G. Borowski, T. Kubrak, B. J. Płachno, and I. Sowa, “Antioxidant Potential of Diosmin and Diosmetin against Oxidative Stress in Endothelial Cells,” *Molecules*, vol. 27, no. 23, p. 8232, Nov. 2022, doi: 10.3390/molecules27238232.

[80] “The Bioactive Effects of Chicoric Acid As a Functional Food Ingredient”.

[81] H. Wang *et al.*, “Aucubin alleviates oxidative stress and inflammation via Nrf2-mediated signaling activity in experimental traumatic brain injury,” *J Neuroinflammation*, vol. 17, no. 1, p. 188, Dec. 2020, doi: 10.1186/s12974-020-01863-9.

[82] A. Cherkas, S. Holota, T. Mdzinarashvili, R. Gabbianelli, and N. Zarkovic, “Glucose as a Major Antioxidant: When, What for and Why It Fails?,” *Antioxidants*, vol. 9, no. 2, p. 140, Feb. 2020, doi: 10.3390/antiox9020140.

[83] J. Zhu, G. Li, J. Zhou, Z. Xu, and J. Xu, “Cytoprotective effects and antioxidant activities of acteoside and various extracts of Clerodendrum cyrtophyllum Turcz leaves against t-BHP induced oxidative damage,” *Sci Rep*, vol. 12, no. 1, p. 12630, Jul. 2022, doi: 10.1038/s41598-022-17038-w.

[84] “Antioxidant effects and mechanism of silymarin in oxidative stress induced cardiovascular diseases”.

[85] T. Boeing *et al.*, “Antioxidant and anti-inflammatory effect of plumieride in dextran sulfate sodium-induced colitis in mice,” *Biomedicine & Pharmacotherapy*, vol. 99, pp. 697–703, Mar. 2018, doi: 10.1016/j.biopha.2018.01.142.

[86] X. Ao *et al.*, “Extraction, isolation and identification of four phenolic compounds from Pleioblastus amarus shoots and their antioxidant and anti-inflammatory properties in vitro,” *Food Chem*, vol. 374, p. 131743, Apr. 2022, doi: 10.1016/j.foodchem.2021.131743.

[87] Y.-C. Cho, J. Park, and S. Cho, “Anti-Inflammatory and Anti-Oxidative Effects of luteolin-7-O-glucuronide in LPS-Stimulated Murine Macrophages through TAK1 Inhibition and Nrf2 Activation,” *Int J Mol Sci*, vol. 21, no. 6, p. 2007, Mar. 2020, doi: 10.3390/ijms21062007.

[88] K. A. Kang *et al.*, “Luteolin induces apoptotic cell death via antioxidant activity in human colon cancer cells,” *Int J Oncol*, vol. 51, no. 4, pp. 1169–1178, Oct. 2017, doi: 10.3892/ijo.2017.4091.

[89] W. Wang *et al.*, “Efficiency comparison of apigenin-7-O-glucoside and trolox in antioxidative stress and anti-inflammatory properties,” *Journal of Pharmacy and Pharmacology*, vol. 72, no. 11, pp. 1645–1656, Oct. 2020, doi: 10.1111/jphp.13347.

[90] A. Hameed, N. Iqbal, and S. A. Malik, “Effect of d-mannose on antioxidant defense and oxidative processes in etiolated wheat coleoptiles,” *Acta Physiol Plant*, vol. 36, no. 1, pp. 161–167, Jan. 2014, doi: 10.1007/s11738-013-1396-5.

[91] P. Kashyap, D. Shikha, M. Thakur, and A. Aneja, “Functionality of apigenin as a potent antioxidant with emphasis on bioavailability, metabolism, action mechanism and in vitro and in vivo studies: A review,” *J Food Biochem*, vol. 46, no. 4, Apr. 2022, doi: 10.1111/jfbc.13950.

[92] H. Han *et al.*, “Quercetin-3-O-α-L-rhamnopyranoside derived from the leaves of Lindera aggregata (Sims) Kosterm. evokes the autophagy-induced nuclear factor erythroid 2-related factor 2 antioxidant pathway in human umbilical vein endothelial cells,” *Int J Mol Med*, Nov. 2018, doi: 10.3892/ijmm.2018.3976.

[93] A. André, M. Leupin, M. Kneubühl, V. Pedan, and I. Chetschik, “Evolution of the Polyphenol and Terpene Content, Antioxidant Activity and Plant Morphology of Eight Different Fiber-Type Cultivars of Cannabis sativa L. Cultivated at Three Sowing Densities,” *Plants*, vol. 9, no. 12, p. 1740, Dec. 2020, doi: 10.3390/plants9121740.

[94] J.-H. Moon, T. Tsushida, K. Nakahara, and J. Terao, “Identification of quercetin 3- O -β-D-glucuronide as an antioxidative metabolite in rat plasma after oral administration of quercetin,” *Free Radic Biol Med*, vol. 30, no. 11, pp. 1274–1285, Jun. 2001, doi: 10.1016/S0891-5849(01)00522-6.

[95] H. Kırmızıbekmez *et al.*, “Secondary metabolites from the aerial parts of *Sideritis germanicopolitana* and their *in vitro* enzyme inhibitory activities,” *Nat Prod Res*, vol. 35, no. 4, pp. 655–658, Feb. 2021, doi: 10.1080/14786419.2019.1586700.

[96] S. K. Yoon, “Oleuropein as an Antioxidant and Liver Protect,” in *The Liver*, Elsevier, 2018, pp. 323–335. doi: 10.1016/B978-0-12-803951-9.00027-6.

[97] K. Iwai, N. Kishimoto, Y. Kakino, K. Mochida, and T. Fujita, “In Vitro Antioxidative Effects and Tyrosinase Inhibitory Activities of Seven Hydroxycinnamoyl Derivatives in Green Coffee Beans,” *J Agric Food Chem*, vol. 52, no. 15, pp. 4893–4898, Jul. 2004, doi: 10.1021/jf040048m.

[98] Y. Kumarasamy, P. J. Cox, M. Jaspars, L. Nahar, and S. D. Sarker, “Bioactivity of Hirsutanolol, Oregonin and Genkwanin, Isolated from the Seeds of Alnus glutinosa (Betulaceae).”

[99] J. Ho and C.-Y. Hong, “Salvianolic acids: small compounds with multiple mechanisms for cardiovascular protection,” *J Biomed Sci*, vol. 18, no. 1, p. 30, 2011, doi: 10.1186/1423-0127-18-30.

[100] O. Danino, H. E. Gottlieb, S. Grossman, and M. Bergman, “Antioxidant activity of 1,3-dicaffeoylquinic acid isolated from Inula viscosa,” *Food Research International*, vol. 42, no. 9, pp. 1273–1280, Nov. 2009, doi: 10.1016/j.foodres.2009.03.023.

[101] S. Bakir, S. Kamiloglu, M. Tomas, and E. Capanoglu, “Tomato Polyphenolics: Putative Applications to Health and Disease,” in *Polyphenols: Mechanisms of Action in Human Health and Disease*, Elsevier, 2018, pp. 93–102. doi: 10.1016/B978-0-12-813006-3.00009-X.

[102] A. Braca, G. Fico, I. Morelli, F. de Simone, F. Tomè, and N. de Tommasi, “Antioxidant and free radical scavenging activity of flavonol glycosides from different Aconitum species,” *J Ethnopharmacol*, vol. 86, no. 1, pp. 63–67, May 2003, doi: 10.1016/S0378-8741(03)00043-6.

[103] Y. S. Song and C. M. Park, “Luteolin and luteolin-7-O-glucoside strengthen antioxidative potential through the modulation of Nrf2/MAPK mediated HO-1 signaling cascade in RAW 264.7 cells,” *Food and Chemical Toxicology*, vol. 65, pp. 70–75, Mar. 2014, doi: 10.1016/j.fct.2013.12.017.

[104] “Free Radical Scavenging and Antioxidant Activities of Silymarin Components”.

[105] A. Nugroho, H. Heryani, J. S. Choi, and H.-J. Park, “Identification and quantification of flavonoids in Carica papaya leaf and peroxynitrite-scavenging activity,” *Asian Pac J Trop Biomed*, vol. 7, no. 3, pp. 208–213, Mar. 2017, doi: 10.1016/j.apjtb.2016.12.009.

[106] A. Elmann, A. Telerman, H. Erlank, R. Ofir, Y. Kashman, and E. Beit-Yannai, “Achillolide A Protects Astrocytes against Oxidative Stress by Reducing Intracellular Reactive Oxygen Species and Interfering with Cell Signaling,” *Molecules*, vol. 21, no. 3, p. 301, Mar. 2016, doi: 10.3390/molecules21030301.

[107] Y. Wang *et al.*, “Variation in the Main Health-Promoting Compounds and Antioxidant Activity of Different Edible Parts of Purple Flowering Stalks (Brassica campestris var. purpuraria) and Green Flowering Stalks (Brassica campestris var. campestris),” *Plants*, vol. 11, no. 13, p. 1664, Jun. 2022, doi: 10.3390/plants11131664.

[108] H. Parhiz, A. Roohbakhsh, F. Soltani, R. Rezaee, and M. Iranshahi, “Antioxidant and Anti-Inflammatory Properties of the Citrus Flavonoids Hesperidin and Hesperetin: An Updated Review of their Molecular Mechanisms and Experimental Models,” *Phytotherapy Research*, vol. 29, no. 3, pp. 323–331, Mar. 2015, doi: 10.1002/ptr.5256.

[109] A. John and H. Raza, “Azadirachtin Attenuates Lipopolysaccharide-Induced ROS Production, DNA Damage, and Apoptosis by Regulating JNK/Akt and AMPK/mTOR-Dependent Pathways in Rin-5F Pancreatic Beta Cells,” *Biomedicines*, vol. 9, no. 12, p. 1943, Dec. 2021, doi: 10.3390/biomedicines9121943.

[110] D.-W. Kim *et al.*, “Isorhamnetin-3-O-galactoside Protects against CCl_4 -Induced Hepatic Injury in Mice,” *Biomol Ther (Seoul)*, vol. 20, no. 4, pp. 406–412, Jul. 2012, doi: 10.4062/biomolther.2012.20.4.406.

[111] A. Sharma, S. Gupta, S. Chauhan, A. Nair, and P. Sharma, “ASTILBIN: A PROMISING UNEXPLORED COMPOUND WITH MULTIDIMENSIONAL MEDICINAL AND HEALTH BENEFITS,” *Pharmacol Res*, vol. 158, p. 104894, Aug. 2020, doi: 10.1016/j.phrs.2020.104894.

[112] M. Cuendet, O. Potterat, and K. Hostettmann, “Flavonoids and phenylpropanoid derivatives from Campanula barbata,” *Phytochemistry*, vol. 56, no. 6, pp. 631–636, Mar. 2001, doi: 10.1016/S0031-9422(00)00423-4.

[113] M. de la L. Cádiz-Gurrea, D. Pinto, C. Delerue-Matos, and F. Rodrigues, “Olive Fruit and Leaf Wastes as Bioactive Ingredients for Cosmetics—A Preliminary Study,” *Antioxidants*, vol. 10, no. 2, p. 245, Feb. 2021, doi: 10.3390/antiox10020245.

[114] J. Wang *et al.*, “Antitumor, antioxidant and anti-inflammatory activities of kaempferol and its corresponding glycosides and the enzymatic preparation of kaempferol,” *PLoS One*, vol. 13, no. 5, p. e0197563, May 2018, doi: 10.1371/journal.pone.0197563.

[115] F. C. Maiyo, R. Moodley, and M. Singh, “Cytotoxicity, Antioxidant and Apoptosis Studies of Quercetin-3-O Glucoside and 4-(?-D-Glucopyranosyl-1?4-?-L-Rhamnopyranosyloxy)-Benzyl Isothiocyanate from Moringa oleifera,” *Anticancer Agents Med Chem*, vol. 16, no. 5, pp. 648–656, Mar. 2016, doi: 10.2174/1871520615666151002110424.

[116] V. Gayathri Devi, B. N. Rooban, V. Sasikala, V. Sahasranamam, and A. Abraham, “Isorhamnetin-3-glucoside alleviates oxidative stress and opacification in selenite cataract in vitro,” *Toxicology in Vitro*, vol. 24, no. 6, pp. 1662–1669, Sep. 2010, doi: 10.1016/j.tiv.2010.05.021.

[117] E. M. Ryan *et al.*, “Antioxidant properties of citric acid interfere with the uricase-based measurement of circulating uric acid,” *J Pharm Biomed Anal*, vol. 164, pp. 460–466, Feb. 2019, doi: 10.1016/j.jpba.2018.11.011.

[118] Z. Shojaeifard, B. Hemmateenejad, and A. R. Jassbi, “Chemometrics-based LC-UV-ESIMS analyses of 50 Salvia species for detecting their antioxidant constituents,” *J Pharm Biomed Anal*, vol. 193, p. 113745, Jan. 2021, doi: 10.1016/j.jpba.2020.113745.

[119] N. Terahara, I. Konczak, H. Ono, M. Yoshimoto, and O. Yamakawa, “Characterization of Acylated Anthocyanins in Callus Induced From Storage Root of Purple-Fleshed Sweet Potato, Ipomoea batatas L,” *J Biomed Biotechnol*, vol. 2004, no. 5, pp. 279–286, 2004, doi: 10.1155/S1110724304406056.

[120] R. A. Mendes *et al.*, “Evaluation of the antioxidant potential of myricetin 3-O-α-L-rhamnopyranoside and myricetin 4′-O-α-L-rhamnopyranoside through a computational study,” *J Mol Model*, vol. 25, no. 4, p. 89, Apr. 2019, doi: 10.1007/s00894-019-3959-x.

[121] N. P. A, B. A. K, S. Bhattu, S. H. K, G. S. K, and S. G. S, “Antioxidant profiling of C3 quercetin glycosides: Quercitrin, Quercetin 3-β-D-glucoside and Quercetin 3-O-(6”-O-malonyl)-β-Dglucoside in cell free environment,” *Free Radicals and Antioxidants*, vol. 5, no. 2, pp. 90–100, Jun. 2015, doi: 10.5530/fra.2015.2.7.

[122] J. G. Ganzon, L.-G. Chen, and C.-C. Wang, “4- O -Caffeoylquinic acid as an antioxidant marker for mulberry leaves rich in phenolic compounds,” *J Food Drug Anal*, vol. 26, no. 3, pp. 985–993, Jul. 2018, doi: 10.1016/j.jfda.2017.11.011.

[123] X. Ren *et al.*, “Isorhamnetin, Hispidulin, and Cirsimaritin Identified in Tamarix ramosissima Barks from Southern Xinjiang and Their Antioxidant and Antimicrobial Activities,” *Molecules*, vol. 24, no. 3, p. 390, Jan. 2019, doi: 10.3390/molecules24030390.

[124] Li, R. Tsao, R. Yang, C. Liu, H. Zhu, and J. C. Young, “Polyphenolic Profiles and Antioxidant Activities of Heartnut (*Juglans ailanthifolia* Var . *cordiformis*) and Persian Walnut (*Juglans regia* L.),” *J Agric Food Chem*, vol. 54, no. 21, pp. 8033–8040, Oct. 2006, doi: 10.1021/jf0612171.

[125] Y. Ma, Y. Shang, D. Zhu, C. Wang, Z. Zhong, and Z. Xu, “Facile Separation of 5- *O* -Galloylquinic Acid from Chinese Green Tea Extract using Mesoporous Zirconium Phosphate,” *Phytochemical Analysis*, vol. 27, no. 3–4, pp. 153–157, May 2016, doi: 10.1002/pca.2610.

[126] M. Xu, Z. Jin, J.-B. Ohm, P. Schwarz, J. Rao, and B. Chen, “Improvement of the Antioxidative Activity of Soluble Phenolic Compounds in Chickpea by Germination,” *J Agric Food Chem*, vol. 66, no. 24, pp. 6179–6187, Jun. 2018, doi: 10.1021/acs.jafc.8b02208.

[127] F. Topal *et al.*, “Antioxidant activity of taxifolin: an activity–structure relationship,” *J Enzyme Inhib Med Chem*, vol. 31, no. 4, pp. 674–683, Jul. 2016, doi: 10.3109/14756366.2015.1057723.

[128] M. Dosedĕl *et al.*, “Vitamin C—Sources, Physiological Role, Kinetics, Deficiency, Use, Toxicity, and Determination,” *Nutrients*, vol. 13, no. 2, p. 615, Feb. 2021, doi: 10.3390/nu13020615.

[129] H. Kirmizibekmez *et al.*, “Iridoid, phenylethanoid and flavonoid glycosides from Sideritis trojana,” *Fitoterapia*, vol. 83, no. 1, pp. 130–136, Jan. 2012, doi: 10.1016/j.fitote.2011.10.003.

[130] W. Hu *et al.*, “Apigenin-7-O-β- <scp>d</scp> -glucuronide inhibits LPS-induced inflammation through the inactivation of AP-1 and MAPK signaling pathways in RAW 264.7 macrophages and protects mice against endotoxin shock,” *Food Funct*, vol. 7, no. 2, pp. 1002–1013, 2016, doi: 10.1039/C5FO01212K.

[131] T. Esposito *et al.*, “Study on Ajuga reptans Extract: A Natural Antioxidant in Microencapsulated Powder Form as an Active Ingredient for Nutraceutical or Pharmaceutical Purposes,” *Pharmaceutics*, vol. 12, no. 7, p. 671, Jul. 2020, doi: 10.3390/pharmaceutics12070671.

[132] H.-D. Lee, J. H. Kim, Q. Q. Pang, P.-M. Jung, E. J. Cho, and S. Lee, “Antioxidant Activity and Acteoside Analysis of Abeliophyllum distichum,” *Antioxidants*, vol. 9, no. 11, p. 1148, Nov. 2020, doi: 10.3390/antiox9111148.

[133] G. A. L. de Oliveira *et al.*, “Bergenin from Peltophorum dubium: Isolation, Characterization, and Antioxidant Activities in Non-Biological Systems and Erythrocytes,” *Med Chem (Los Angeles)*, vol. 13, no. 6, Aug. 2017, doi: 10.2174/1573406413666170306120152.

[134] A. Dawé *et al.*, “Flavonoids and triterpenes from *Combretum fragrans* with anti-inflammatory, antioxidant and antidiabetic potential,” *Zeitschrift für Naturforschung C*, vol. 73, no. 5–6, pp. 211–219, Apr. 2018, doi: 10.1515/znc-2017-0166.

[135] Y. T. Tung, W. C. Chang, P. S. Chen, T. C. Chang, and S. T. Chang, “Ultrasound-assisted extraction of phenolic antioxidants from *Acacia confusa* flowers and buds,” *J Sep Sci*, vol. 34, no. 7, pp. 844–851, Apr. 2011, doi: 10.1002/jssc.201000820.

[136] C. M. Park and Y.-S. Song, “Luteolin and luteolin-7- *O* -glucoside protect against acute liver injury through regulation of inflammatory mediators and antioxidative enzymes in GalN/LPS-induced hepatitic ICR mice,” *Nutr Res Pract*, vol. 13, no. 6, p. 473, 2019, doi: 10.4162/nrp.2019.13.6.473.

[137] G. Liu *et al.*, “Antioxidant capacity and interaction of endogenous phenolic compounds from tea seed oil,” *Food Chem*, vol. 376, p. 131940, May 2022, doi: 10.1016/j.foodchem.2021.131940.

[138] L. Jin, C. Chen, L. Huang, L. Bu, L. Zhang, and Q. Yang, “Salvianolic acid A blocks vasculogenic mimicry formation in human non-small cell lung cancer via PI3K/Akt/mTOR signalling,” *Clin Exp Pharmacol Physiol*, vol. 48, no. 4, pp. 508–514, Apr. 2021, doi: 10.1111/1440-1681.13464.

[139] K. P. Funh *et al.*, “Lithospermic acid B as an antioxidant-based protector of cultured ventricular myocytes and aortic endothelial cells of rabbits,” *Life Sci*, vol. 53, no. 12, pp. PL189–PL193, Jan. 1993, doi: 10.1016/0024-3205(93)90129-Q.

[140] Q. Li, P. Zhang, and Y. Cai, “Genkwanin suppresses MPP+-induced cytotoxicity by inhibiting TLR4/MyD88/NLRP3 inflammasome pathway in a cellular model of Parkinson’s disease,” *Neurotoxicology*, vol. 87, pp. 62–69, Dec. 2021, doi: 10.1016/j.neuro.2021.08.018.

[141] J. Yan *et al.*, “Rhoifolin Ameliorates Osteoarthritis via Regulating Autophagy,” *Front Pharmacol*, vol. 12, May 2021, doi: 10.3389/fphar.2021.661072.

[142] N. Li, M. Xu, M. Wu, and G. Zhao, “Cinnamtannin A2 protects the renal injury by attenuates the altered expression of kidney injury molecule 1 (KIM-1) and neutrophil gelatinase-associated lipocalin (NGAL) expression in 5/6 nephrectomized rat model,” *AMB Express*, vol. 10, no. 1, p. 87, Dec. 2020, doi: 10.1186/s13568-020-01022-6.

[143] J. Choi, H. J. Kang, S. Z. Kim, T. O. Kwon, S.-I. Jeong, and S. il Jang, “Antioxidant effect of astragalin isolated from the leaves of Morus alba L. against free radical-induced oxidative hemolysis of human red blood cells,” *Arch Pharm Res*, vol. 36, no. 7, pp. 912–917, Jul. 2013, doi: 10.1007/s12272-013-0090-x.

[144] L. S. Frota *et al.*, “Antioxidant and anticholinesterase activities of amentoflavone isolated from *Ouratea fieldingiana* (Gardner) Engl. through *in vitro* and chemical-quantum studies,” *J Biomol Struct Dyn*, pp. 1–11, Dec. 2021, doi: 10.1080/07391102.2021.2017353.

[145] M. Imran *et al.*, “Kaempferol: A Key Emphasis to Its Anticancer Potential,” *Molecules*, vol. 24, no. 12, p. 2277, Jun. 2019, doi: 10.3390/molecules24122277.

[146] Y. Gao *et al.*, “Antioxidant Activity Evaluation of Dietary Flavonoid Hyperoside Using Saccharomyces Cerevisiae as a Model,” *Molecules*, vol. 24, no. 4, p. 788, Feb. 2019, doi: 10.3390/molecules24040788.

[147] Y. Deng *et al.*, “Kaempferol-3-O-Glucuronide Ameliorates Non-Alcoholic Steatohepatitis in High-Cholesterol-Diet-Induced Larval Zebrafish and HepG2 Cell Models via Regulating Oxidation Stress,” *Life*, vol. 11, no. 5, p. 445, May 2021, doi: 10.3390/life11050445.

[148] M. A. Aderogba, L. J. Mcgaw, A. O. Ogundaini, and J. N. Eloff, “Antioxidant activity and cytotoxicity study of the flavonol glycosides from ***Bauhinia galpinii*** ,” *Nat Prod Res*, vol. 21, no. 7, pp. 591–599, Jun. 2007, doi: 10.1080/14786410701369557.

[149] T. Kassa, J. G. Whalin, M. P. Richards, and A. I. Alayash, "Caffeic acid: an antioxidant with novel antisickling properties," *FEBS Open Bio*, Sep. 2021, doi: 10.1002/2211-5463.13295.

[150] V. Morocho, L. P. Valarezo, D. A. Tapia, L. Cartuche, N. Cumbicus, and G. Gilardoni, "A Rare Dirhamnosyl Flavonoid and Other Radical-Scavenging Metabolites from *Cynophalla mollis* (Kunth) J. Presl and *Colicodendron scabridum* (Kunt) Seem. (Capparaceae) of Ecuador," *Chem Biodivers*, vol. 18, no. 8, Aug. 2021, doi: 10.1002/cbdv.202100260.

[151] S. P. Kamdi, A. Raval, and K. T. Nakhate, "Phloridzin attenuates lipopolysaccharide-induced cognitive impairment via antioxidant, anti-inflammatory and neuromodulatory activities," *Cytokine*, vol. 139, p. 155408, Mar. 2021, doi: 10.1016/j.cyto.2020.155408.
